# Characterization of a Silver Vinylcarbene Intermediate in Carbene–Alkyne Metathesis and Its Concerted C(sp^2^)–H Bond Insertion

**DOI:** 10.1021/jacs.5c19806

**Published:** 2026-01-12

**Authors:** Àlex Díaz-Jiménez, Roger Monreal-Corona, Arijit Saha, Andrea Álvarez-Núñez, Anna Company, Teodor Parella, Pedro J. Pérez, Ana Caballero, Anna Roglans, Albert Poater, Anna Pla-Quintana

**Affiliations:** † Institut de Química Computacional i Catàlisi (IQCC) and Departament de Química, 16738 Universitat de Girona (UdG), Facultat de Ciències, C/Maria Aurèlia Capmany, 69, Girona 17003, Catalunya, Spain; ‡ Servei de Ressonància Magnètica Nuclear, Facultat de Ciències i Biociències, 16719 Universitat Autònoma de Barcelona, Cerdanyola del Vallès 08193, Catalunya, Spain; § Laboratorio de Catálisis Homogénea, Unidad Asociada al CSIC, CIQSO-Centro de Investigación en Química Sostenible and Departamento de Química, 16743 Universidad de Huelva, Huelva 21007, Spain

## Abstract

Herein we present compelling evidence of the intervention of electrophilic silver vinylcarbenes in the carbene-alkyne metathesis (CAM) reaction, leading to a pivotal C­(sp^2^)-H insertion process. The delicate equilibrium between the stability and reactivity of transient species is crucial for efficient detection. Through meticulous mechanistic exploration, we unveil that the mechanism for the C­(sp^2^)-H insertion hinges on the substitution pattern. Indeed, it departs from the previously documented stepwise mechanism (involving Wheland intermediates), and it follows a concerted route, quite uncommon for this transformation.

## Graphical abstract

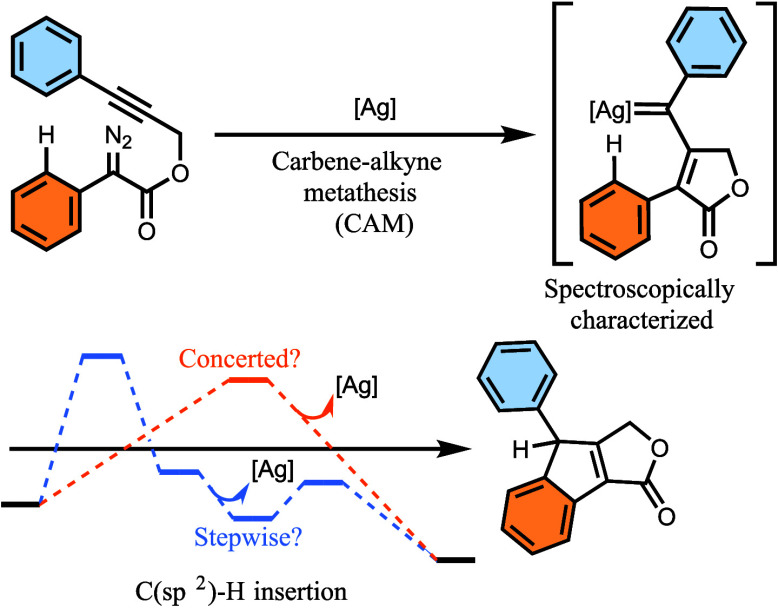

## Introduction

Transition metal carbene complexes are highly versatile reactive intermediates that enable key transformations in organic synthesis. Among them, carbene transfer reactions to C–H bonds^1^ have revolutionized C–C bond forming methodologies, including the regio- and enantioselective modification of the low reactive bonds of alkanes C_*n*_H_2*n*+2_.^2,3^ Silver carbenes are usually more electrophilic than their copper counterparts due to their weaker M-C σ and π bonds.^4^ This provides obvious advantages to their reactivity, such as the potential for insertion into the C–H bond of methane,^5^ but also makes the isolation of these reactive species very challenging.

Indeed, apart from silver-NHC carbene complexes,^6^ to the best of our knowledge, only two electrophilic silver carbenes have been characterized to date. Straub et al.^7^ isolated and characterized, through X-ray diffraction and spectroscopic techniques, a silver carbene formed upon reaction of dimesityl diazomethane and a silver NHC complex (Scheme 1a). Although the reactive carbene site is highly shielded by the crowded NHC ligand, the complex is highly fragile, and no carbene transfer reactivity has been reported for this complex.

**Scheme 1:**
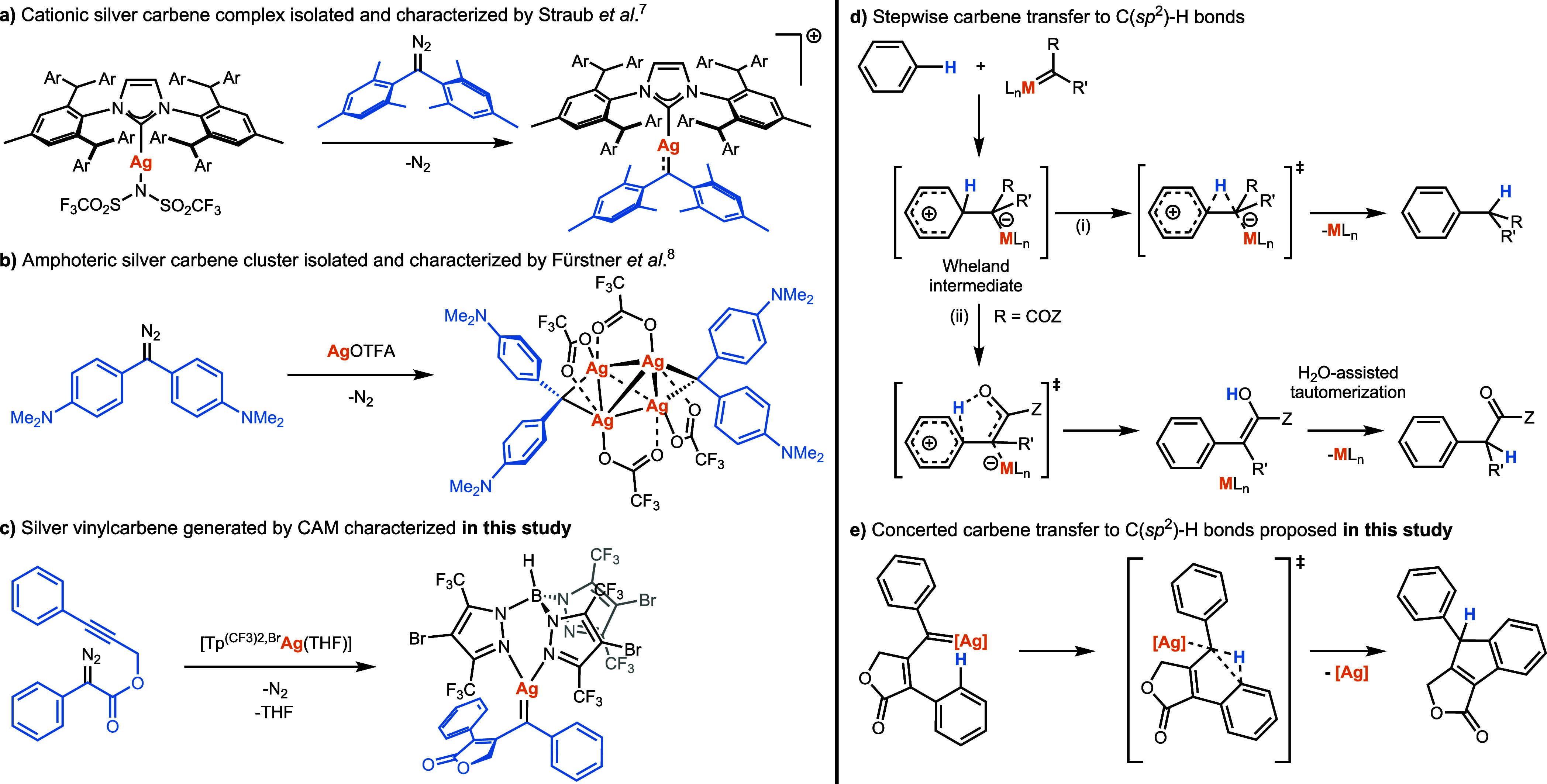
Left. Silver-Carbene Complexes Described to Date (a,b) and Silver Vinylcarbene Characterized in This Study (c) Right. Proposed Mechanisms for Stepwise Carbene Transfer to C­(sp^2^)-H Bonds (d) and Concerted Mechanism Proposed in This Study (e)

Fürstner et al.^8^ also succeeded in isolating a silver trimer and a silver tetramer cluster species upon the reaction of di­(*p*-(dimethylamino)­phenyl) diazomethane and silver trifluoroacetate, both comprising the same bridging μ-carbene entity (for the structure of the tetrameric cluster see Scheme 1b). These clusters exhibit some electrophilic behavior as they afford azines by reaction with a diazo derivative, imines by insertion into the N–H of benzylamine and subsequent oxidation, and participate in cross-coupling reactions with PhMgBr. Due to their amphoteric nature, the complexes also showed modest nucleophilic behavior by acting as carbene transfer agents. However, neither the trimeric nor the tetrameric clusters could cyclopropanate styrene or insert into C–H bonds, either due to the mesomeric effect of the distal NMe_2_ substituent or the assembly into an aggregate.

On the other hand, metal vinylcarbenes formed upon carbene-alkyne metathesis (CAM)^9^ have remained elusive so far. The reactivity of these intermediates, as initially reported by Padwa^10^ and Hoye,^11^ has enormous potential for the construction of polycyclic frameworks,^12,13^ especially if control over their carbenic and vinylogous reactivity is achieved.

As part of our investigations^9b,12,14^ in the use of silver-based catalysts for CAM reactions, we focused on the detection of relevant silver-carbene intermediates in such transformations. Herein we provide, for the first time, spectroscopic evidence for the involvement of electrophilic silver vinylcarbenes in the carbene-alkyne metathesis reaction (Scheme 1c). Additionally, we demonstrate that the transfer reaction of such silver vinylcarbenes to the C­(sp^2^)-H bond diverges from the traditionally accepted stepwise mechanism (Scheme 1d),^15^ proceeding instead through a concerted pathway (Scheme 1e). Our findings provide insights into the electronic and steric factors dictating the choice between these two pathways.

## Results and Discussion

### Silver Catalysts for the CAM/C­(sp^2^)-H Insertion Cascade Reaction

Revisiting the literature, we identified propargyl aryldiazoacetates **1** (Scheme 2a), previously reported by Padwa^16^ and Doyle,^17^ as a powerful platform for the ready generation of a metal vinylcarbene and subsequent study of its transfer to C­(sp^2^)-H bonds. These compounds are generated by esterification of phenylacetic acid derivatives and propargyl alcohols followed by diazotization. Upon treatment with an appropriate transition metal complex,^16,17^ metalocarbene **2** is generated which readily engages in a carbene-alkyne metathesis (CAM) furnishing metal vinylcarbene **3**. This in situ generated species can insert into the C­(sp^2^)-H bond of the phenylacetic moiety leading to indene fused product **4**, in a process that does not compete with the Buchner reaction for steric reasons.^18^

**Scheme 2:**
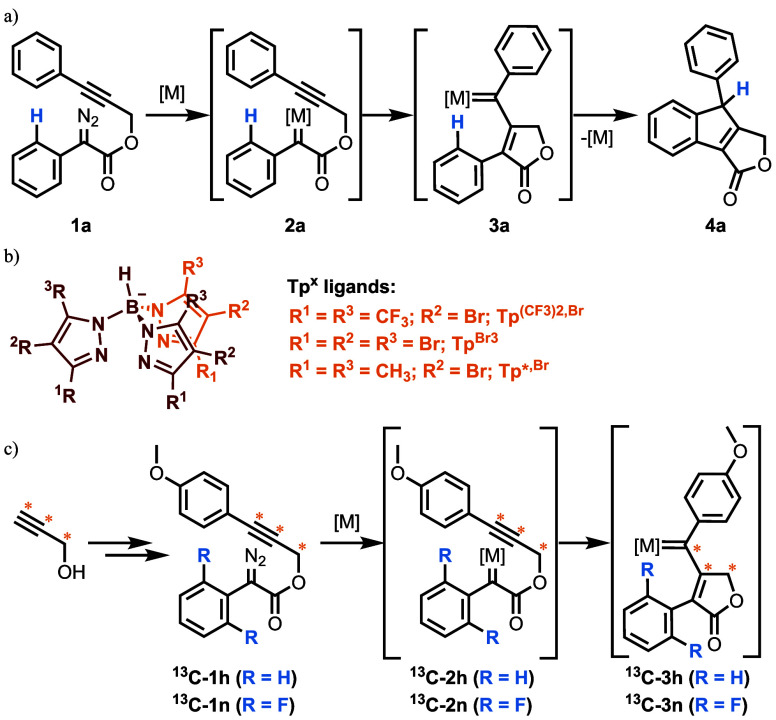
Carbene-Alkyne Metathesis/C­(sp^2^)-H Insertion Cascade in Propargyl Aryldiazoacetates (a); Trispyrazolylborate Ligands Used in This Study (b); ^13^C Labelled Substrates Used in the NMR Detection of the Vinylcarbene Intermediate (c)[Fn s2fn1]

a
^13^C-labelled carbons are marked with asterisks.

We decided to verify the use of silver toward the cascade sequence shown in Scheme 2a, employing silver catalysts containing trispyrazolylborate ligands (Scheme 2b). These Tp^x^AgL complexes have been used as catalysts for the functionalization of poorly reactive carbon–hydrogen bonds,^3b^ and more recently in cascade reactions involving the carbene-alkyne metathesis (CAM).^14^ In a first attempt, we used 3-phenylprop-2-yn-1-yl-2-diazo-2-phenylacetate (**1a**) as model substrate and treated it with [Tp^(CF3)2,Br^Ag­(THF)] (5 mol %) as the catalyst in dichloromethane at room temperature (Scheme 3). The reaction afforded the expected indene fused product **4a** in quantitative yield, as confirmed by spectroscopic techniques and by X-ray diffraction (see Supporting Information).^19^

**Scheme 3:**
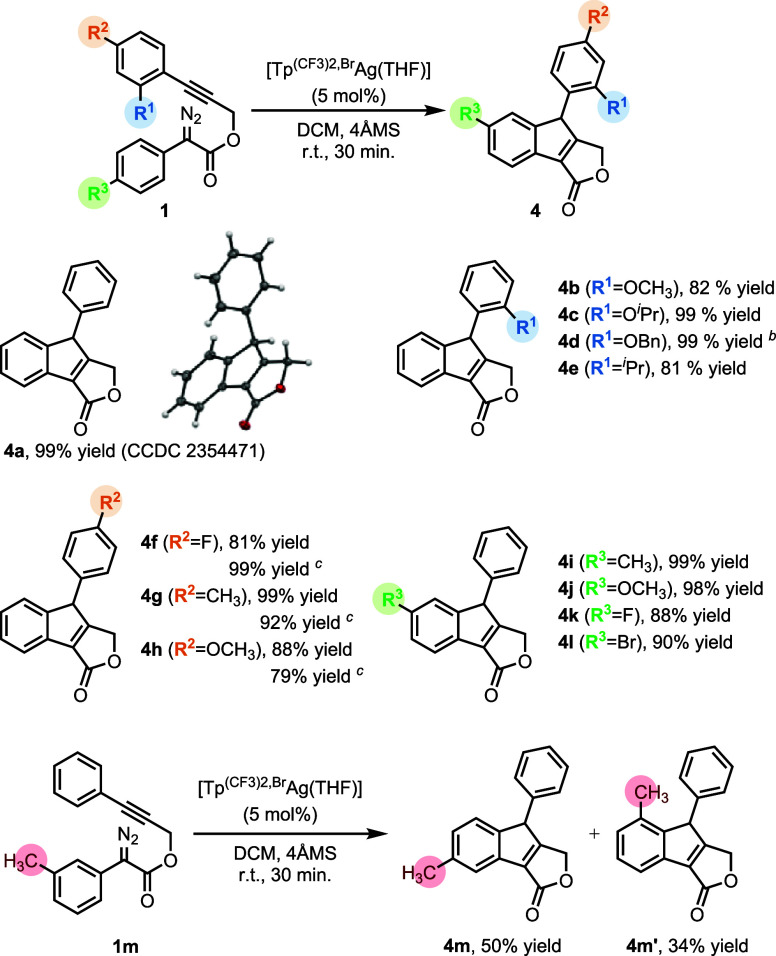
Scope of the CAM/C­(sp^2^)–H Insertion Cascade^a^

a Unless otherwise noted, we report isolated yields for reactions carried out with 0.07 mmol of **1** in DCM ([**1**] = 4.7 mM), at room temperature for 30 min. ^*b*^ Reaction run for 1.5 h. ^*c*^ Using [Tp^Br3^Ag]_2_ as the catalyst.

To assess the generality of this protocol, we prepared a set of 13 substrates, from which information about the chemoselectivity of the reaction and the electronic and steric effects on the reactivity was collected. The reaction worked with excellent yields for all the substrates (Scheme 3). The effect of substituents at the *ortho*-position of the phenylpropynyl moiety (R^1^, Scheme 3) was initially evaluated. Very good to excellent yields were obtained regardless of the electron-donating or moderate electron-withdrawing character of the substituents. Noteworthy, in all cases the carbene transfer was selective to the C­(sp^2^)-H in the phenyl ring of the phenylacetic moiety and no competitive carbene transfer into the primary (**4b** and **4e**), secondary (**4d**) or tertiary (**4c**) C­(sp^3^)-H in the R^1^ substituent was observed. Diazo compounds with a *para* substituent in the phenylpropyl moiety (R^2^, Scheme 3) were also suitable substrates for the reaction with a slightly decreased yield both for electron-withdrawing (**4f**) and electron-donating (**4h**) substituents. Use of [Tp^Br3^Ag]_2_ was also studied improving the yield for **4f** but decreasing it for **4g** and **4h**.

We finally investigated the effect of substituents in the phenylacetic moiety using the fluorinated catalyst. When substituents were introduced in the *para* position (R^3^, Scheme 3), excellent yields were obtained, with only a very slight decrease observed with halogen substituents (**4k** and **4l**). When a methyl group was introduced in the *meta* position (**1m**, Scheme 3), a mixture of two diastereoisomers, **4m** and **4m′**, was obtained. The formation of **4m**, which is less sterically hindered, was slightly favored. Overall, the use of these silver catalyst provided the same type of compounds previously reported by Padwa,^16^ and Doyle.^17^

### Detection of the Silver-Carbene Species

With a very clean transformation in hand and motivated by the interest in characterizing carbene compounds en route to carbene transfer, we attempted the detection of such species in our reaction. To pursue this objective, we initially mixed diazo compound **1a** with 1.1 equiv of [Tp^(CF3)2,Br^Ag­(THF)] in CDCl_3_ at −40 °C, and NMR spectra were recorded. Even though NMR signals of a transient species were observed, this compound proved too short-lived to allow reliable characterization under optimal sensitivity conditions. To address this limitation, we designed and synthesized the selective ^13^C-labeled ^**13**^ **C-1n** substrate (Scheme 2c) from fully ^13^C-labeled propargyl alcohol. This substrate presents key features to entrap the prospective silver-carbene species: (1) three ^13^C-labels, including one at the carbene-forming carbon to enhance NMR sensitivity; (2) two *ortho*-fluorine atoms on the phenylacetic acid moiety to block the final step of the reaction; and (3) a methoxy substituent on the alkyne-bound phenyl ring to aid NMR signal discrimination in the aromatic region. Reaction of ^**13**^ **C-1n** with 1.1 equiv of [Tp^(CF3)2,Br^Ag­(THF)] in anhydrous, degassed CDCl_3_ at −40 °C produced an emerald-green solution containing the silver vinylcarbene species ^**13**^ **C-3n** (Scheme 2c), which turned out to be stable enough at this temperature to be thoroughly characterized. A key diagnostic feature was the downfield ^13^C NMR resonance at 311.4 ppm, which was assigned to the carbene carbon (Figure 1A). This deshielded shift far exceeds the typical 160–220 ppm range for NHC–Ag complexes with σ-donor single bonds, confirming the electrophilic Ag=C double bond nature.^7^ This signal was expected to appear as two overlapping doublets of doublets with nearly identical chemical shifts and intensities, since each carbon couples to the attached silver isotope (^109^Ag (48.2%) or ^107^Ag (51.8%)) and to the adjacent ^13^C-labeled carbon atom. The experimentally observed signal appears as a broad doublet with partially resolved carbon–silver coupling constants estimated at approximately 215 Hz for ^1^ *J*_13C–109Ag_ and 187 Hz for ^1^ *J*_13C–107Ag_, in agreement with those reported for Straub’s complex (202 and 175 Hz, respectively).^7^ The ^1^ *J*_13C–13C_ coupling to the neighboring labeled carbon was determined to be 34 Hz, as measured from the adjacent well-resolved ^13^C signal at 173.8 ppm.

**Figure 1:**
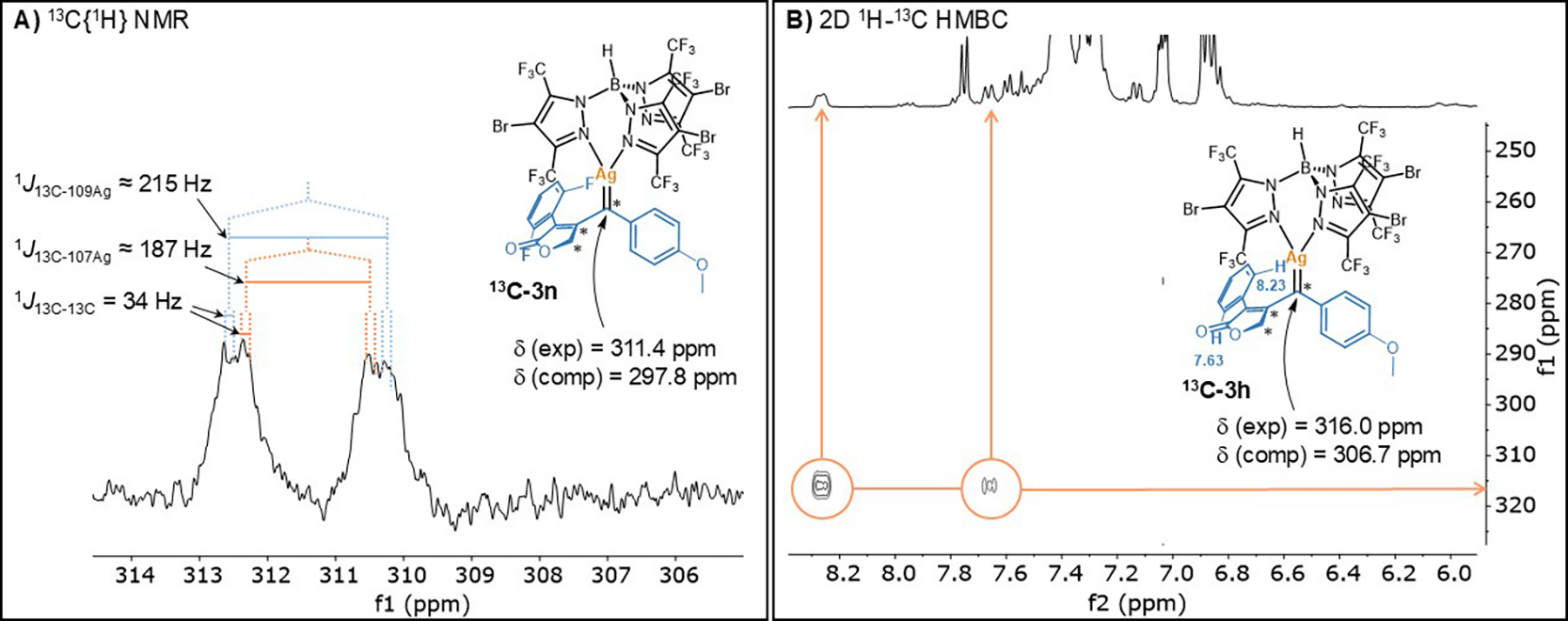
Spectroscopic signatures of silver vinylcarbene species. (A) ^13^C­{^1^H} NMR (CDCl_3_, 100 MHz, 233 K) resonance of the carbene carbon in ^**13**^ **C-3n** (δ = 311.4 ppm). (B) 2D ^1^H–^13^C HMBC (CDCl_3_, 400 MHz, 233 K) cross-peak of ^**13**^ **C-3h** showing strong three-bond correlations between the carbene carbon (δ = 316.0 ppm) and aromatic protons (δ = 8.23, 7.63 ppm). ^13^C-labeled carbons are marked with asterisks.

Analogous NMR experiments were performed with substrate ^**13**^ **C-1h** lacking the two blocking fluorine atoms. Experimental evidence for the existence of the silver vinylcarbene ^**13**^ **C-3h** was obtained by the observation of strong cross-peaks at characteristic 316.0 ppm in the 2D ^1^H–^13^C HMBC spectrum (Figure 1B). These cross-peaks correspond to key three-bond correlations between the vinylcarbene carbon and the aromatic protons of the methoxyphenyl ring at 8.23 and 7.63 ppm, similar to the correlations observed for the fluorine-blocked system ^**13**^ **C-3n**. ^**13**^ **C-3h** underwent a relatively fast conversion to products even at −40 °C, preventing optimal acquisition of the more time-consuming 1D ^13^C NMR spectrum.

To support the spectroscopic assignment, the ^13^C NMR chemical shifts of the vinylcarbene carbon were computed using ORCA (see the Supporting Information for computational details). The protocol was first validated against literature data for silver carbene complexes reported by Fürstner^8^ (δ = 210.6 ppm; exp. 221.4 ppm) and Straub^7^ (δ = 336.7 ppm; exp. 359.3 ppm), showing excellent agreement and confirming the reliability of the method. For the present systems, calculations predicted chemical shifts of the vinylcarbene carbon at 306.7 ppm for **3h**, experimentally observed at 316.0, and 297.8 ppm for the fluorine-blocked **3n**, experimentally detected at 311.4 ppm. Overall, the combination of spectroscopic and computational evidence strongly supports^20^ the assignment of the observed species as silver vinylcarbene compounds.

The electronic properties of the trapped silver vinylcarbene species **3n** were further examined. UV–vis spectroscopy in chloroform at −40 °C revealed a broad absorption band at 622 nm, assigned to excitation from the HOMO—an antibonding σ orbital arising from interaction between the singlet carbene and a metal d orbital—to the LUMO, primarily the vacant p orbital of the carbene carbon, as determined by DFT calculations. To further support this assignment, the UV–vis spectrum of **3n** was simulated using TD-DFT calculations, which predicted the corresponding electronic transition at 607 nm, in good agreement with the observed absorption centered at 622 nm. This band is hypsochromically shifted relative to Straub’s complex (742 nm),^7^ indicating a larger HOMO–LUMO gap. The results suggest that the HOMO is stabilized due to reduced Ag→C backdonation, while the LUMO is slightly destabilized by increased π-acceptor character of the carbene carbon. The weaker metal–to–carbene π interaction resulting in greater orbital separation imparts a more electrophilic carbene center, accounting for the higher reactivity of the vinylcarbene complex **3** under study.

### Computational Mechanistic Studies

To gain additional information about this first spectroscopic evidence for the formation of a vinylcarbene in carbene-alkyne metathesis reactions, we performed density functional theory (DFT) calculations at the B3LYP-D3/Def2TZVP-SDD-SMD­(CH_2_Cl_2_)//BP86-D3/Def2SVP-SDD level of theory using the Gaussian16 software package, as in our previous related study,^14^ where this method was benchmarked and validated for similar substrate–catalyst systems. Figure 2 shows the pathway for the overall transformation of **1a**. The mechanism for this transformation starts with the formation of the donor–acceptor silver carbene **C** through a barrier of 12.2 kcal/mol. Subsequently, a nucleophilic attack of the alkyne onto the carbenic carbon induces a 5-*exo*-dig cyclization, yielding zwitterionic vinyl cationic species **D** in an exergonic step (19.7 kcal/mol) with an energy barrier of 2.2 kcal/mol. Next, the nucleophilic attack of the negatively charged silver atom to the carbocation leads to the formation of silver η^3^-vinylcarbene **E** surpassing a kinetic barrier of 11.0 kcal/mol. At this point, a rearrangement occurs, resulting in the formation of the silver η^1^-vinylcarbene **F** with an associated energy barrier of only 0.4 kcal/mol.

**Figure 2:**
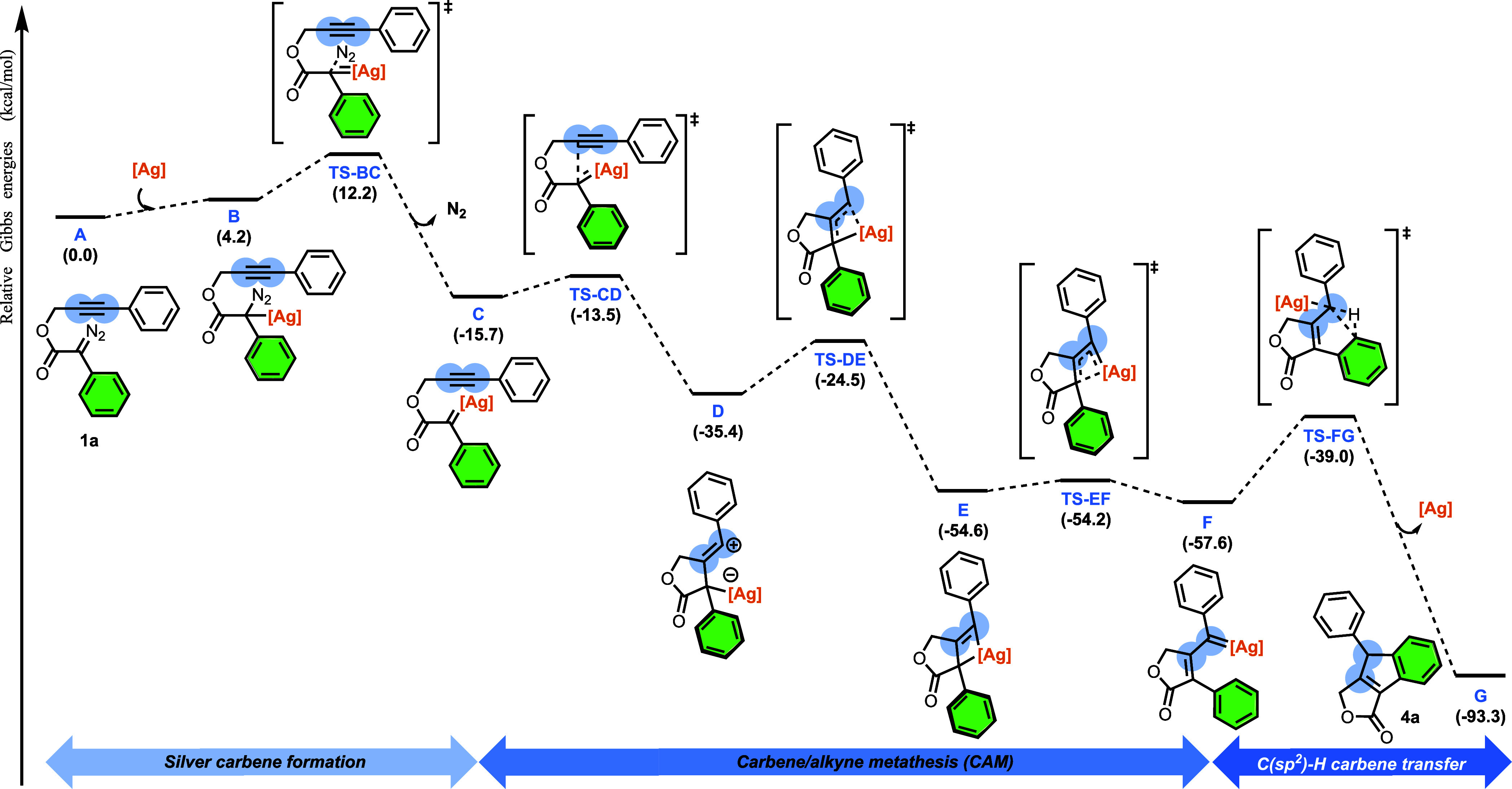
Gibbs energy profile (in kcal/mol) of the silver-catalyzed CAM cascade reaction of **1a** ([Ag] = [Tp^(CF3)2,Br^Ag]).

Then, the carbene transfer takes place with a concerted C–C bond formation and C–H bond activation via **TS-FG** through a barrier of 18.6 kcal/mol, yielding the final scaffold in a highly exergonic process. This pathway reinforces the assignment of the detected intermediate as **3a**, as it precedes the rate-determining step of the reaction. But a second very important point emerges from these results: it postulates a concerted synchronous pathway^21^ for the insertion of the carbene moiety into the C­(sp^2^)-H bond, at variance with previous reports proposing a stepwise pathway.^15^ In fact, precedents for a concerted insertion are limited to one case reported by Xu et al.^22^ using rhodium catalysis.

Indeed, the insertion of a carbene into C­(sp^2^)-H bonds is generally postulated to occur in a stepwise manner,^15^ with the formation of the C–C bond preceding the formation of the C–H bond. The C–C bond formation step involves the electrophilic addition of the transition metal carbene intermediate to the aromatic ring, resulting in a Wheland intermediate (see Scheme 1d). The picture is less specific for the second step. A [1,2]-H migration step^23^ has been postulated for reactions involving donor–donor carbenes. Alternatively, for metal-carbenes including (at least) an acceptor substituent, a proton transfer to the oxygen of the carbonyl adjacent to the carbene carbon forming an enol that tautomerizes to the keto form^24^ has been proposed (Scheme 1d). Despite our efforts, the transition state leading to a Wheland intermediate could not be located in the mechanistic pathway described in Figure 2.

To investigate whether concerted C­(sp^2^)-H insertion is a specific mechanistic feature of donor–donor vinylcarbenes, we ran DFT calculations for the C­(sp^2^)-H insertion step for a series of substrates with different substituents. We initiated our analysis by studying three different substituents in the *para* position of the phenylpropynyl ring (R^2^ = CH_3_, I, CH_3_O). In these cases, both concerted and stepwise transition states could be located (see Figure 3, for substrate **1g** with R^2^ = CH_3_).

**Figure 3:**
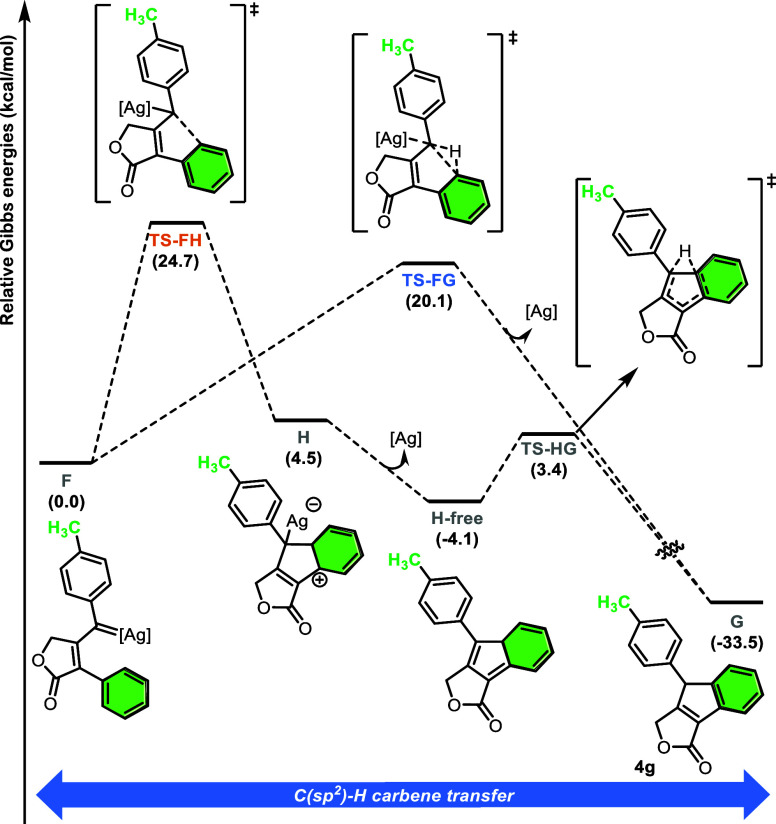
Gibbs energy profile (in kcal/mol) of the C­(sp^2^)-H carbene transfer for the formation of **4g** ([Ag] = [Tp^(CF3)2,Br^Ag]).

For the stepwise C­(sp^2^)-H carbene transfer, the reaction proceeds through electrophilic addition of the carbene to the arene, leading to intermediate **H**, the so-called Wheland intermediate. Subsequently silver decoordination yields 3*H*-indene intermediate **H-free** in an exergonic process. From there, a 1,2-H shift occurs, recovering the aromaticity of the arene ring and yielding the final product **G**. The concerted pathway (**F** → **TS-FG** →**G**) corresponds to that shown in Figure 2. The rate-determining step (rds) for the overall transformation lies in the C­(sp^2^)-H carbene transfer, both in the concerted and stepwise pathways. In the former, the rds is the C–H insertion (**TS-FG**) whereas in the latter the rds is the electrophilic addition of the carbene to the arene (**TS-FH**) (barriers compared in Figure 4b–d). In the three cases studied in this first set (R^2^ = CH_3_, F, CH_3_O), the pathway with the lowest energy corresponded to the concerted mechanism, irrespective of the electron-donating or electron-withdrawing nature of the substituents present in the aromatic ring.

**Figure 4:**
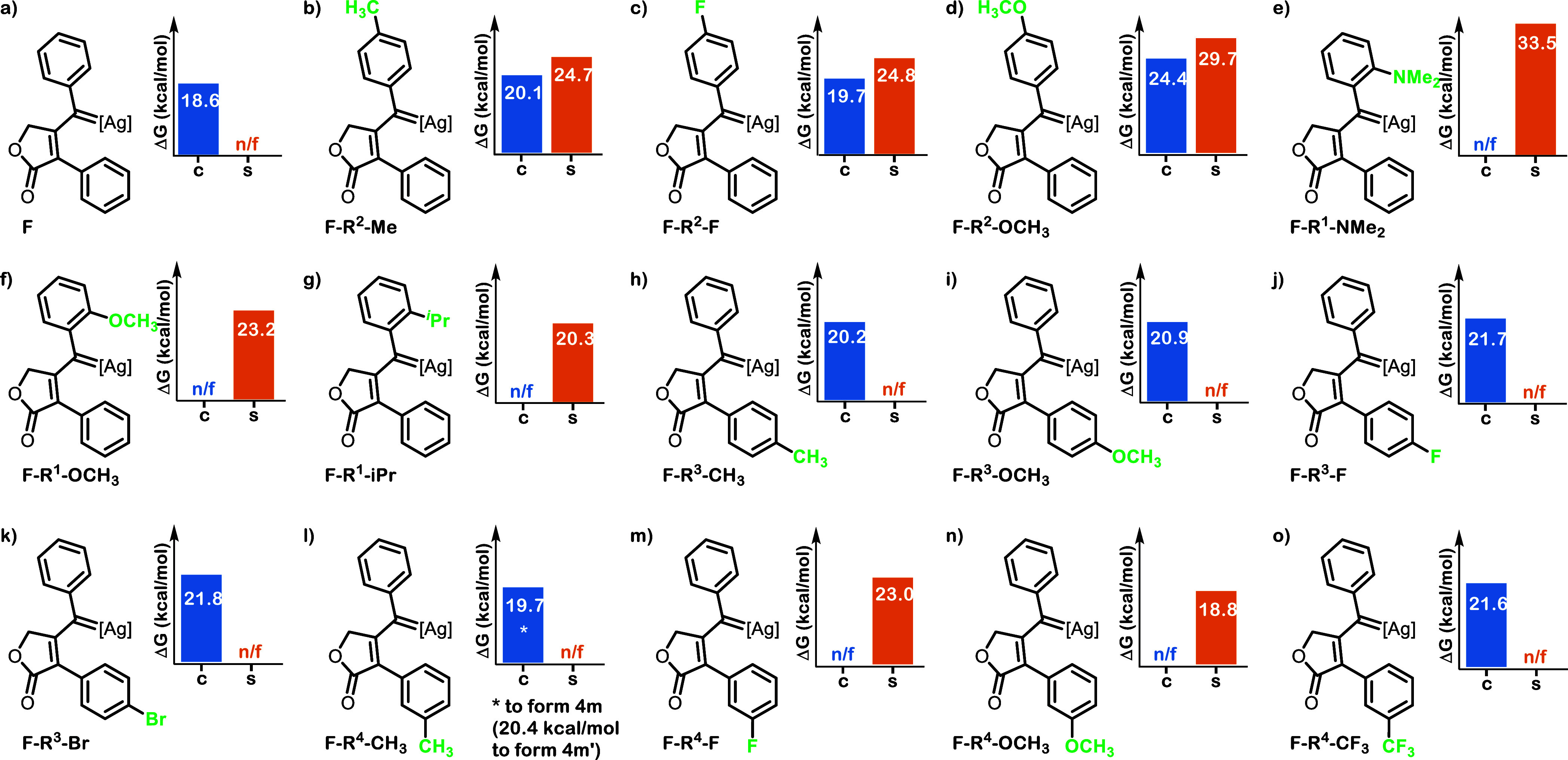
Comparison of the calculated Δ*G^⧧^* values for concerted (c, in blue) and stepwise (s, in orange) pathways in a series of substrates with different substituents ([Ag] = [Tp^(CF3)2,Br^Ag]); Despite our efforts some barriers could not be located and are indicated as *n*/*f* = not feasible).

To validate the generality of our results, we extended our analysis to include calculations using the [Tp^Br3^Ag] core which has proven to be effective for this transformation (Scheme 3). We computed barriers for both mechanisms for substrates featuring F, Me and MeO *para* substituents in the phenylpropynyl moiety (see Supporting Information for the values). Across all three cases examined, our results are consistent with the ones obtained for the [Tp^(CF3)2,Br^Ag] core, reaffirming the predominance of the concerted mechanism.

We then evaluated the effect of NMe_2_, OCH_3_ and ^i^Pr substituents in the *ortho* position of the phenylpropynyl moiety (R^1^) with the [Tp^(CF3)2,Br^Ag] core. In these three cases studied, only the transition state for the stepwise pathway could be located (Figure 4e–g). Notably, for the substrate bearing the dimethylamino moiety, C­(sp^2^)-H insertion would proceed in a stepwise manner. However, the barrier is notably high at 33.5 kcal/mol, nearly 14 kcal/mol higher than the pathway leading to the N–C­(sp^3^)-H insertion previously reported by us,^14^ consistent with the fact that C­(sp^2^)-H insertion was never experimentally observed in this substrate.

To determine if steric factors were responsible for the prevalence of the stepwise mechanism in the substrates bearing substituents in the *ortho* position, we examined the structures of intermediates **F**. The analysis showed that the phenyl ring flanking the carbene adopts a coplanar orientation with the (empty) 2*p* carbene orbital. However, for *o*-iPr (Figure 4g), *o*-NMe_2_ (Figure 4e), *p*-CH_3_ (Figure 4b) and *o*-OCH_3_ (Figure 4f), the phenyl ring is slightly twisted (with the degree of twisting decreasing in the indicated order), and this trend does not correlate with the differences observed. Conversely, a detailed analysis of **TS-FG** and **TS-FH** structures showed that the steric hindrance of the *ortho* substituents impedes the proper approximation of the C–H bond to the carbenic carbon towards the concerted pathway. This determines the prevalence of the stepwise mechanism in the *ortho*-substituted substrates.

Next, we examined the effect of substituents in *para* position in the phenylacetic ring (R^3^). The mechanism for substrates bearing CH_3_, CH_3_O, F, and Br substituents was determined to be always concerted irrespective of the electronic nature of the substituents (Figure 4h–k). On the other hand, and not unexpectedly, when the substituents were introduced in *meta* position, different outcomes were observed depending on the type of substituent.^25^ In the case of a CH_3_ or CF_3_ group (Figure 4l and o, respectively), only the concerted insertion was operative. Conversely, when F or CH_3_O groups were studied (Figure 4m and n, respectively), exclusive stepwise insertion was observed. We hypothesize that the presence of lone pairs of electrons in these substituents provides stabilization through mesomeric effects to the Wheland intermediate to favor the stepwise mechanism (see Supporting Information).^26^

The observation of singular mechanistic features for donor–donor metal carbenes is not unprecedented. For C­(sp^3^)–H bonds, mechanistic studies support in most of the cases a concerted mechanism as initially proposed by Nakamura et al.^27^ However, in recent examples by Shaw, Tantillo et al.^28^ using rhodium and by our group using silver,^14^ an alternative, stepwise C–H insertion mechanism involving a zwitterionic intermediate has been proposed.

## Conclusions

In summary, herein we provide experimental proof of the intervention of electrophilic silver vinylcarbenes in the carbene-alkyne metathesis (CAM) reaction, culminating in a C­(sp^2^)-H insertion process. The delicate balance between a transient species that is stable yet reactive enough to be significant for catalysis allowed for the unprecedented spectroscopic characterization of a silver vinylcarbene. Furthermore, our comprehensive mechanistic investigation reveals that although the literature has adopted a stepwise carbene C­(sp^2^)–H bond insertion as the norm, such reaction can also proceed in a concerted synchronous manner. In the case of the studied silver vinylcarbenes, the most favorable mechanism is concerted, and only in the case of substituents imparting significant steric hindrance or mesomeric stabilization of the Wheland intermediate it does proceed via a stepwise mechanisms.

## Supplementary Material





## Notes

Experimental procedures, compound characterization data including NMR spectra for all compounds and crystallographic data (PDF)
XYZ Cartesian coordinates, energies, and vibrational frequencies of all optimized structures (XYZ)

## References

[1] He Y., Huang Z., Wu K., Ma J., Zhou Y.-G., Yu Z. (2022). Recent Advances in Transition-Metal-Catalyzed Carbene Insertion to
C-H Bonds. Chem. Soc. Rev..

[2] Liao K., Yang Y.-F., Li Y., Sanders J. N., Houk K. N., Musaev D. G., Davies H. M. L. (2018). Design of Catalysts
for Site-Selective and Enantioselective Functionalization of Non-Activated
Primary C–H Bonds. Nat. Chem..

[3] Liu Z., Cao S., Yu W., Wu J., Yi F., Anderson E. A., Bi X. (2020). Site-Selective C–H
Benzylation
of Alkanes with N-Triftosylhydrazones Leading to Alkyl Aromatics. Chem.

[4] Zhang X., Li L., Sivaguru P., Zanoni G., Bi X. (2022). Highly Electrophilic
Silver Carbenes. Chem. Commun..

[5] Caballero A., Despagnet-Ayoub E., Mar Díaz-Requejo M., Díaz-Rodríguez A., González-Núñez M. E., Mello R., Muñoz B. K., Ojo W.-S., Asensio G., Etienne M., Pérez P. J. (2011). Silver-Catalyzed
C–C Bond Formation between Methane and Ethyl Diazoacetate in
Supercritical CO_2_. Science.

[6] Garrison J. C., Youngs W. J. (2005). Ag­(I) N-Heterocyclic Carbene Complexes:
Synthesis,
Structure, and Application. Chem. Rev..

[7] Hussong M. W., Hoffmeister W. T., Rominger F., Straub B. F. (2015). Copper and Silver
Carbene Complexes without Heteroatom-Stabilization: Structure, Spectroscopy,
and Relativistic Effects. Angew. Chem., Int.
Ed..

[8] Tskhovrebov A. G., Goddard R., Fürstner A. (2018). Two Amphoteric
Silver Carbene Clusters. Angew. Chem., Int.
Ed..

[9] Pei C., Zhang C., Qian Y., Xu X. (2018). Catalytic
Carbene/Alkyne Metathesis (CAM): A Versatile Strategy for Alkyne Bifunctionalization. Org. Biomol. Chem..

[10] Padwa A., Blacklock T. J., Loza R. (1981). Silver-Promoted Isomerizations of Some Cyclopropene Derivatives. J. Am. Chem. Soc..

[11] Hoye T. R., Dinsmore C. J. (1991). Rhodium­(II) Acetate Catalyzed Alkyne Insertion Reactions
of α-Diazo Ketones: Mechanistic Inferences. J. Am. Chem. Soc..

[12] Torres Ò., Parella T., Solà M., Roglans A., Pla-Quintana A. (2015). Enantioselective Rhodium­(I) Donor
Carbenoid-Mediated Cascade Triggered by a Base-Free Decomposition
of Arylsulfonyl Hydrazones. Chem.—Eur.
J..

[13] Marichev K. O., Qiu H., Offield A. C., Arman H., Doyle M. P. (2016). Catalyst-Free Rearrangement of Allenyl Aryldiazoacetates
into 1,5- Dihydro-4H-pyrazol-4-ones. J. Org.
Chem..

[14] Díaz-Jiménez À., Monreal-Corona R., Poater A., Álvarez M., Borrego E., Pérez P. J., Caballero A., Roglans A., Pla-Quintana A. (2022). Intramolecular
Interception of the
Remote Position of Vinylcarbene Silver Complex Intermediates by C­(*sp*
^3^)-H Bond Insertion. Angew. Chem., Int. Ed..

[15] Rosenfeld M. J., Shankar B. K. R., Shechter H. (1988). Rhodium­(II)
acetate-catalyzed reactions of 2-diazo-1,3-indandione and 2-diazo-1-indanone
with various substrates. J. Org. Chem..

[16] Padwa A., Weingarten M. D. (2000). Rhodium­(II)-Catalyzed Carbocyclization Reaction of
α-Diazo Carbonyls with Tethered Unsaturation. J. Org. Chem..

[17] Qiu H., Deng Y., Marichev K. O., Doyle M. P. (2017). Diverse Pathways
in Catalytic Reactions of Propargyl Aryldiazoacetates: Selectivity
Between Three Reaction Sites. J. Org. Chem..

[18] Ma G., Wei K. F., Song M., Dang Y.-L., Yue Y., Han B., Su H., Shen W.-B. (2023). Recent Advances in Transition-Metal-Catalyzed
Büchner Reaction of Alkynes. Org. Biomol.
Chem..

[19] Li X., Sheng H., Song Q. (2023). Rhodium-Catalyzed
Intramolecular Cyclization to Synthesize 2-Aminobenzofurans via Carbene
Metathesis Reactions. Org. Lett..

[20] Kornecki K. P., Briones J. F., Boyarskikh V., Fullilove F., Autschbach J., Schrote K. E., Lancaster K. M., Davies H. M. L., Berry J. F. (2013). Direct Spectroscopic Characterization
of a Transitory Dirhodium Donor-Acceptor Carbene Complex. Science.

[21] IRC analysis of the CH insertion (see Supporting Information) confirms that the reaction proceeds via a concerted and synchronous pathway, which is clearly distinct from a stepwise mechanism with a very low barrier for one step.

[22] Dong K., Fan X., Pei C., Zheng Y., Chang S., Cai J., Qiu L., Yu Z.-X., Xu X. (2020). Transient-Axial-Chirality Controlled
Asymmetric Rhodium-Carbene C­(sp2)-H Functionalization for the Synthesis
of Chiral Fluorenes. Nat. Commun..

[23] Zhu D., Cao T., Chen K., Zhu S. (2022). Rh_2_(II)-Catalyzed Enantioselective Intramolecular Büchner
Reaction and Aromatic Substitution of Donor-Donor Carbenes. Chem. Sci..

[24] Postils V., Rodríguez M., Sabenya G., Conde A., Díaz-Requejo M. M., Pérez P. J., Costas M., Solà M., Luís J. M. (2018). Mechanism
of the Selective Fe-Catalyzed Arene Carbon–Hydrogen
Bond Functionalization. ACS Catal..

[25] For substrate **1m** (*m*-CH_3_), both the formation of **4m** and **4m’** was evaluated, providing the same mechanism type with only slightly different reaction barriers. For the other substituents, only the formation of the major regioisomer, analogous to **4m**, was evaluated.

[26] Kim N., Widenhoefer R. A. (2019). Ionization of Gold (γ-Methoxy)­vinyl Complexes
Generates Reactive Gold Vinyl Carbene Complexes. Chem. Sci..

[27] Nakamura E., Yoshikai N., Yamanaka M. (2002). Mechanism
of C-H Bond Activation/C-C
Bond Formation Reaction Between Diazo Compound and Alkane Catalyzed
by Dirhodium Tetracarboxylate. J. Am. Chem.
Soc..

[28] Lamb K. N., Squitieri R. A., Chintala S. R., Kwong A. J., Balmond E. I., Soldi C., Dmitrenko O., Castiñeira Reis M., Chung R., Addison J. B., Fettinger J. C., Hein J. E., Tantillo D. J., Fox J. M., Shaw J. T. (2017). Synthesis of Benzodihydrofurans by
Asymmetric C–H Insertion Reactions of Donor/Donor Rhodium Carbenes. Chem.—Eur. J..

