# Supplementary material for: Characterization of a Silver Vinylcarbene Intermediate in Carbene–Alkyne Metathesis and Its Concerted C(sp2)–H Bond Insertion
Source: J Am Chem Soc. 2026 Jan 12;148(3):3689–96. doi: 10.1021/jacs.5c19806 (PMC12856905; doi:10.1021/jacs.5c19806)
Supplement: Supplementary file 1 [file ja5c19806_si_001.pdf]

SUPPORTING INFORMATION

Characterization of a Silver Vinylcarbene Intermediate in Carbene–Alkyne Metathesis and Its Concerted C(sp²)–H Bond Insertion

Àlex Díaz-Jiménez,[a,§] Roger Monreal-Corona,[a,§] Arijit Saha,[a] Andrea Álvarez-Núñez,[a] Anna Company,[a] Teodor Parella,[b] Pedro J. Pérez,[c] Ana Caballero,[c] Anna Roglans,[a] Albert Poater,[a,*] Anna Pla-Quintana[a,*]

[a] Institut de Química Computacional i Catàlisi (IQCC) and Departament de Química, Universitat de Girona (UdG), Facultat de Ciències, C/ Maria Aurèlia Capmany, 69, 17003-Girona, Catalunya, Spain.

[b] Servei de Ressonància Magnètica Nuclear, Facultat de Ciències i Biociències, Universitat Autònoma de Barcelona, 08193-Cerdanyola del Vallès, Catalunya, Spain.

[c] Laboratorio de Catálisis Homogénea, Unidad Asociada al CSIC, CIQSO-Centro de Investigación en Química Sostenible and Departamento de Química, Universidad de Huelva, 21007-Huelva, Spain.

[§] These authors contributed equally to this work.

TABLE OF CONTENTS

General materials and methods 3

S1. Synthesized compounds previously reported in the literature 4

S2. General scheme for the synthesis of new diazo compounds 1b,1c,1e and 1m. 4

S3. Experimental procedure for the synthesis of propargyl alcohol S1c........ 4

S4. Experimental procedure for the synthesis of propargyl esters S2b, S2c, S2e and S2m. 5

S5. Experimental procedure for the synthesis of diazo compounds 1b, 1c, 1e and 1m. 7

S6. General procedure for the silver catalyzed carbene/alkyne metathesis tandem reaction 9

S8. Experimental procedure for the synthesis of propargyl alcohol ^{13}C-S1n 16

S9. Experimental procedure for the synthesis of propargyl esters^{13}C-S2h and ^{13}C-S2n. 17

S10. Experimental procedure for the synthesis of diazo compounds ^{13}C-1h and ^{13}C-1n. 18

S11. Spectroscopic characterization of the silver vinylcarbene species 19

S12. Crystal structure of compound 4a 25

S13. Computational details 29

S14. ^{13}C NMR Calculations 30

S15. Bonding analysis 30

S16. Mechanism discussion 31

S17. References 34

S17 NMR Spectra 37

General materials and methods

Unless otherwise noted, materials were obtained from commercial suppliers and used without further purification. Activated molecular sieves (4Å) were added to SPS dried (MBraun, SPS-800) dichloromethane and nitrogen gas was bubbled for 30 minutes to ensure anhydrous and degassed conditions for the reaction solvent. Reaction progress during the preparation of all compounds was monitored using thin layer chromatography on Macherey-Nagel Xtra SIL G/UV254 silica gel plates. Solvents were removed under reduced pressure with a rotary evaporator. Reaction mixtures were chromatographed on silica gel using an automated purification instrument Interchim PuriFlash XS 520 Plus equipped with a quaternary gradient pump (up to 300 ml/min, 20 bar) and an UV-Vis 200-800 nm diode array detector. All ^{1}H and ^{13}C NMR spectra were recorded on a Bruker ASCEND 400 spectrometer equipped with a 5 mm BBFO probe using $CDCl_3$ as a deuterated solvent. ^{1}H and ^{13}C chemical shifts for are reported in ppm (δ) relative to residual solvent signals ($CDCl_3$: 7.26 ppm for ^{1}H, 77,16 ppm for ^{13}C). Coupling constants are given in Hertz (Hz). ^{1}H and ^{13}C NMR signals were assigned based on 2D-NMR: ^{1}H-^{13}C HSQC, ^{1}H-^{13}C HMBC, ^{1}H-^{1}H COSY and ^{1}H-^{1}H TOCSY experiments. Electrospray ionization high-resolution mass spectrometry was performed using a Bruker microTOF-Q II instrument operated in the positive ESI (+) ion mode. IR spectra were recorded on an Agilent Cary 630 FT-IR spectrometer equipped with an ATR sampling accessory. The X-ray intensity data were measured on a 'Bruker D8 QUEST ECO' three-circle diffractometer system equipped with a Ceramic x-ray tube (Mo Kα, λ = 0.71076 Å) and a doubly curved silicon crystal Bruker Triumph monochromator. Melting points were measured in a SMP10 apparatus from Stuart without any correction. UV−vis spectroscopy was performed with an Agilent 50 Scan (Varian) UV−vis spectrophotometer with 1 cm quartz cells. Low temperature control was achieved with a cryostat from Unisoku Scientific Instruments, Japan.

S1. Synthesized compounds previously reported in the literature

Diazo compounds **1a,1d-1i and 1k-1l**[1] and **1j**,[2] were prepared in accordance with the experimental procedure previously described in the literature.

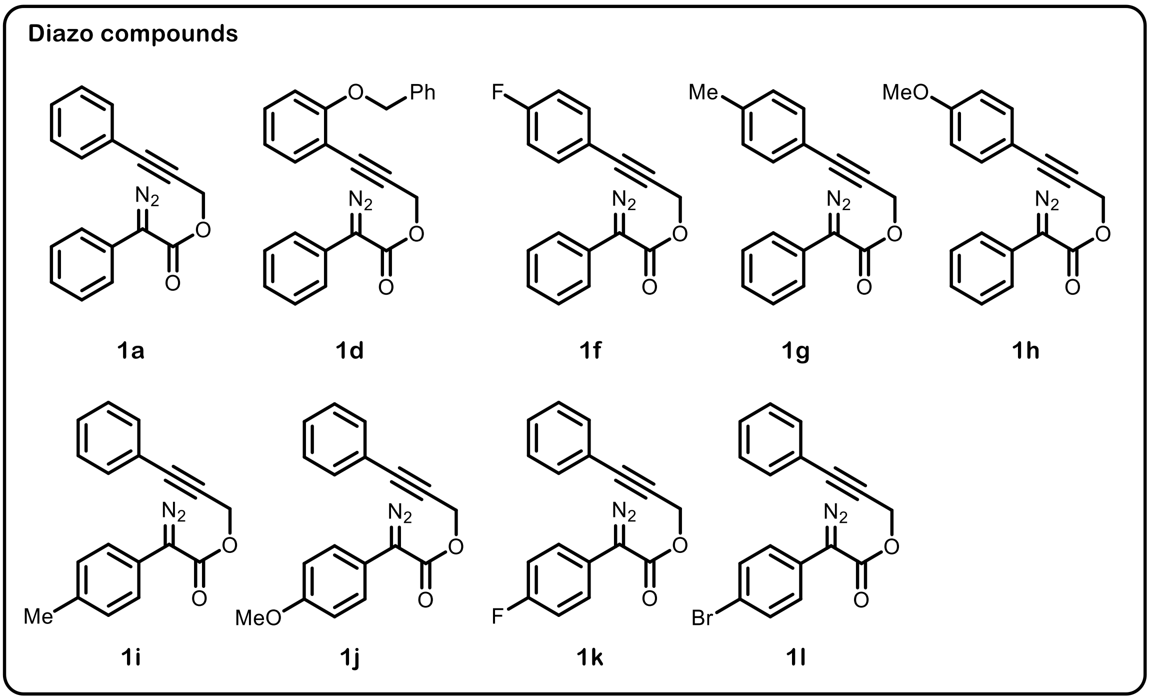

S2. General scheme for the synthesis of new diazo compounds 1b,1c,1e and 1m.

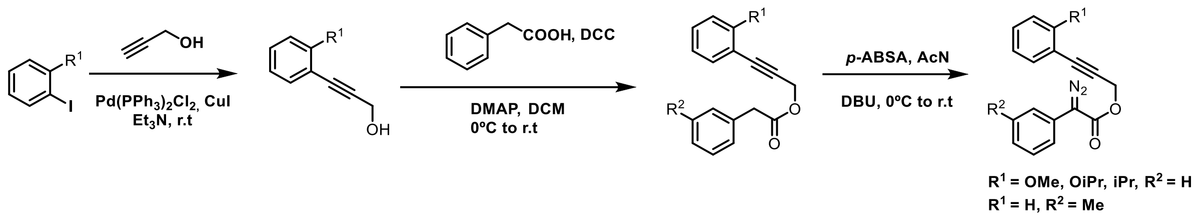

S3. Experimental procedure for the synthesis of propargyl alcohol S1c

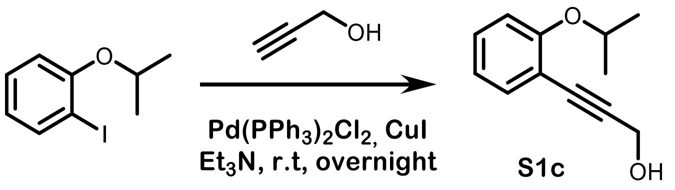

To a 50 mL round-bottom flask containing a mixture of **1-iodo-2-isopropoxybenzene**,[3] (0.98 g, 3.74 mmol), CuI (28.5 mg, 0.15 mmol), and $Pd(PPh_3)_2Cl_2$ (65.6 mg, 0.09 mmol) in triethylamine (25 mL), propargyl alcohol (0.26 mL, 4.47 mmol) was added dropwise under a nitrogen atmosphere. After the addition, the solution was stirred at room temperature overnight. Upon completion of the reaction (TLC monitoring), the crude was filtered through a Celite pad, rinsed

with EtOAc and concentrated under reduced pressure. The crude product was purified by column chromatography on silica gel (Hexanes:EtOAc = 7.5:2.5) to afford **S1c** as a yellow oil (0.42 g, 59 % yield).

MW ($C_{12}H_{14}O_2$): 190.24 g/mol; **Rf:** 0.35 (Hexanes/EtOAc 6:4); **IR (ATR) ν (cm^{-1}):** 3326, 2917, 1485, 1257, 749; **^{1}H NMR ($CDCl_3$, 400.13 MHz):** δ_H 7.39 (dd, 1H, J = 7.7, 1.8 Hz), 7.25 (ddd, 1H, J = 8.4, 7.7, 1.8 Hz), 6.92 – 6.85 (m, 2H), 4.62 – 4.55 (m, 1H), 4.53 (d, 2H, J = 6.0 Hz), 1.75 (t, 1H, J = 6.0 Hz), 1.37 (d, 6H, J = 6.1 Hz). **^{13}C{H} NMR ($CDCl_3$, 100.6 MHz):**δ_C 159.0, 134.0, 129.9, 120.8, 115.0, 113.7, 91.1, 82.6, 72.0, 52.1, 22.3. **HRMS (ESI) m/z:** $[M+Na]^+$ calcd. for $C_{12}H_{14}O_2Na$ 213.0886; Found 213.0886.

S4. Experimental procedure for the synthesis of propargyl esters S2b, S2c, S2e and S2m.

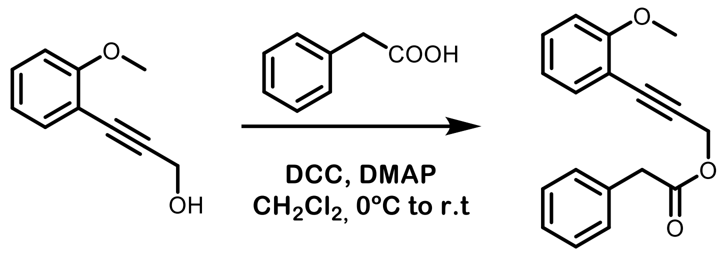

S2b

To a 25 mL round-bottom flask containing a mixture of **3-(2-methoxy-phenyl)-prop-2-yn-1-ol,** [4] (0.42 g, 2.59 mmol), phenylacetic acid (0.39 g, 2.86 mmol), and 4-dimethylaminopyridine (DMAP) (31.8 mg, 0.26 mmol) in dichloromethane (13 mL), N,N'-dicyclohexylcarbodiimide (DCC) (0.65 g, 3.15 mmol) was added in batches at 0 ºC. After the addition, the reaction mixture was slowly warmed to room temperature and stirred overnight. Upon completion of the reaction (TLC monitoring), the crude was filtrated through a Celite pad, rinsed with EtOAc and concentrated under reduced pressure. The crude product was then purified by column chromatography on silica gel (Hexanes/EtOAc = 9:1) to afford ester **S2b** as a yellow oil (0.69 g, 95 % yield).

MW ($C_{18}H_{16}O_3$): 280.32 g/mol; **Rf:** 0.68 (Hexanes/EtOAc 7:3); **IR (ATR) ν (cm^{-1}):** 2932, 1735, 1491, 1262, 1136, 752. **^{1}H NMR ($CDCl_3$, 400.13 MHz):** δ_H 7.42 (dd, 1H, J = 7.6, 1.7 Hz), 7.37 – 7.26 (m, 6H), 6.95 – 6.84 (m, 2H), 4.99 (s, 2H), 3.88 (s, 3H), 3.71 (s, 2H). **^{13}C{H} NMR ($CDCl_3$, 100.6 MHz:** δ_C 171.1, 160.4, 134.2, 133.8, 130.4, 129.5, 128.7, 127.3, 120.6, 111.4, 110.8, 86.9, 83.2, 55.9, 53.7, 41.2. **HRMS (ESI) m/z:** $[M+Na]^+$ calcd. for $C_{18}H_{16}O_3Na$ 303.0992; Found 303.0987.

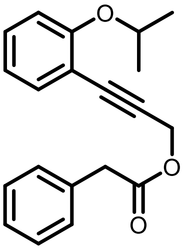

S2c

Ester **S2c** was obtained from **S1c** (0.21 g, 1.10 mmol) as yellow oil (0.29 g, 85%) following the same procedure as for **S2b**.

MW ($C_{20}H_{20}O_3$): 308.38 g/mol; **Rf:** 0.33 (Hexanes/EtOAc 7:3); **IR (ATR) ν (cm^{-1}):** 2974, 1736, 1486, 1261, 1121, 751. **^{1}H NMR ($CDCl_3$, 400.13 MHz):** δ_H 7.40 (dd, 1H, J = 7.8, 1.8 Hz), 7.38 – 7.23 (m, 6H), 6.93 – 6.85 (m, 2H), 4.97 (s, 2H), 4.55 (hept, 1H, J = 6.1 Hz), 3.70 (s, 2H), 1.36 (d, 6H, J = 6.1 Hz). **^{13}C{H} NMR ($CDCl_3$, 100.6 MHz):** δ_C 171.0, 159.2, 134.1, 133.8, 130.1, 129.5, 128.7, 127.3, 120.8, 115.2, 113.4, 86.5, 83.6, 72.1, 53.7, 41.2, 22.3. **HRMS (ESI) m/z:** $[M+Na]^+$ calcd. for $C_{20}H_{20}O_3Na$ 331.1305; Found 331.1310.

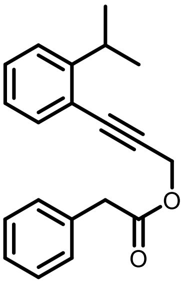

S2e

Ester **S2e** was obtained from **3-(2-Isopropylphenyl)prop-2-yn-1-ol,** [5] (0.41 g, 2.35 mmol) as yellow oil (0.68 g, 99 %) following the same procedure as for **S2b**.

MW ($C_{20}H_{20}O_2$): 292.38 g/mol; **Rf:** 0.63 (Hexanes/EtOAc 8:2); **IR (ATR) ν (cm^{-1}):** 2958, 1737, 1235, 1133, 756. **^{1}H NMR ($CDCl_3$, 400.13 MHz):** δ_H 7.42 (dd, 1H, J = 7.6, 1.4 Hz), 7.38 – 7.27 (m, 7H), 7.13 (td, 1H, J = 7.3, 1.7 Hz), 4.97 (s, 2H), 3.71 (s, 2H), 3.40 (hept, 1H, J = 6.8 Hz), 1.24 (d, 6H, J = 6.8 Hz). **^{13}C{H} NMR ($CDCl_3$, 100.6 MHz):** δ_C 171.0, 151.0, 133.7, 132.8, 129.4, 129.2, 128.7, 127.3, 127.1, 125.6, 125.1, 121.0, 86.5, 85.5, 53.5, 41.2, 31.6, 23.2. **HRMS (ESI) m/z:** $[M+Na]^+$ calcd. $C_{20}H_{20}O_2Na$ 315.1356; Found 315.1362.

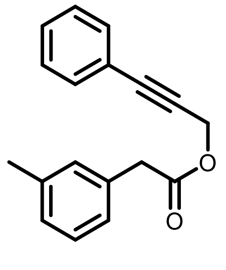

S2m

Ester **S2m** was obtained from **3-Phenylprop-2-yn-1-ol** (0.50 g, 3.78 mmol) as yellow oil (0.95 g, 95 %) following the same procedure as for **S2b**.

MW ($C_{18}H_{16}O_2$): 264.32 g/mol; **Rf:** 0.69 (Hexanes/EtOAc 9:1); **^{1}H NMR ($CDCl_3$, 400.13 MHz):** δ_H 7.49 – 7.40 (m, 2H), 7.35 – 7.29 (m, 3H), 7.25 – 7.18 (m, 1H), 7.16 – 7.05 (m, 3H), 4.94 (s, 2H), 3.67 (s, 2H), 2.35 (s, 3H). **^{13}C{H} NMR ($CDCl_3$, 100.6 MHz):** δ_C 171.2, 138.4, 133.6, 132.0, 130.2, 128.9, 128.7, 128.4, 128.1, 126.4, 122.3, 86.7, 83.0, 53.3, 41.1, 21.5. **HRMS (ESI) m/z:** $[M+Na]^+$ calcd. $C_{18}H_{16}O_2Na$ 287.1047; Found 287.1035.

S5. Experimental procedure for the synthesis of diazo compounds 1b, 1c, 1e and 1m.

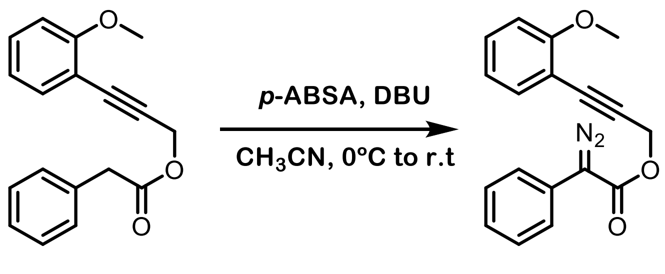

To a 50 mL oven-dried flask containing a mixture of **S2b** (0.40 g, 1.43 mmol) and *p*-acetamidobenzenesulfonyl azide (*p*-ABSA) (0.44 g, 1.83 mmol) in anhydrous CH_3CN (9.3 mL), a solution of 1,8-diazabicyclo[5.4.0]undec-7-ene (DBU) (0.32 mL, 2.14 mmol) in anhydrous CH_3CN (1.9 mL) was added dropwise at 0 ºC. After the addition, the reaction mixture was slowly warmed to room temperature and stirred overnight. Upon completion of the reaction (TLC monitoring), the crude was diluted with dichloromethane, and washed with saturated aqueous NH_4Cl, saturated aqueous $NaHCO_3$ and brine. The combined organic extracts were dried over anhydrous Na_2SO_4 and concentrated under reduced pressure. The crude product was purified by column chromatography on silica gel (Hexanes: Et_3N= 99:1) to afford diazo compound **1b** as a yellow solid (0.29 g, 66% yield).

MW ($C_{18}H_{14}N_2O_3$): 306.32 g/mol; **Rf:** 0.7 (Hexanes/EtOAc 8:2); **IR (ATR) ν (cm^{-1}):** 2935, 2092, 1698, 1235, 1139, 752. **^{1}H NMR ($CDCl_3$, 400.13 MHz):** δ_H 7.54 – 7.47 (m, 2H), 7.44 (dd, 1H, J = 7.6, 1.7 Hz), 7.44 – 7.35 (m, 2H), 7.32 (ddd, 1H, J = 8.3, 7.5, 1.7 Hz), 7.24 – 7.15 (m, 1H), 6.92

(dd, 1H, J = 7.6, 1.0 Hz), 6.88 (d, 1H, J = 9.2 Hz), 5.17 (s, 2H), 3.89 (s, 3H). **^{13}C{H} NMR (CDCl$_3$, 100.6 MHz):** δ_C 164.6, 160.4, 134.2, 130.5, 129.1, 126.1, 125.4, 124.2, 120.6, 111.4, 110.8, 87.0, 83.3, 55.9, 53.5. **HRMS (ESI) m/z:** $[M+Na]^+$ calcd. for $C_{18}H_{14}N_2O_3Na$ 329.0897; Found 329.0895.

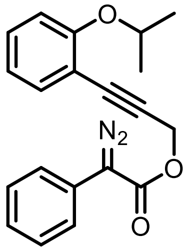

1c

Diazo compound **1c** was obtained from **S2c** (0.29 g, 0.94 mmol) as an orange oil (0.25 g, 80%) following the same procedure as for **1b**.

MW ($C_{20}H_{18}N_2O_3$): 334.38 g/mol; **Rf:** 0.73 (Hexanes/EtOAc 8:2); **IR (ATR) ν (cm^{-1}):** 2974, 2082, 1699, 1486, 1238, 1141, 750. **^{1}H NMR (CDCl$_3$, 400.13 MHz):** δ_H 7.54 – 7.48 (m, 2H), 7.44 – 7.36 (m, 3H), 7.30 – 7.23 (m, 1H (overlapped with chloroform)), 7.23 – 7.16 (m, 1H), 6.93 – 6.86 (m, 2H), 5.15 (s, 2H), 4.56 (hept, 1H, J = 6.1 Hz), 1.37 (d, J = 6.1 Hz, 6H). **^{13}C{H} NMR (CDCl$_3$, 100.6 MHz):** δ_C 164.5, 159.3, 134.0, 130.1, 129.1, 126.0, 125.4, 124.1, 120.8, 115.2, 113.4, 86.6, 83.8, 72.1, 53.6, 22.2. **HRMS (ESI) m/z:** $[M+Na]^+$ calcd. for $C_{20}H_{18}N_2O_3Na$ 357.1210; Found 357.1220.

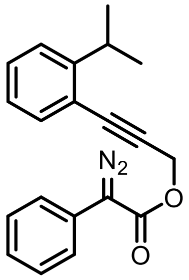

1e

Diazo compound **1e** was obtained from **S2e** (0.18 g, 0.62 mmol) as an orange solid (0.17 g, 86%) following the same procedure as for **1b**.

MW ($C_{20}H_{18}N_2O_2$): 318.38 g/mol; **Rf:** 0.8 (Hexanes/EtOAc 9:1); **IR (ATR) ν (cm^{-1}):** 2960, 2094, 1703, 1237, 1138, 749. **^{1}H NMR (CDCl$_3$, 400.13 MHz):** δ_H 7.52 – 7.47 (m, 2H), 7.45 – 7.38 (m, 3H), 7.33 – 7.28 (m, 2H), 7.20 (ddt, 1H, J = 7.7, 7.0, 1.2 Hz), 7.14 (ddd, 1H, J = 7.7, 7.0, 1.7 Hz), 5.14 (s, 2H), 3.44 (hept, 1H, J = 6.9 Hz), 1.26 (d, 6H, J = 6.9 Hz). **^{13}C{H} NMR (CDCl$_3$, 100.6 MHz):** δ_C 164.6, 151.1, 132.8, 129.3, 129.1, 126.1, 125.6, 125.4, 125.1, 124.2, 121.0, 86.6, 85.8, 53.5, 31.7, 23.2. **HRMS (ESI) m/z:** $[M+H]^+$ calcd. for $C_{20}H_{18}N_2O_2Na$ 341.1260; Found 341.1267.

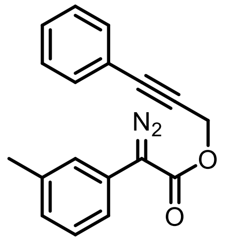

1m

Diazo compound **1m** was obtained from **S2m** (0.55 g, 2.08 mmol) as an orange oil (0.40 g, 66%) following the same procedure as for **1b**.

MW ($C_{18}H_{14}N_2O_2$): 290.32 g/mol; **Rf:** 0.75 (Hexanes/EtOAc 9:1); **^{1}H NMR ($CDCl_3$, 400.13 MHz):** δ_H 7.50 – 7.45 (m, 2H), 7.37 – 7.27 (m, 6H), 7.06 – 6.98 (m, 1H), 5.10 (s, 2H), 2.37 (s, 3H). **^{13}C{H} NMR ($CDCl_3$, 100.6 MHz):** δ_C 164.7, 138.9, 132.1, 129.0, 128.9, 128.5, 127.0, 125.1, 124.8, 122.3, 121.4, 86.8, 83.1, 53.2, 21.7. **HRMS (ESI) m/z:** [M+Na$^+$ calcd. for $C_{18}H_{14}N_2O_2Na$ 313.0947; Found 313.0947.

S6. General procedure for the silver catalyzed carbene/alkyne metathesis tandem reaction

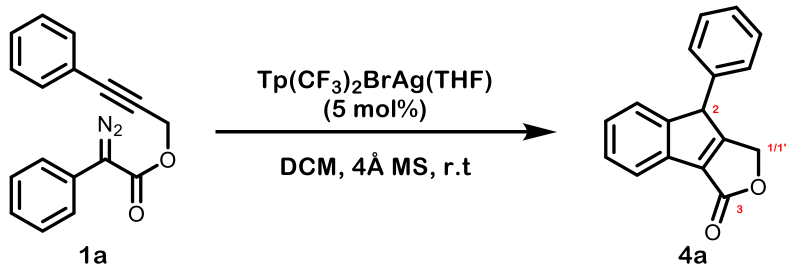

To an oven-dried Schlenk flask containing diazo compound **1a** (19.3 mg, 0.070 mmol) and 4Å MS (100 mg) in anhydrous dichloromethane (14.4 mL), a solution of $Tp(CF_3)_2BrAg(THF)$ (3.6 mg, 0.0035 mmol) in dichloromethane (0.6 mL) was added dropwise under a nitrogen atmosphere. The mixture was stirred in the dark at room temperature for 30 minutes. The solvent was then removed under reduced pressure and the crude reaction mixture was purified by column chromatography on silica gel using hexane/EtOAc mixtures as the eluent (94:6 to 86:14). Concentration under reduced pressure afforded compound **4a** (17.1 mg, 99% yield) as a colorless solid.

MW ($C_{17}H_{12}O_2$): 248.28 g/mol; **Rf:** 0.48 (Hexanes/EtOAc 8:2). **MP (ºC):** 160. **IR (ATR) ν (cm^{-1}):** 3028, 2927,1740, 1012, 748, 695. **^{1}H NMR ($CDCl_3$, 400.13 MHz):** δ_H 7.75 (d, 1H, J = 7.5 Hz), 7.38 (td, 1H, J = 7.2, 1.9 Hz), 7.34 – 7.27 (m, 5H), 7.08 (dd, 2H, J = 7.7, 1.8 Hz), 5.13 (dd, 1H, J = 18.2, 1.1 Hz, **H1/1'**) 4.96 (d, 1H, J = 18.2 Hz, **H1/1'**), 4.91 (s, 1H, **H2**). **^{13}C{H} NMR ($CDCl_3$, 100.6 MHz):** δ_C 175.8(**C3**), 167.7, 151.7, 137.0, 136.2, 134.1, 129.5, 128.1, 128.0, 127.8, 127.3, 125.2,

121.3, 69.2 (**C1**), 53.1(**C2**). **HRMS (ESI) m/z:** $[M+Na]^+$ calcd. for $C_{17}H_{12}O_2Na$ 271.0730; Found 271.0722.

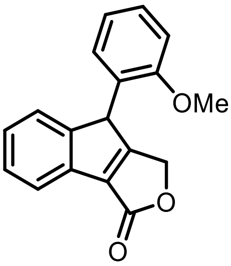

4b

Starting from diazo compound **1b** (30.8 mg, 0.10 mmol), compound **4b** was obtained as a colorless solid (23.0 mg, 82% yield).

MW ($C_{18}H_{14}O_3$): 278.31 g/mol; **Rf:** 0.60 (Hexanes/EtOAc 8:2). **MP (ºC):** 131. **IR (ATR) ν (cm^{-1}):** 3009, 2936,1759, 1486, 1241, 1001, 758. **^{1}H NMR ($CDCl_3$, 400.13 MHz):** δ_H 7.76 (d, 1H, J = 7.4 Hz), 7.45 – 7.36 (m, 2H), 7.36 – 7.27 (m, 2H), 6.98 (d, 1H, J = 8.2 Hz), 6.89 – 6.78 (m, 2H), 5.34 (s, 1H), 5.16 (dd, 1H, J = 18.3, 1.2 Hz), 4.89 (d, 1H, J = 18.3 Hz), 3.94 (s, 3H).**^{13}C{H} NMR ($CDCl_3$, 100.6 MHz):** δ_C 176.5, 168.0, 157.5, 149.8, 135.8, 135.0, 129.1, 127.8, 127.7, 126.8, 125.7, 124.3, 121.4, 121.3, 110.7, 69.9, 55.7, 47.4.**HRMS (ESI) m/z:** $[M+Na]^+$ calcd. for $C_{18}H_{14}O_3Na$ 301.0835; Found 301.0831.

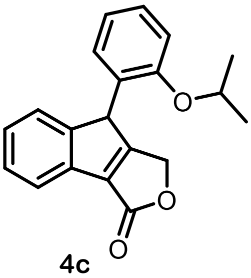

4c

Starting from diazo compound **1c** (23.2 mg, 0.069 mmol), compound **4c** was obtained as a colorless solid (21.0 mg, 99% yield).

MW ($C_{20}H_{18}O_3$): 306.36 g/mol; **Rf:** 0.48 (Hexanes/EtOAc 8:2). **MP (ºC):** 165. **IR (ATR) ν (cm^{-1}):** 2983, 1757, 1449, 1240, 1110, 753. **^{1}H NMR ($DMSO_{d6}$, 400.13 MHz, 65ºC):** δ_H 7.58 (d, 1H, J = 7.3 Hz), 7.38 (td, 1H, J = 7.3, 1.5 Hz), 7.35 – 7.22 (m, 3H), 7.04 (d, 2H, J = 8.2 Hz), 6.85 (td, 1H, J = 7.4, 1.1 Hz), 5.30 (s, 1H), 5.28 (d, 2H, J = 17.5 Hz), 4.98 (d, 1H, J = 17.5 Hz), 4.63 (hept, 1H, J = 6.0 Hz), 1.24 (d, 3H, J = 6.0 Hz), 1.05 (d, 3H, J = 6.0 Hz).**^{13}C{H} NMR ($DMSO_{d6}$, 100.6 MHz, 65ºC):** δ_C 177.9, 166.9, 155.0, 150.7, 134.0, 133.9, 128.5, 126.9, 126.1, 124.7, 124.6, 120.1, 119.5, 112.9, 69.5, 69.2, 21.4, 21.0. **HRMS (ESI) m/z:** $[M+Na]^+$ calcd. for $C_{20}H_{18}O_3Na$ 329.1148; Found 329.1152.

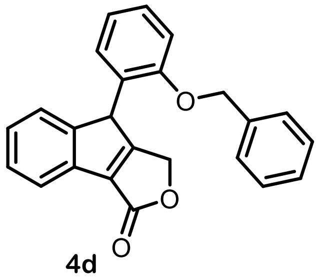

4d

Starting from diazo compound **1d** (27.1 mg, 0.071 mmol), compound **4d** was obtained as a colorless solid (24.4 mg, 97% yield).

MW ($C_{24}H_{18}O_3$): 354.41 g/mol; **Rf:** 0.40 (Hexanes/EtOAc 8:2). **MP (ºC):** 139. **IR (ATR) ν (cm^{-1}):** 3033, 2915, 1756, 1449, 1241, 1008, 750. **^1H NMR ($CDCl_3$, 400.13 MHz):** δ_H 7.73 (dd, 1H, J = 7.5, 1.3 Hz), 7.45 – 7.35 (m, 7H), 7.34 – 7.27 (m, 2H), 7.07 (d, 1H, J = 8.2 Hz), 6.91 – 6.86 (m, 2H), 5.35 (s, 1H), 5.16 (d, 1H, J = 11.2 Hz), 5.12 (d, 1H, J = 11.2 Hz), 4.96 (dd, 1H, J = 18.4, 1.1 Hz), 4.85 (d, 1H, J = 18.4 Hz). **$^{13}C\{H\}$ NMR ($CDCl_3$, 100.6 MHz):** δ_C 176.3, 167.8, 156.7, 149.8, 136.3, 135.8, 135.0, 129.2, 129.0, 128.6, 127.8, 126.8, 125.6, 124.6, 121.6, 121.3, 111.9, 70.6, 69.9, 47.8. **HRMS (ESI) m/z:** $[M+Na]^+$ calcd. for $C_{24}H_{18}O_3Na$ 377.1148; Found 377.1143.

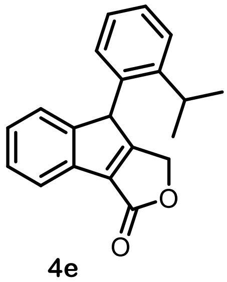

4e

Starting from diazo compound **1e** (23.5 mg, 0.074 mmol), compound **4e** was obtained as a colorless solid (17.3 mg, 81% yield).

MW ($C_{20}H_{18}O_2$): 290.36 g/mol; **Rf:** 0.53 (Hexanes/EtOAc 8:2). **MP (ºC):** 145. **IR (ATR) ν (cm^{-1}):** 2960, 1753, 1361, 1215, 764. **^1H NMR ($CDCl_3$, 400.13 MHz):** δ_H 7.77 (d, 1H, J = 7.6 Hz), 7.42 – 7.36 (m, 2H), 7.28 – 7.23 (m, 3H, (overlapped with chloroform)), 7.00 (td, 1H, J = 7.6, 1.4 Hz), 6.47 (dd, 1H, J = 7.8, 1.4 Hz), 5.31 (s, 1H), 5.10 (d, 1H, J = 18.1 Hz), 4.92 (d, 1H, J = 18.1 Hz), 3.50 (hept, 1H, J = 6.8 Hz), 1.48 (d, 3H, J = 6.8 Hz), 1.37 (d, 3H, J = 6.8 Hz). Some small signals appear and some multiplets are wider due to restricted rotation alongside de C^{sp3}-C^{sp2} axis. **$^{13}C\{H\}$ NMR ($CDCl_3$, 100.6 MHz):** δ_C 176.7, 167.7, 152.4, 146.8, 136.9, 134.4, 133.0, 128.3, 127.8, 127.2, 127.0, 126.8, 126.2, 125.1, 121.4, 69.1, 48.5, 29.8, 24.4, 24.2. **HRMS (ESI) m/z:** $[M+Na]^+$ calcd. for **$C_{20}H_{18}O_2$**Na 313.1199; Found 313.1202.

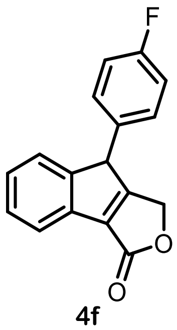

4f

Starting from diazo compound **1f** (30.0 mg, 0.10 mmol), compound **4f** was obtained as a colorless solid (22.0 mg, 81% yield).

MW ($C_{17}H_{11}FO_2$): 266.27 g/mol; **Rf:** 0.48 (Hexanes/EtOAc 8:2). **MP (ºC):** 161. **IR (ATR) ν (cm^{-1}):** 3036, 2903,1741, 1501, 1217, 1010, 776. **^{1}H NMR ($CDCl_3$, 400.13 MHz):** δ_H 7.75 (d, 1H, J = 7.5 Hz), 7.39 (tt, 1H, J = 7.5, 4.9 Hz), 7.31 – 7.27 (m, 2H), 7.10 – 6.97 (m, 4H), 5.13 (dd, 1H, J = 18.3, 1.1 Hz), 4.94 (d, 1H, J = 18.3 Hz), 4.88 (s, 1H).**^{13}C{H} NMR ($CDCl_3$, 100.6 MHz):** δ_C 175.5, 167.5, 162.5 (d, J = 247.2 Hz), 151.6, 137.1, 134.0, 131.9 (d, J = 3.4 Hz), 129.5 (d, J = 8.2 Hz), 128.1, 127.4, 125.2, 121.3, 116.5 (d, J = 21.7 Hz), 69.1, 52.3. **^{19}F NMR ($CDCl_3$, 376 MHz):** δ_F -114.96. (s, 1F). **HRMS (ESI) m/z:** [M+Na]$^+$ calcd. for $C_{17}H_{11}FO_2Na$ 289.0635; Found 289.0634.

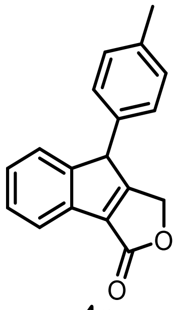

4g

Starting from diazo compound **1g** (22.2 mg, 0.076 mmol), compound **4g** was obtained as a colorless solid (20.0 mg, 99% yield).

MW ($C_{18}H_{14}O_2$): 262.31 g/mol; **Rf:** 0.45 (Hexanes/EtOAc 8:2). **MP (ºC):** 175. **IR (ATR) ν (cm^{-1}):** 3014, 2922,1744, 1005, 774, 723. **^{1}H NMR ($CDCl_3$, 400.13 MHz):** δ_H 7.74 (d, 1H, J = 7.6 Hz), 7.37 (td, 1H, J = 7.2, 1.9 Hz), 7.31 – 7.26 (m, 2H), 7.13 (d, 2H, J = 7.8 Hz), 6.96 (d, 2H, J = 8.1 Hz), 5.12 (dd, 1H, J = 18.3, 1.1 Hz), 4.95 (d, 1H, J = 18.3 Hz), 4.87 (s, 1H), 2.33 (s, 3H).**^{13}C{H} NMR ($CDCl_3$, 100.6 MHz):** δ_C 176.1, 167.7, 151.9, 137.9, 136.8, 134.1, 133.1, 130.2, 127.9, 127.7, 127.2, 125.2, 121.2, 69.2, 52.8, 21.2. **HRMS (ESI) m/z:** [M+Na]$^+$ calcd. for $C_{18}H_{14}O_2Na$ 284.0803; Found 284.0808.

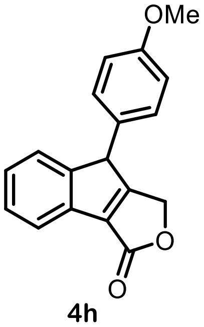

4h

Starting from diazo compound **1h** (20.1 mg, 0.066 mmol), compound **4h** was obtained as a colorless solid (16.0 mg, 88% yield).

MW ($C_{18}H_{14}O_3$): 278.31 g/mol; **Rf:** 0.23 (Hexanes/EtOAc 8:2). **MP (ºC):** 178. **IR (ATR) ν (cm^{-1}):** 3009, 2918,1742, 1506, 1252, 1006, 774. **^{1}H NMR ($CDCl_3$, 400.13 MHz):** δ_H 7.74 (d, 1H, J = 7.5 Hz), 7.37 (ddd, 1H, J = 7.5, 6.1, 2.1 Hz), 7.30 – 7.26 (m, 2H), 7.02 – 6.95 (m, 2H), 6.89 – 6.81 (m, 2H), 5.12 (dd, 1H, J = 18.3, 1.1 Hz), 4.95 (d, 1H, J = 18.3 Hz), 4.86 (s, 1H), 3.79 (s, 3H). **^{13}C{H} NMR ($CDCl_3$, 100.6 MHz):** δ_C 176.2, 167.7, 159.4, 152.0, 136.7, 134.0, 128.9, 127.9, 127.8, 127.2, 125.2, 121.2, 114.9, 69.2, 55.5, 52.4. **HRMS (ESI) m/z:** $[M+Na]^+$ calcd. for $C_{18}H_{14}O_3Na$ 301.0835; Found 301.0835.

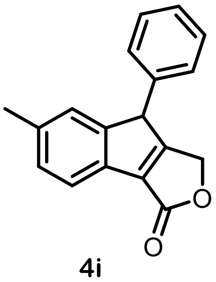

4i

Starting from diazo compound **1i** (21.0 mg, 0.072 mmol), compound **4i** was obtained as a colorless solid (18.6 mg, 98% yield).

MW ($C_{18}H_{14}O_2$): 262.31 g/mol; **Rf:** 0.45 (Hexanes/EtOAc 8:2). **MP (ºC):** 175. **IR (ATR) ν (cm^{-1}):** 3028, 2914,1742, 1013, 824, 695. **^{1}H NMR ($CDCl_3$, 400.13 MHz):** δ_H 7.62 (d, 1H, J = 7.7 Hz), 7.37 – 7.27 (m, 3H), 7.18 (d, 1H, J = 8.5 Hz), 7.13 – 7.05 (m, 3H), 5.11 (dd, 1H, J = 18.1, 1.1 Hz), 4.94 (d, 1H, J = 18.1 Hz), 4.85 (s, 1H), 2.35 (s, 3H). **^{13}C{H} NMR ($CDCl_3$, 100.6 MHz):** δ_C 174.9, 167.8, 152.0, 137.3, 136.9, 136.6, 131.4, 129.5, 128.6, 128.0, 127.9, 126.0, 120.9, 69.2, 53.0, 21.7. **HRMS (ESI) m/z:** $[M+Na]^+$ calcd. for $C_{18}H_{14}O_2Na$ 285.0886; Found 285.0892.

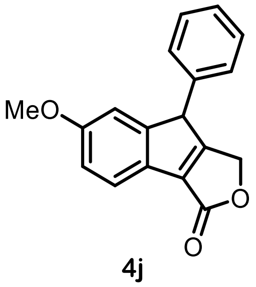

4j

Starting from diazo compound **1j** (21.5 mg, 0.070 mmol), compound **4j** was obtained as a colorless solid (19.2 mg, 98% yield).

MW ($C_{18}H_{14}O_3$): 278.31 g/mol; **Rf:** 0.30 (Hexanes/EtOAc 8:2). **MP (ºC):** 163. **IR (ATR) ν (cm^{-1}):** 2931, 1735, 1478, 1269, 1013, 769. **^1H NMR ($CDCl_3$, 400.13 MHz):** δ_H 7.64 (d, 1H, J = 8.3 Hz), 7.38 – 7.28 (m, 3H), 7.12 – 7.05 (m, 2H), 6.90 (dd, 1H, J = 8.3, 2.4 Hz), 6.86 (d, 1H, J = 2.4 Hz), 5.09 (dd, 1H, J = 18.2, 1.2 Hz), 4.92 (d, 1H, J = 18.2 Hz), 4.85 (s, 1H), 3.78 (s, 3H). **$^{13}C\{H\}$ NMR ($CDCl_3$, 100.6 MHz):** δ_C 173.6, 167.8, 159.6, 153.7, 136.6, 136.5, 129.5, 128.1, 127.9, 126.9, 121.7, 112.8, 112.3, 69.2, 55.7, 53.1. **HRMS (ESI) m/z:** $[M+Na]^+$ calcd. for $C_{18}H_{14}O_3Na$ 301.0835; Found 301.0833.

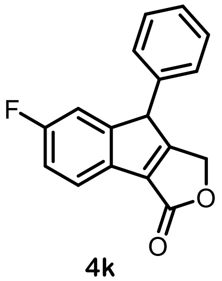

4k

Starting from diazo compound **1k** (20.5 mg, 0.070 mmol), compound **4k** was obtained as a colorless solid (16.4 mg, 88% yield).

MW ($C_{17}H_{11}FO_2$): 266.27 g/mol; **Rf:** 0.40 (Hexanes/EtOAc 8:2). **MP (ºC):** 178. **IR (ATR) ν (cm^{-1}):** 2918, 1740, 1471, 1013, 834, 697. **^1H NMR ($CDCl_3$, 400.13 MHz):** δ_H 7.68 (dd, 1H, J = 8.6, 5.1 Hz), 7.40 – 7.28 (m, 3H), 7.13 – 7.04 (m, 3H), 7.01 (dd, 1H, J = 8.6, 2.3 Hz), 5.12 (d, 1H, J = 18.3 Hz), 4.95 (d, 1H, J = 18.3 Hz), 4.89 (s, 1H). **$^{13}C\{H\}$ NMR ($CDCl_3$, 100.6 MHz):** δ_C 175.2 (d, J = 3.5 Hz), 167.4, 162.5 (d, J = 247.1 Hz), 154.0 (d, J = 8.3 Hz), 136.3, 135.6, 130.1 (d, J = 2.6 Hz), 129.7, 128.4, 127.8, 122.1 (d, J = 8.9 Hz), 115.0 (d, J = 23.1 Hz), 113.3 (d, J = 23.9 Hz), 69.1, 53.2, 53.1. **^{19}F NMR ($CDCl_3$, 376 MHz):** δ_F -114.68. (s, 1F). **HRMS (ESI) m/z:** $[M+Na]^+$ calcd. for $C_{17}H_{11}FO_2Na$ 289.0635; Found 289.0643.

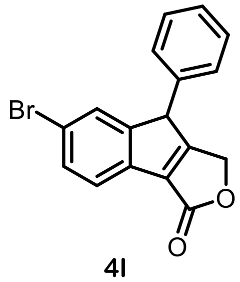

4l

Starting from diazo compound **1l** (24.6 mg, 0.070 mmol), compound **4l** was obtained as a colorless solid (20.3 mg, 89% yield).

MW ($C_{17}H_{11}BrO_2$): 327.18 g/mol; **Rf:** 0.30 (Hexanes/EtOAc 8:2). **MP (ºC):** 163. **IR (ATR) *ν* (cm^{-1}):** 2918, 1745, 1451, 1012, 948, 827, 698. **^{1}H NMR ($CDCl_3$, 400.13 MHz):** δ_H 7.61 (d, 1H, J = 8.0 Hz), 7.53 (dd, 1H, J = 8.0, 1.8 Hz), 7.42 (broad signal, 1H), 7.40 – 7.28 (m, 3H), 7.07 (dd, 2H, J = 7.5, 2.1 Hz), 5.11 (dd, 1H, J = 18.4, 1.1 Hz), 4.95 (d, 1H, J = 18.4 Hz), 4.89 (s, 1H). **^{13}C{H} NMR ($CDCl_3$, 100.6 MHz):** δ_C 175.8, 167.2, 153.6, 136.5, 135.3, 133.0, 131.2, 129.7, 128.7, 128.4, 127.8, 122.4, 121.5, 69.1, 53.1. **HRMS (ESI) m/z:** $[M+Na]^+$ calcd. for $C_{17}H_{11}BrO_2Na$ 348.9835-350.9815; Found 348.9834-350.9816.

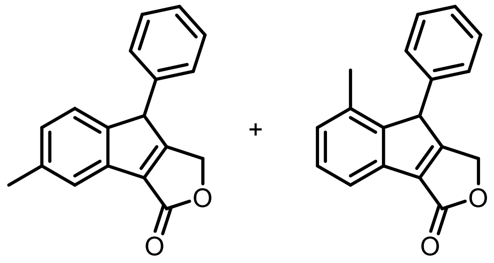

4m **4m'**

Starting from diazo compound **1m** (31.0 mg, 0.11 mmol), compound **4m** (13.9 mg, 50% yield) and compound **4m'** (9.6 mg, 34% yield) were obtained as colorless solids.

4m: MW ($C_{18}H_{14}O_2$): 262.31 g/mol; **Rf:** 0.33 (Hexanes/EtOAc 8:2). **MP (ºC):** 148. **^{1}H NMR ($CDCl_3$, 400.13 MHz):** δ_H 7.58 (s, 1H), 7.36 – 7.27 (m, 3H), 7.17 (d, *J* = 7.8 Hz, 1H), 7.10 – 7.04 (m, 3H), 5.12 (d, *J* = 18.4 Hz, 1H), 4.94 (d, *J* = 18.4 Hz, 1H), 4.87 (s, 1H), 2.41 (s, 3H). **^{13}C{H} NMR ($CDCl_3$, 100.6 MHz):** δ_C 176.1, 167.8, 148.8, 137.9, 136.8, 136.6, 134.2, 129.5, 128.0, 127.9, 127.8, 124.9, 122.0, 69.3, 52.8, 21.5. **HRMS (ESI) m/z:** $[M+H]^+$ calcd. for $C_{18}H_{15}O_2$ 263.1067; Found 263.1060.

4m': MW ($C_{18}H_{14}O_2$): 262.31 g/mol; **Rf:** 0.31 (Hexanes/EtOAc 8:2). **MP (ºC):** 185. **^{1}H NMR ($CDCl_3$, 400.13 MHz):** δ_H 7.61 (d, *J* = 7.6 Hz, 1H), 7.37 – 7.27 (m, 4H), 7.07 (d, *J* = 7.6 Hz, 1H), 7.04 – 6.99 (m, 2H), 5.10 (dd, *J* = 18.2, 1.1 Hz, 1H), 4.88 (s, 1H), 4.78 (d, *J* = 18.2 Hz, 1H), 2.05 (s, 3H). **^{13}C{H} NMR ($CDCl_3$, 100.6 MHz):** δ_C 176.4, 167.9, 149.3, 136.0, 135.3, 134.6, 129.5, 129.0, 128.4, 128.0, 127.8, 118.9, 69.0, 52.7, 18.8. **HRMS (ESI) m/z:** $[M+H]^+$ calcd. for $C_{18}H_{15}O_2$ 263.1067; Found 263.1064.

S7. General scheme for the synthesis of new diazo compounds ^{13}C-1h and ^{13}C-1n.

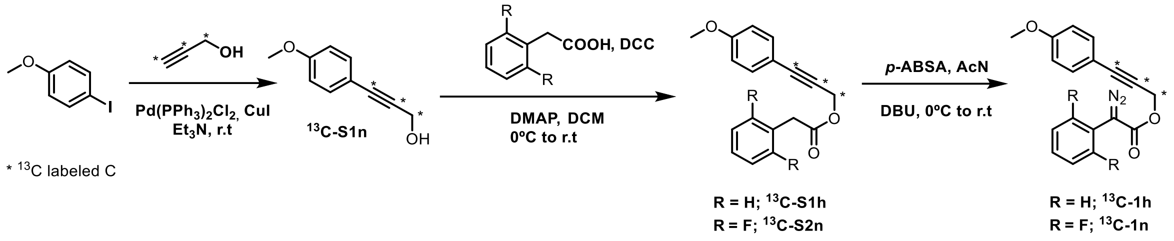

S8. Experimental procedure for the synthesis of propargyl alcohol ^{13}C-S1n

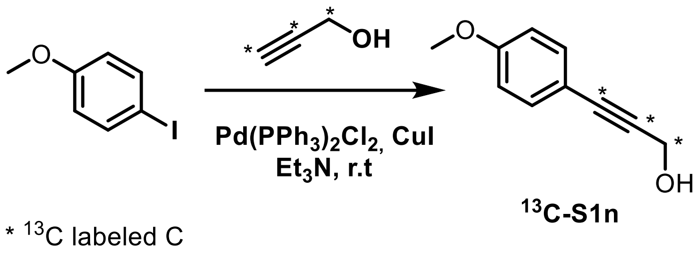

To a 50 mL round-bottom flask containing a mixture of **4-iodoanisole** (0.47 g, 2.0 mmol), CuI (11.4 mg, 0.06 mmol), and $Pd(PPh_3)_2Cl_2$ (21.0 mg, 0.03 mmol) in triethylamine (10 mL), propargyl alcohol (^{13}C labeled) (0.092 mL, 1.50 mmol) was added dropwise under a nitrogen atmosphere. After the addition, the solution was stirred at room temperature overnight. Upon completion of the reaction (TLC monitoring), the crude was filtered through a Celite pad, rinsed with EtOAc and concentrated under reduced pressure. The crude product was purified by column chromatography on silica gel (Hexanes:EtOAc = 7.5:2.5) to afford **^{13}C-S1n** as a yellow solid (0.222 g, 96 % yield).

MW ($C_7{}^{13}C_3H_{10}O_2$): 165.16 g/mol; **Rf:** 0.22 (Hexanes/EtOAc 8:2); **^{1}H NMR ($CDCl_3$, 400.13 MHz):** δ_H 7.39 -7.35 (m, 2H), 6.84 (d, J = 8.0 Hz, 2H), 4.47 (d, $^1J_{13C\text{-}1H}$= 148.0 Hz, 2H), 3.81 (s, 3H).**^{13}C{H} NMR ($CDCl_3$, 100.6 MHz):** δ_C 162.4, 159.9, 133.3 (t, J = 3 Hz), 114.1 (*t*, J = 3 Hz), 85.9 (d, $^1J_{13C\text{-}13C}$ = 46 Hz), 85.8 (d, $^2J_{13C\text{-}13C}$ = 41 Hz), 55.4, 51.8 (dd, $^1J_{13C\text{-}13C}$ = 46 Hz, $^2J_{13C\text{-}13C}$ = 41 Hz).). **HRMS (ESI) m/z:** $[M+Na]^+$ calcd. for $C_7{}^{13}C_3H_{10}O_2Na$ 188.0674; Found 188.0679.

S9. Experimental procedure for the synthesis of propargyl esters ^{13}C-S2h and ^{13}C-S2n.

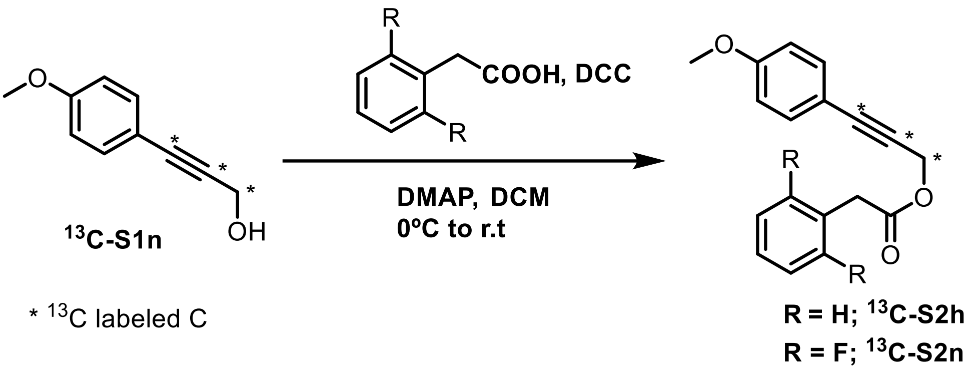

To a 25 mL round-bottom flask containing a mixture of **^{13}C-S1n** (0.122 g, 0.74 mmol), phenylacetic acid (0.151 g, 1.11 mmol), and 4-dimethylaminopyridine (DMAP) (18 mg, 0.14 mmol) in dichloromethane (15 mL), N,N'-dicyclohexylcarbodiimide (DCC) (0.244 g, 1.11 mmol) was added in batches at 0 ºC. After the addition, the reaction mixture was slowly warmed to room temperature and stirred overnight. Upon completion of the reaction (TLC monitoring), the crude was filtrated through a Celite pad, rinsed with EtOAc and concentrated under reduced pressure. The crude product was then purified by column chromatography on silica gel (Hexanes/EtOAc = 9:1) to afford ester **^{13}C-S2h** as a colorless oil (0.193 g, 92% yield).

MW ($C_{15}{}^{13}C_3H_{16}O_3$): 283.30 g/mol; **Rf:** 0.55 (Hexanes/EtOAc 9:1). **^{1}H NMR ($CDCl_3$, 400.13 MHz):** δ_H 7.38 (dd, J = 8.8, 5.0 Hz, 2H), 7.34 – 7.28 (m, 5H), 6.85 (d, J = 8.0 Hz, 2H), 4.92 (ddd, $^1J_{13C\text{-}1H}$ =152, $^2J_{13C\text{-}1H}$ = 8, $^3J_{13C\text{-}1H}$ = 4 Hz, 2H), 3.81 (s, 3H), 3.70 (s, 2H). **^{13}C{H} NMR ($CDCl_3$, 100.6 MHz):** δ_C 171.1, 160.1, 133.7, 133.6 (m), 129.4, 128.8, 127.4, 114.1 (d, $^3J_{13C\text{-}13C}$ = 5 Hz), 86.9 (dd, $^1J_{13C\text{-}13C}$ = 183 Hz, $^2J_{13C\text{-}13C}$ = 16 Hz), 81.4 (dd, $^1J_{13C\text{-}13C}$ = 79 Hz, $^1J_{13C\text{-}13C}$ = 183 Hz), 55.4, 53.5 (dd, $^1J_{13C\text{-}13C}$ = 79 Hz, $^2J_{13C\text{-}13C}$ = 16 Hz), 41.2. **HRMS (ESI) m/z:** $[M+Na]^+$ calcd. for $C_{15}{}^{13}C_3H_{16}O_3Na$ 306.1092; Found 306.1091.

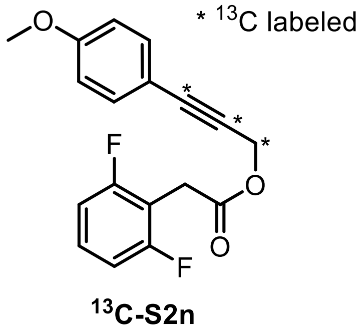

^{13}C-S2n

Ester **^{13}C-S2n** was obtained from **^{13}C-S1n** (122 mg, 0.74 mmol) as a colorless oil (197.0 mg, 84% yield) following the same procedure as for **^{13}C-S2h**.

MW ($C_{15}{}^{13}C_3H_{14}F_2O_3$): 319.28 g/mol; **Rf:** 0.60 (Hexanes/EtOAc 9:1). **^{1}H NMR ($CDCl_3$, 400.13 MHz):** δ_H 7.42 – 7.37 (m, 2H), 7.29 – 7.21 (m, 1H), 6.91 (dd, J = 8.4, 7.2 Hz, 2H), 6.84 (d, J = 8.8 Hz, 2H), 4.95 (ddd, $^1J_{13C\text{-}1H}$ = 152.9 Hz, $^2J_{13C\text{-}1H}$ = 7.7 Hz, $^3J_{13C\text{-}1H}$ = 3.8 Hz, 2H), 3.81 (s, 3H), 3.79 (s, 2H). **^{13}C{H} NMR ($CDCl_3$, 100.6 MHz):** δ_C 169.3, 161.6 (dd, $^1J_{13C\text{-}19F}$ = 247 Hz, $^3J_{13C\text{-}19F}$ = 7 Hz), 160.1, 133.6, 129.3 (t, $^3J_{13C\text{-}19F}$ = 10 Hz), 114.1 (d, $^3J_{13C\text{-}13C}$ = 5.9 Hz), 111.29 (dd, $^2J_{13C\text{-}19F}$ = 25.0 Hz, $^4J_{13C\text{-}19F}$ = 6 Hz), 110.3 (t, $^2J_{13C\text{-}19F}$ = 20 Hz), 87.0 (dd, $^1J_{13C\text{-}13C}$ = 183 Hz, $^2J_{13C\text{-}13C}$ = 16 Hz), 81.2 (dd, $^1J_{13C\text{-}13C}$ = 79 Hz, $^1J_{13C\text{-}13C}$ = 183 Hz), 55.4, 53.9 (dd, $^1J_{13C\text{-}13C}$ = 79 Hz, $^2J_{13C\text{-}13C}$ = 16 Hz), 28.0. **^{19}F NMR ($CDCl_3$, 376 MHz,):** δ_F -115.3. **HRMS (ESI) m/z:** $[M+Na]^+$ calcd. for $C_{15}{}^{13}C_3H_{14}F_2O_3Na$ 342.0904; Found 342.0903.

S10. Experimental procedure for the synthesis of diazo compounds ^{13}C-1h and ^{13}C-1n.

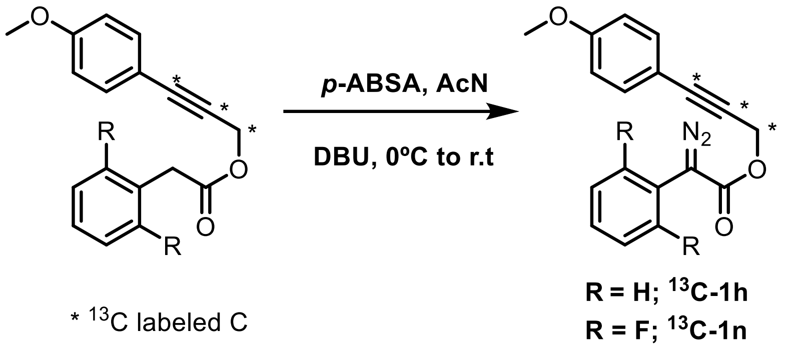

R = H; ^{13}C-1h
R = F; ^{13}C-1n

To a 50 mL oven-dried flask containing a mixture of **^{13}C-S2h** (0.11 mg, 0.4 mmol) and *p*-acetamidobenzenesulfonyl azide (*p*-ABSA) (0.145 g, 0.6 mmol) in anhydrous CH_3CN (10 mL), a solution of 1,8-diazabicyclo[5.4.0]undec-7-ene (DBU) (0.12 mL, 0.8 mmol) in anhydrous CH_3CN (2 mL) was added dropwise at 0 °C. After the addition, the reaction mixture was slowly warmed to room temperature and stirred overnight. Upon completion of the reaction (TLC monitoring), the crude was diluted with dichloromethane, and washed with saturated aqueous NH_4Cl, saturated aqueous $NaHCO_3$ and brine. The combined organic extracts were dried over anhydrous Na_2SO_4 and concentrated under reduced pressure. The crude product was purified by column chromatography on silica gel (Hexanes/EtOAc = 9:1) to afford diazo compound **^{13}C-1h** as a yellow solid (112 mg, 91% yield).

MW ($C_{15}{}^{13}C_3H_{14}N_2O_3$): 309.29 g/mol; **Rf:** 0.62 (Hexanes/EtOAc 9:1). **^{1}H NMR ($CDCl_3$, 400.13 MHz):** δ_H 7.50 (dd, J = 8.7, 1.3 Hz, 2H), 7.43 – 7.38 (m, 4H), 7.20 (tt, J = 7.4, 1.2 Hz, 1H), 6.84 (d, J = 8.0 Hz, 2H), 5.09 (ddd, $^1J_{13C\text{-}1H}$ = 152Hz, $^2J_{13C\text{-}1H}$ = 8 Hz, $^3J_{13C\text{-}1H}$ = 4 Hz, 2H), 3.81 (s, 3H). **^{13}C{H} NMR ($CDCl_3$, 100.6 MHz):** δ_C 164.6, 160.2, 133.6 (m), 129.1, 126.1, 125.4, 124.2, 114.1 (d, $^3J_{13C\text{-}13C}$ = 6 Hz), 87.0 (dd, $^1J_{13C\text{-}13C}$ = 184 Hz, $^2J_{13C\text{-}13C}$ = 16 Hz), 81.6 (dd, $^1J_{13C\text{-}13C}$ = 80 Hz, $^1J_{13C\text{-}13C}$ = 184 Hz), 55.4, 53.4 (dd, $^1J_{13C\text{-}13C}$ = 79 Hz, $^2J_{13C\text{-}13C}$ = 16 Hz). **HRMS (ESI) m/z:** $[M+Na]^+$ calcd. for **$C_{15}{}^{13}C_3H_{14}N_2O_3$**Na 332.0992; Found 332.1002.

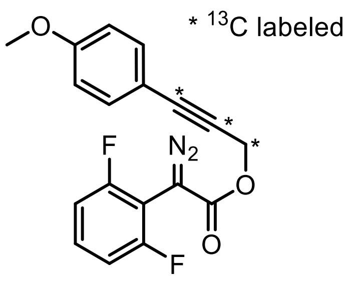

^{13}C-1n

Diazo compound **^{13}C-1n** was obtained from **^{13}C-S2n** (110 mg, 0.35 mmol) as an yellow oil (116 mg, 96% yield) following the same procedure as for **^{13}C-1h**.

MW ($C_{15}{}^{13}C_3H_{12}F_2N_2O_3$): 345.27 g/mol; **Rf:** 0.68 (Hexanes/EtOAc 9:1). **^{1}H NMR ($CDCl_3$, 400.13 MHz):** δ_H 7.42 - 7.39 (m, 2H), 7.37 – 7.31 (m, 1H), 6.98 (t, J = 7.7 Hz, 2H), 6.84 (d, J = 8.8 Hz, 2H), 5.06 (ddd, $^1J_{13C\text{-}1H}$ = 153 Hz, $^2J_{13C\text{-}1H}$ = 7.6 Hz, $^3J_{13C\text{-}1H}$ = 3.7 Hz, 2H), 3.81 (s, 3H). **^{13}C{H} NMR ($CDCl_3$, 100.6 MHz):** δ_C 221.2, 163.9, 160.7 (dd, $^1J_{13C\text{-}19F}$ = 252.9 Hz, $^3J_{13C\text{-}19F}$ = 5.5 Hz), 160.1, 133.6 (m), 131.1 (t, $^3J_{13C\text{-}19F}$ = 10.7 Hz), 114.1 (d, $^3J_{13C\text{-}13C}$ = 5.9 Hz), 112.0 (ddd, $J_{13C\text{-}19F}$ = 19.5, 5.9, 1.8Hz), 103.4 (t, $^2J_{13C\text{-}19F}$ = 18.8 Hz), 87.0 (dd, $^1J_{13C\text{-}13C}$ = 184 Hz, $^2J_{13C\text{-}13C}$ = 15.8 Hz), 81.4 (dd, $^1J_{13C\text{-}13C}$ = 79.5 Hz, $^1J_{13C\text{-}13C}$ = 184 Hz), 55.4, 53.9 (dd, $^1J_{13C\text{-}13C}$ = 79.9 Hz, $^2J_{13C\text{-}13C}$ = 16.2 Hz). **^{19}F NMR ($CDCl_3$, 376 MHz,):** δ_F -109.7. **HRMS (ESI) m/z:** $[M+Na]^+$ calcd. for $C_{15}{}^{13}C_3H_{12}F_2N_2O_3Na$ 368.0809; Found 368.0811.

S11. Spectroscopic characterization of the silver vinylcarbene species

General procedure for the preparation of the silver vinylcarbene species ^{13}C-3h and ^{13}C-3n.

NMR monitoring. Inside the glovebox, [Tp$^{(CF3)2,Br}$Ag(THF)] complex (10 mg, 9.6 μmol, 1.1 equiv.) was dissolved in dry $CDCl_3$ (500 μL) and the solution was placed in an NMR tube. The tube was capped with a septum and taken out of the glovebox. The NMR tube was placed inside the 400.13 MHz NMR spectrometer and cooled down to 233 K. After reaching thermal equilibrium, the appropriate diazo compound (**1n, ^{13}C-1n** or **^{13}C-1h**) (1 equiv., 8.7 μmols) in dry $CDCl_3$ (100 μL) was added at once to the NMR tube. The corresponding silver vinylcarbene species (**3n, ^{13}C-3n** or **^{13}C-3h**) was quickly formed as ascertained by 1D and 2D NMR experiments.

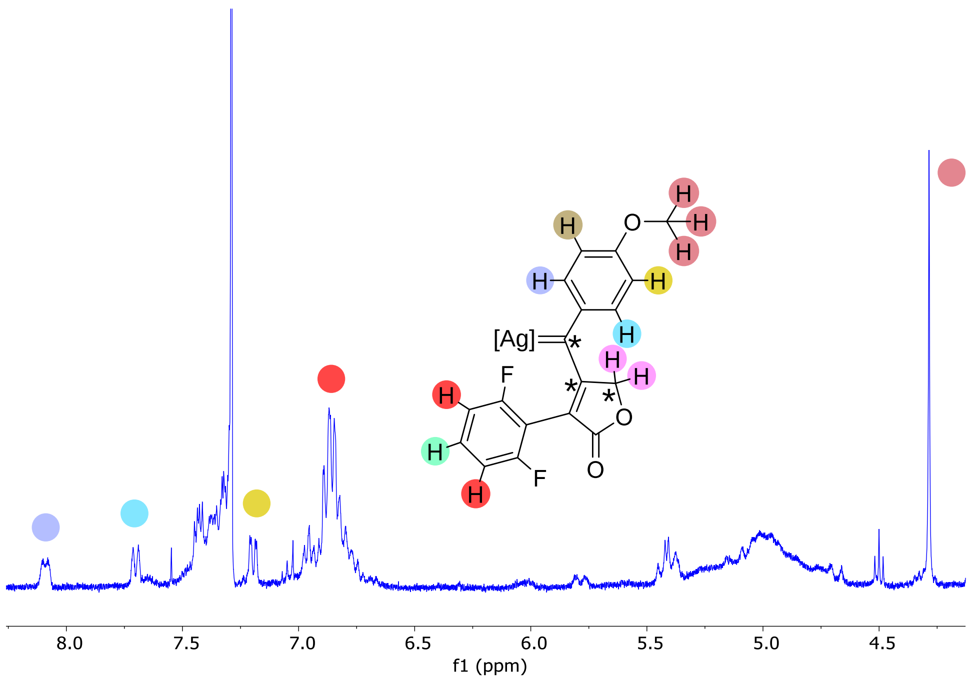

Figure S1. ^{1}H-NMR spectrum ($CDCl_3$, 400.13 MHz, 233 K) of **^{13}C-3n** ([Ag] = [Tp$^{(CF3)2,Br}$Ag]). ^{13}C-labelled carbons are marked with an asterisk in the scheme.

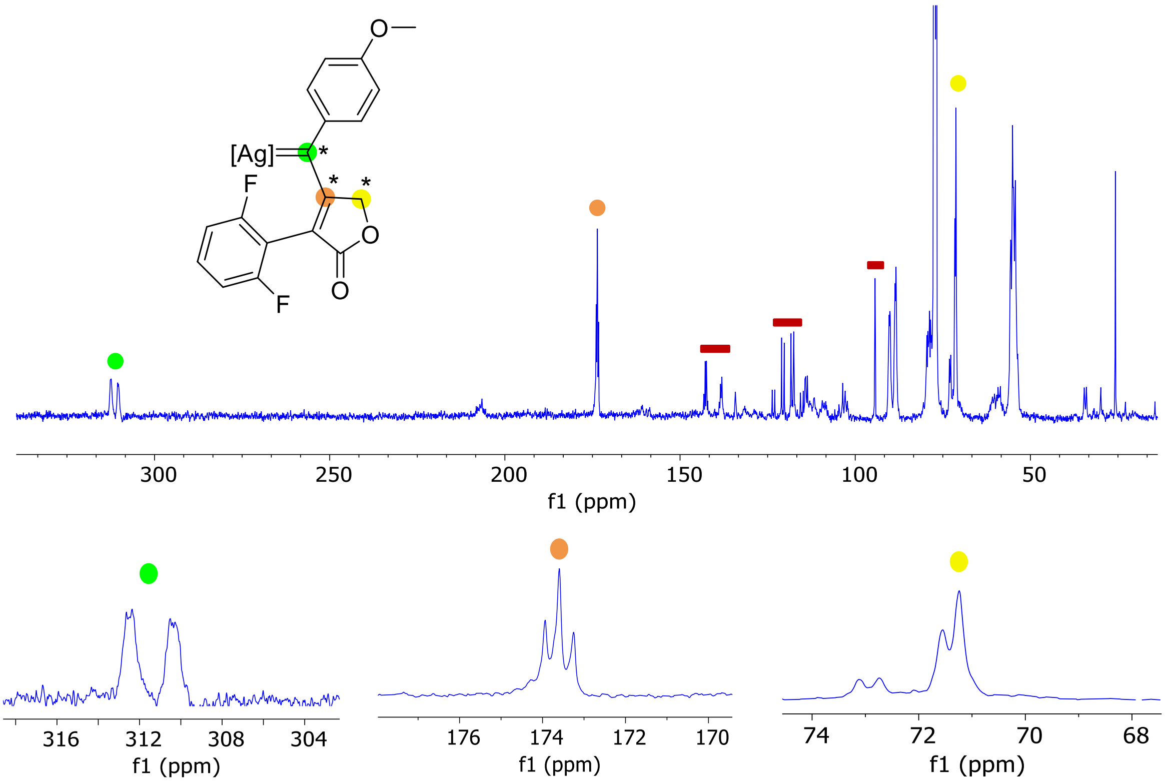

Figure S2. ^{13}C-NMR spectrum ($CDCl_3$, 100 MHz, 233 K) of **^{13}C-3n** ([Ag] = [Tp$^{(CF3)2,Br}$Ag]). ^{13}C-labelled carbons are marked with an asterisk in the scheme and assigned (coloured dots) in the spectrum. Carbon atoms of Tp$^{(CF3)2,Br}$ ligand are also highlighted with brown rectangles.

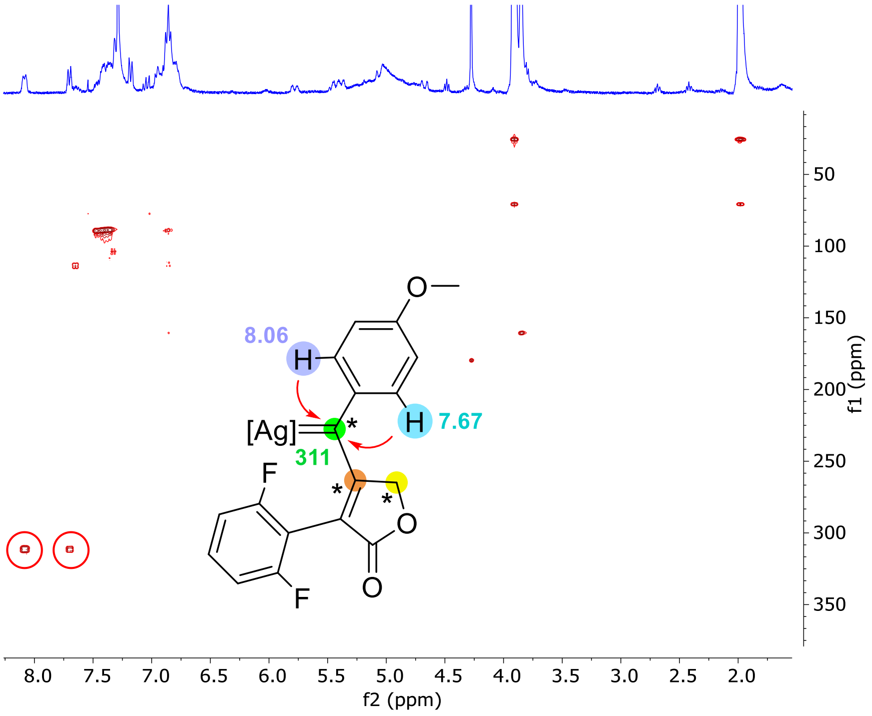

Figure S3. 2D HMBC spectrum ($CDCl_3$, 400.13 MHz, 233 K) of **^{13}C- 3n** ([Ag] = [Tp$^{(CF3)2,Br}$Ag]) showing cross-peaks of the aromatic protons at δ = 8.06 and 7.67 ppm with ^{13}C-labelled vinylcarbene carbon at 311 ppm. ^{13}C-labelled peaks are marked with an asterisk in the scheme.

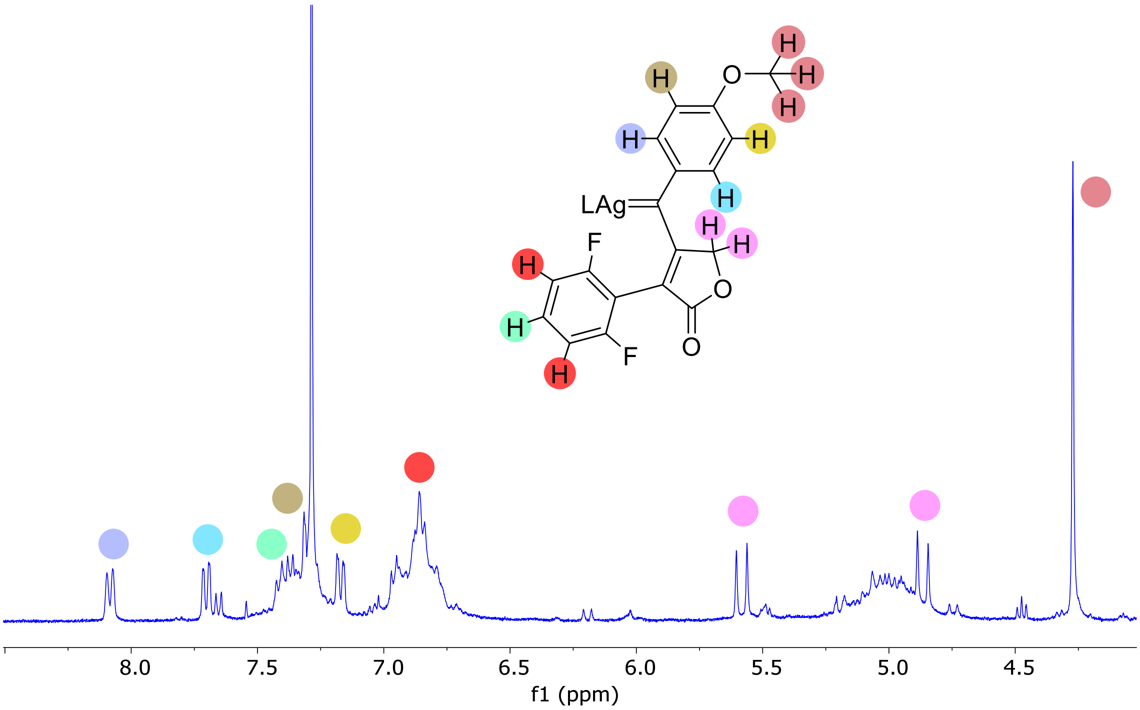

Figure S4. ^{1}H-NMR spectrum ($CDCl_3$, 400.13 MHz, 233 K) of **3n** ([Ag] = [Tp$^{(CF3)2,Br}$Ag]).

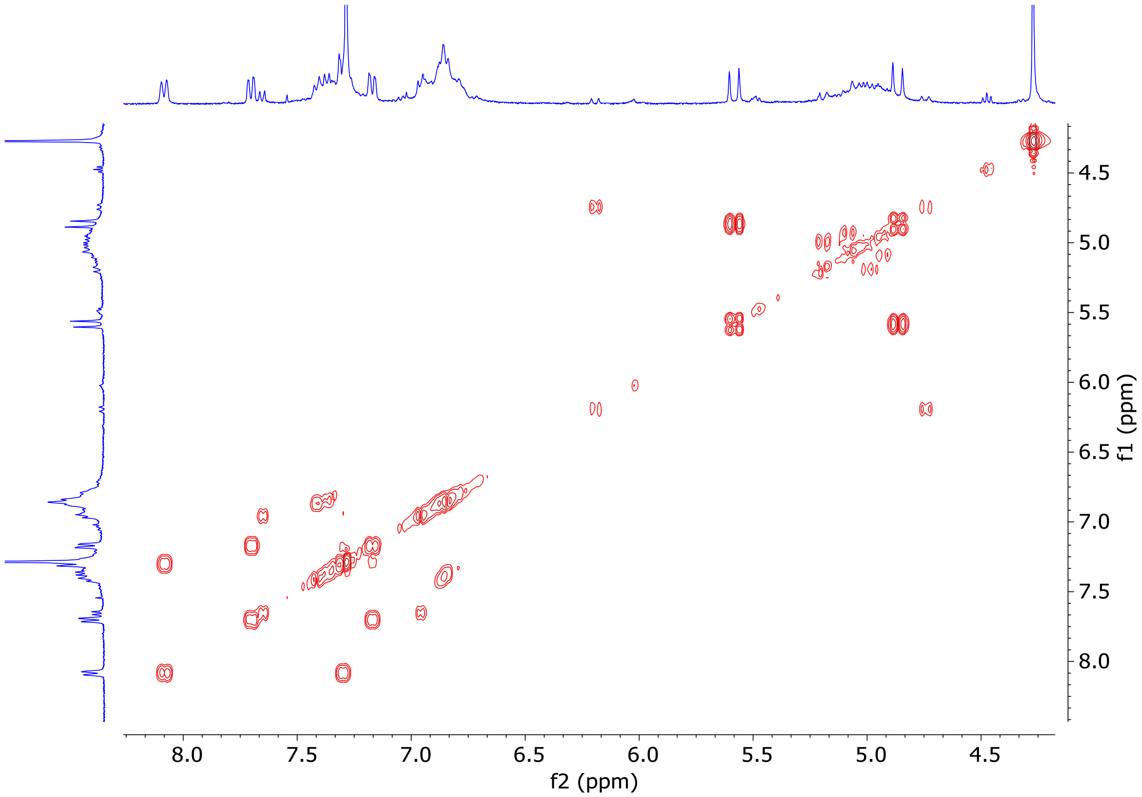

Figure S5. 2D COSY spectrum ($CDCl_3$, 400.13 MHz, 233 K) of **3n**.

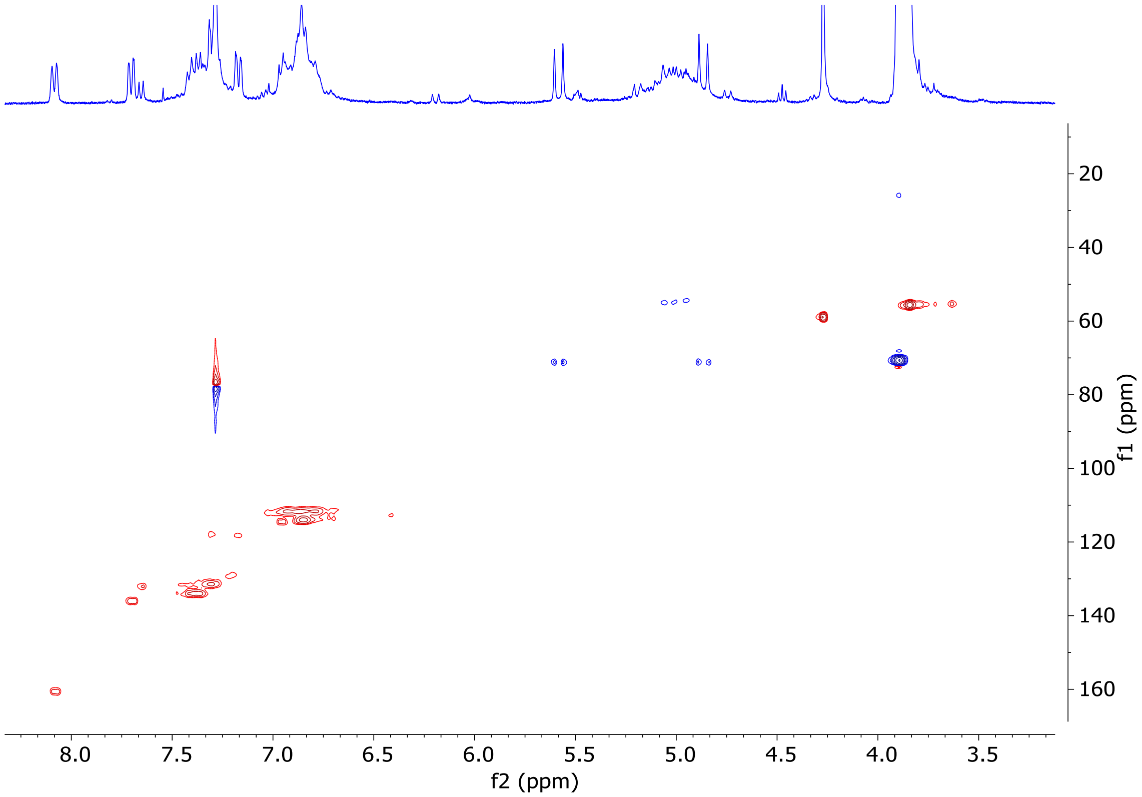

Figure S6. 2D HSQC spectrum ($CDCl_3$, 400.13 MHz, 233 K) of **3n**.

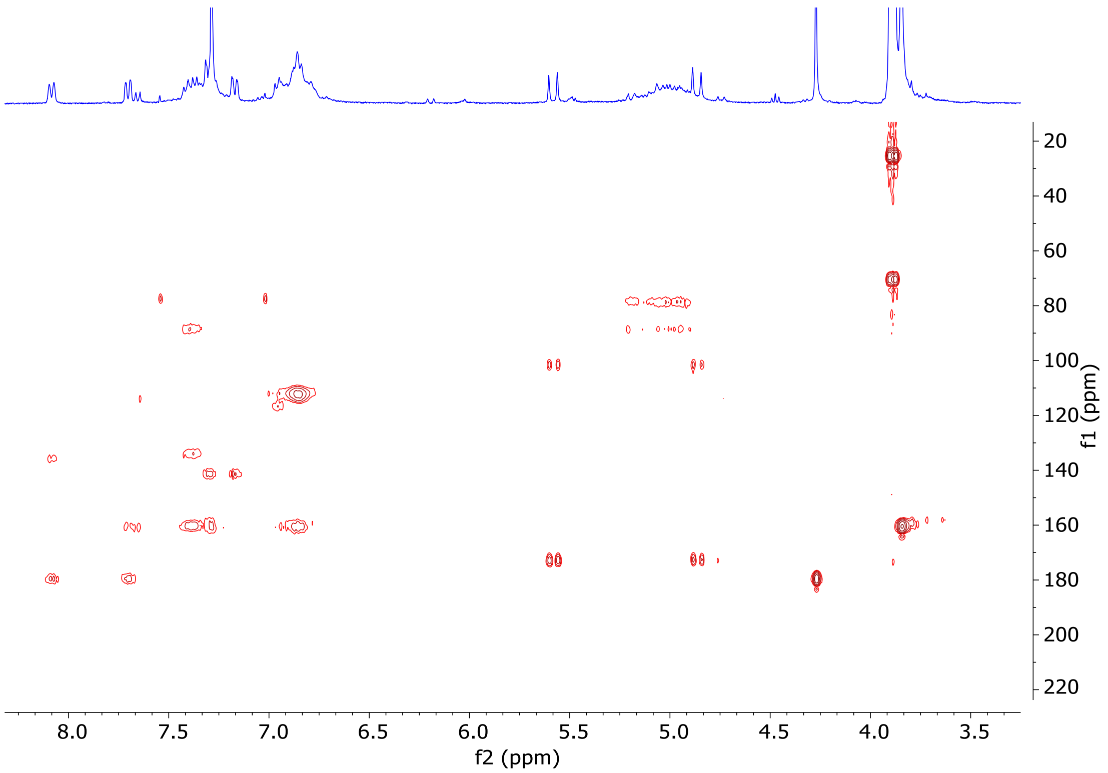

Figure S7. 2D HMBC spectrum ($CDCl_3$, 400.13 MHz, 233 K) of **3n**.

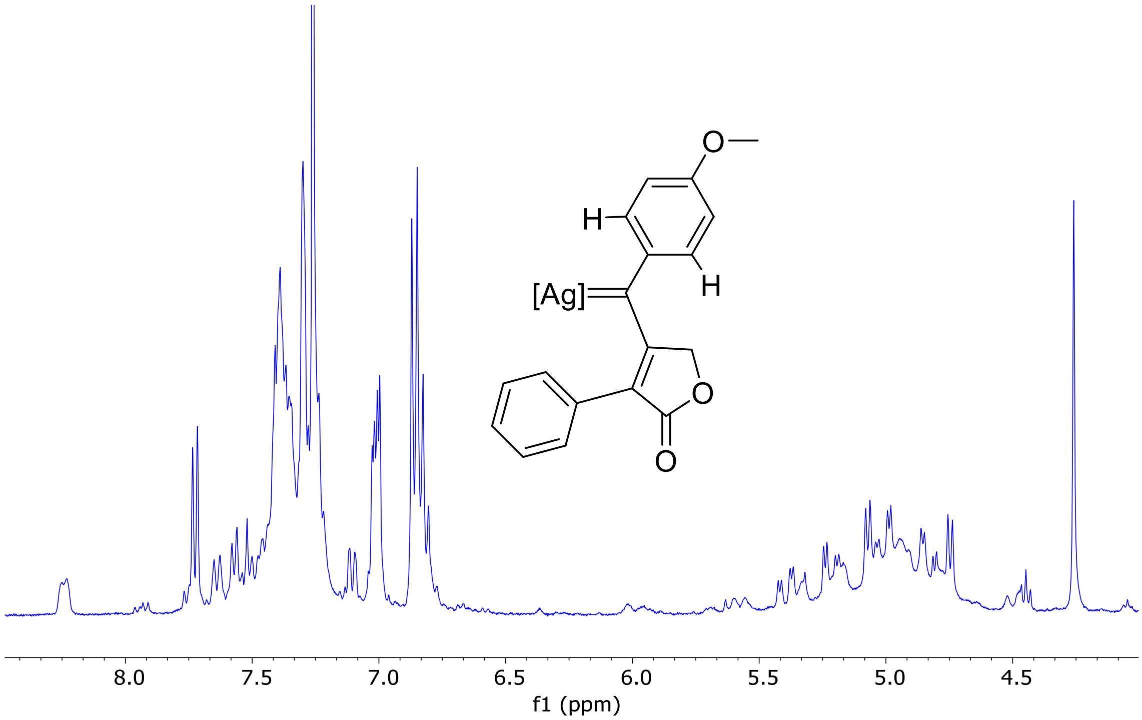

Figure S8. ^{1}H-NMR spectrum ($CDCl_3$, 400.13 MHz, 233 K) of **^{13}C-3h** ([Ag] = [$Tp^{(CF3)2,Br}Ag$]).

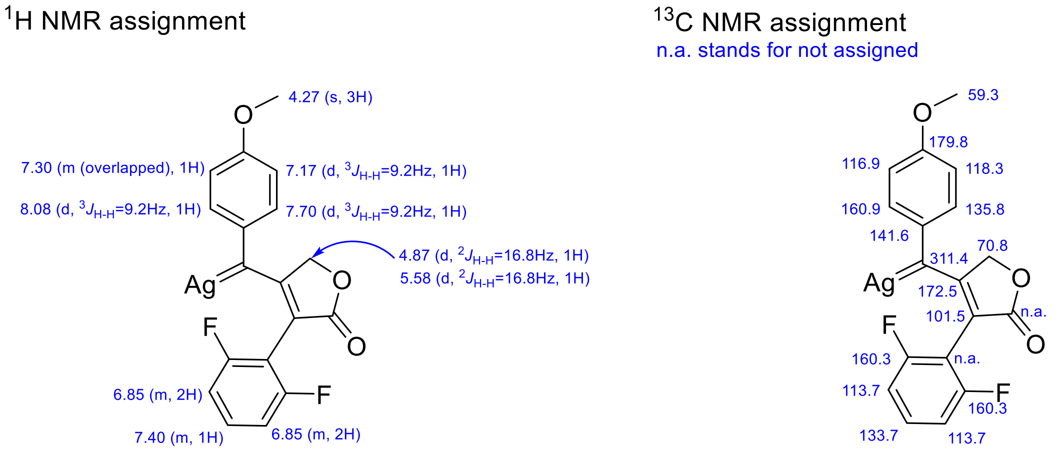

Figure S9. ^{1}H and ^{13}C full signal assignment for **3n**.

UV-vis monitoring. Inside the glovebox, [Tp$^{(CF3)2,Br}$Ag(THF)] complex (10 mg, 9.6 μmol, 1.1 equiv.) was dissolved in dry $CDCl_3$ (2 mL) and the solution was placed in a UV-vis cuvette. Afterwards, the quartz cuvette was capped with a septum and taken out from the glovebox to subsequently be placed in the Unisoku cryostat of the UV-vis spectrophotometer and cooled down to 233 K. After reaching thermal equilibrium, 1 equiv. of **^{13}C-1n** dissolved in dry $CDCl_3$ (100 μL of a 86mM solution) was added at once to generate **^{13}C-3n**. The formation of this species was followed by UV-vis spectroscopy, monitoring its characteristic absorption band at λ_{max} = 622 nm, which reached its maximum absorbance after 30 min approximately.

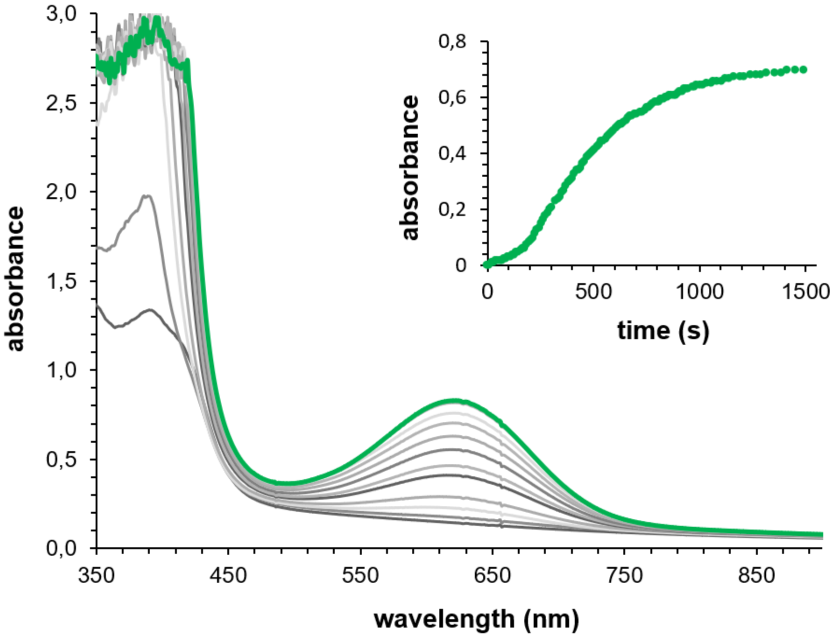

Figure S10. UV-Vis monitoring of the formation of the vinylcarbene **3n** by reaction of [Tp$^{(CF3)2,Br}$Ag(THF)] (4.8 mM) with **1n** at -40 ºC in $CDCl_3$. Inset: kinetic trace at 622 nm.

S12. Crystal structure of compound 4a

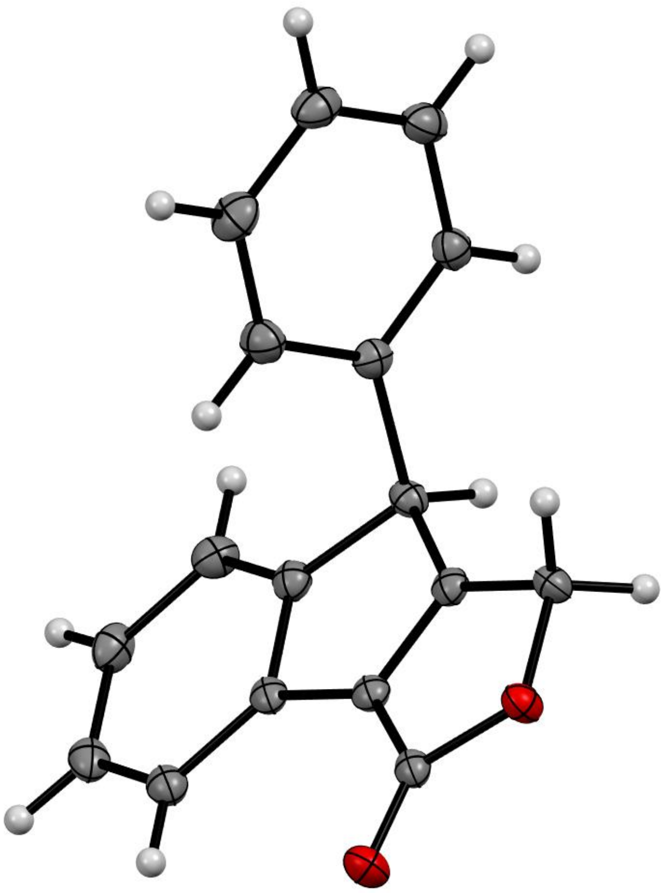

Figure S11. ORTEP plot (50% probability level) of the crystal structure for **4a**.

Colorless needle-like crystals of **4a** were grown at 2-6 °C upon slow diffusion of Et_2O into a solution of **4a** in chloroform.

A colorless prism-like specimen of $C_{17}H_{12}O_2$, approximate dimensions 0.100 mm x 0.250 mm x 0.340 mm, was used for the X-ray crystallographic analysis. The X-ray intensity data were measured on a D8 QUEST ECO three-circle diffractometer system equipped with a Ceramic x-ray tube (Mo Kα, λ = 0.71073 Å) and a doubly curved silicon crystal Bruker Triumph monochromator. A total of 242 frames were collected. The total exposure time was 1.34 hours. The integration of the data using a triclinic unit cell yielded a total of 12850 reflections to a maximum θ angle of 27.51° (0.77 Å resolution), of which 5482 were independent (average redundancy 2.344, completeness = 99.2%, R_{int} = 2.76%, R_{sig} = 4.29%) and 4033 (73.57%) were greater than $2\sigma(F^2)$. The final cell constants of a = 6.3718(3) Å, b = 11.7514(5) Å, c = 16.1542(6) Å, α = 95.714(2)°, β = 94.878(2)°, γ = 90.676(2)°, volume = 1198.97(9) $Å^3$, are based upon the refinement of the XYZ-centroids of 5092 reflections above 20 σ(I) with 6.697° < 2θ < 54.94°. The ratio of minimum to maximum apparent transmission was 0.873. The calculated minimum and maximum transmission coefficients (based on crystal size) are 0.9700 and 0.9910. The structure

was solved and refined using the Bruker SHELXTL Software Package, using the space group P -1, with Z = 4 for the formula unit, $C_{17}H_{12}O_2$.

The final anisotropic full-matrix least-squares refinement on F^2 with 343 variables converged at R1 = 4.58%, for the observed data and wR2 = 12.74% for all data. The goodness-of-fit was 1.020. The largest peak in the final difference electron density synthesis was 0.292 $e^-/Å^3$ and the largest hole was -0.242 $e^-/Å^3$ with an RMS deviation of 0.045 $e^-/Å^3$. On the basis of the final model, the calculated density was 1.375 g/cm^3 and F(000), 520 e^-.

Sample and crystal data for AD117

**Identification code**	AD117	
**Chemical formula**	C17H12O2	
**Formula weight**	248.27 g/mol	
**Temperature**	100(2) K	
**Wavelength**	0.71073 Å	
**Crystal size**	0.100 x 0.250 x 0.340 mm	
**Crystal habit**	colorless prism	
**Crystal system**	triclinic	
**Space group**	P -1	
**Unit cell dimensions**	a = 6.3718(3) Å	α = 95.714(2)°
	b = 11.7514(5) Å	β = 94.878(2)°
	c = 16.1542(6) Å	γ = 90.676(2)°
**Volume**	1198.97(9) Å3	
**Z**	4	
**Density (calculated)**	1.375 g/cm3	
**Absorption coefficient**	0.089 mm-1	
**F(000)**	520	

Data collection and structure refinement for AD117

**Diffractometer**	D8 QUEST ECO three-circle diffractometer
**Radiation source**	Ceramic x-ray tube (Mo Kα, λ = 0.71073 Å)
**Theta range for data collection**	2.94 to 27.51°
**Index ranges**	-8<=h<=8, -15<=k<=14, -19<=l<=20
**Reflections collected**	12850
**Independent reflections**	5482 [R(int) = 0.0276]
**Coverage of independent reflections**	99.20%
**Absorption correction**	none
**Max. and min. transmission**	0.9910 and 0.9700

**Structure solution technique**	direct methods
**Structure solution program**	SHELXS-97 (Sheldrick 2008)
**Refinement method**	Full-matrix least-squares on F2
**Refinement program**	SHELXL-2014 (Sheldrick 2014)
**Function minimized**	Σ w(Fo2 - Fc2)2
**Data / restraints / parameters**	5482 / 0 / 343
**Goodness-of-fit on F2**	1.02
**Final R indices**	4033 data; I>2σ(I) R1 = 0.0458, wR2 = 0.1158
	all data R1 = 0.0689, wR2 = 0.1274
**Weighting scheme**	w=1/[σ2(Fo2)+(0.0674P)2+0.2302P]
	where P=(Fo2+2Fc2)/3
**Largest diff. peak and hole**	0.292 and -0.242 eÅ-3
**R.M.S. deviation from mean**	0.045 eÅ-3

Atomic coordinates and equivalent isotropic atomic displacement parameters ($Å^2$) for 4a

U(eq) is defined as one third of the trace of the orthogonalized Uij tensor.

	x/a	y/b	z/c	U(eq)
O1	0.07400(16)	0.62400(9)	0.02770(6)	0.0173(2)
O13	0.79643(16)	0.67346(9)	0.09951(6)	0.0204(2)
C2	0.9752(2)	0.64125(12)	0.09999(9)	0.0150(3)
C3	0.1239(2)	0.60989(12)	0.16775(9)	0.0150(3)
C4	0.1397(2)	0.60351(12)	0.25819(9)	0.0149(3)
C5	0.0056(2)	0.63446(12)	0.31967(9)	0.0179(3)
C6	0.0742(3)	0.62107(13)	0.40235(9)	0.0206(3)
C7	0.2714(3)	0.57854(13)	0.42254(9)	0.0212(3)
C8	0.4068(3)	0.54708(13)	0.36079(9)	0.0194(3)
C9	0.3394(2)	0.55895(12)	0.27861(9)	0.0166(3)
C10	0.4560(2)	0.53188(12)	0.20053(9)	0.0150(3)
C11	0.2997(2)	0.57322(12)	0.13605(9)	0.0152(3)
C12	0.2827(2)	0.57905(12)	0.04448(9)	0.0164(3)
C14	0.5020(2)	0.40600(12)	0.17876(8)	0.0156(3)
C15	0.3419(2)	0.32398(13)	0.17797(10)	0.0212(3)
C16	0.3770(3)	0.21033(13)	0.15195(10)	0.0241(4)
C17	0.5716(3)	0.17722(13)	0.12662(9)	0.0223(3)
C18	0.7318(3)	0.25784(13)	0.12850(9)	0.0217(3)

C19	0.6970(2)	0.37181(13)	0.15480(9)	0.0180(3)
O1A	0.43044(16)	0.86263(9)	0.48599(6)	0.0185(2)
O13A	0.70335(17)	0.81065(9)	0.41218(7)	0.0219(3)
C2A	0.5249(2)	0.84281(12)	0.41276(9)	0.0167(3)
C3A	0.3716(2)	0.87197(12)	0.34562(9)	0.0155(3)
C4A	0.3528(2)	0.87732(12)	0.25504(9)	0.0150(3)
C5A	0.4863(2)	0.84751(12)	0.19302(9)	0.0182(3)
C6A	0.4152(3)	0.86204(13)	0.11064(9)	0.0209(3)
C7A	0.2168(3)	0.90410(13)	0.09133(9)	0.0213(3)
C8A	0.0829(3)	0.93394(13)	0.15366(9)	0.0194(3)
C9A	0.1523(2)	0.92135(12)	0.23557(9)	0.0162(3)
C10A	0.0382(2)	0.94910(12)	0.31438(9)	0.0153(3)
C11A	0.1974(2)	0.90890(12)	0.37851(9)	0.0155(3)
C12A	0.2192(2)	0.90528(13)	0.47032(9)	0.0169(3)
C14A	0.9908(2)	0.07538(12)	0.33195(8)	0.0152(3)
C15A	0.1543(2)	0.15342(13)	0.36036(9)	0.0197(3)
C16A	0.1135(3)	0.26848(13)	0.37627(10)	0.0229(3)
C17A	0.9097(3)	0.30732(13)	0.36466(9)	0.0211(3)
C18A	0.7467(3)	0.23050(13)	0.33716(9)	0.0215(3)
C19A	0.7872(2)	0.11497(13)	0.32099(9)	0.0184(3)

S13. Computational details

All density functional theory static calculations were carried out with the Gaussian 16 software package.[6] The geometry optimization calculations without symmetry constraints were performed with the BP86 functional Becke and Perdew,[7,8,9] together with the Grimme D3 correction term to the electronic energy.[10] For main-group atoms, the electronic configuration was described with the double-Z basis set with polarization of Ahlrichs (Def2-SVP keyword in Gaussian),[11] whereas for silver and iodine atoms, the small-core quasi-relativistic Stuttgart/Dresden effective core potential, with an associated valence basis set were employed (standard SDD keyword in Gaussian).[12,13,14] Analytical frequency calculations were performed to characterize the located stationary points. The frequencies were used to compute unscaled zero-point energies (ZPEs), thermal corrections, and entropy effects at 298 K. The accuracy of the calculations was improved by performing single point calculations with the Def2-TZVP basis set for main-group atoms,[15] the hybrid exchange-correlation B3LYP functional,[16,17] together with the Grimme D3 correction term. Solvent corrections were considered using the universal solvation model SMD of Cramer and Truhlar,[18] using dichloromethane as the solvent. In summary, the reported Gibbs energies are obtained at the B3LYP-D3/Def2-TZVP~SDD-SMD(DCM)//BP86-D3/Def2-SVP~SDD level of theory together with gas-phase thermal and entropic contributions computed at 298 K and 1 atm with the BP86-D3/Def2-SVP~SDD method.

The nuclear magnetic resonance (NMR) calculations were performed using the ORCA 5.0.4 program package.[19] Geometry optimizations and property calculations used the TPSSh meta-hybrid density functional[20] combined with the relativistic ZORA Hamiltonian.[21,22] The def2-TZVPP basis set was employed for all atoms except silver,[23] which used the decontracted SARC-ZORA-TZVPP basis set with an effective core potential (DelECP).[24] Auxiliary basis sets were generated using the AutoAux procedure.[25] Coulomb and exchange integrals were evaluated with the DEFGRID3 integration grid, ensuring improved numerical accuracy. VeryTightSCF convergence settings were used for the SCF cycles. Solvation effects were included via the conductor-like polarizable continuum model (CPCM) with chloroform as the solvent.[26] NMR shielding constants were calculated with the EPR-NMR approach implemented in ORCA (TAU = Dobson, Nuclei = all carbon).[27]

A separate file contains the xyz coordinates for all the optimized structures.

S14. ^{13}C NMR Calculations

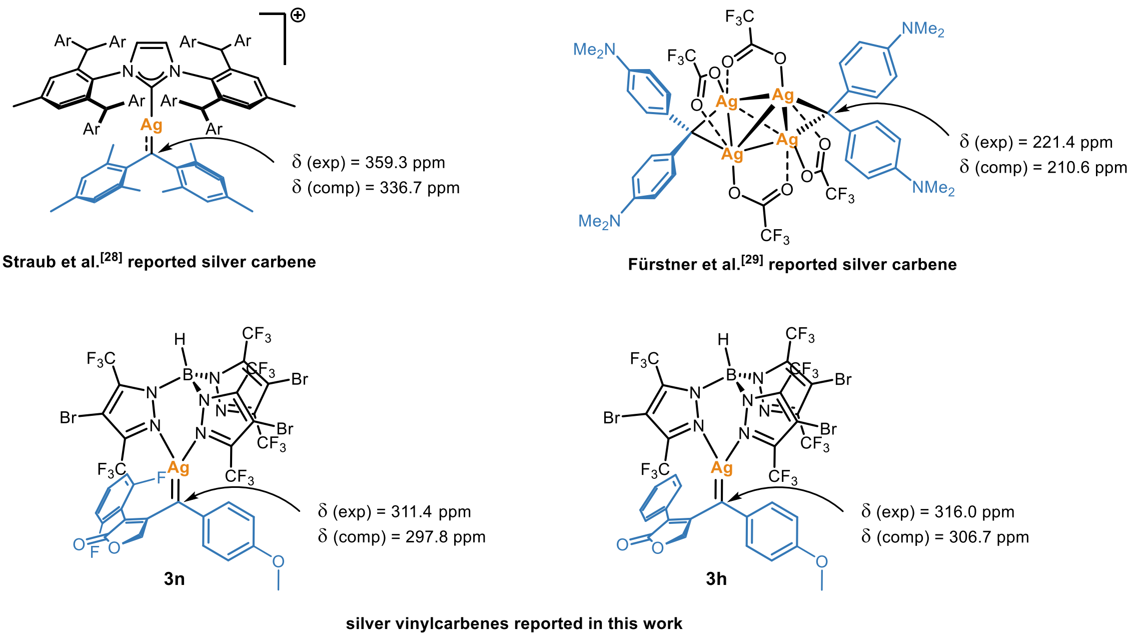

Figure S12. Computed and experimental **^{13}C NMR** chemical shifts for the carbene carbon in silver carbene complexes.

S15. Bonding analysis

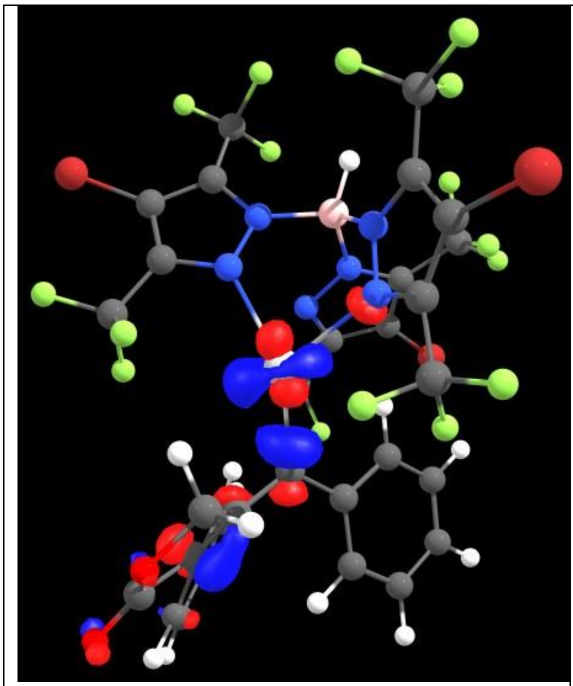	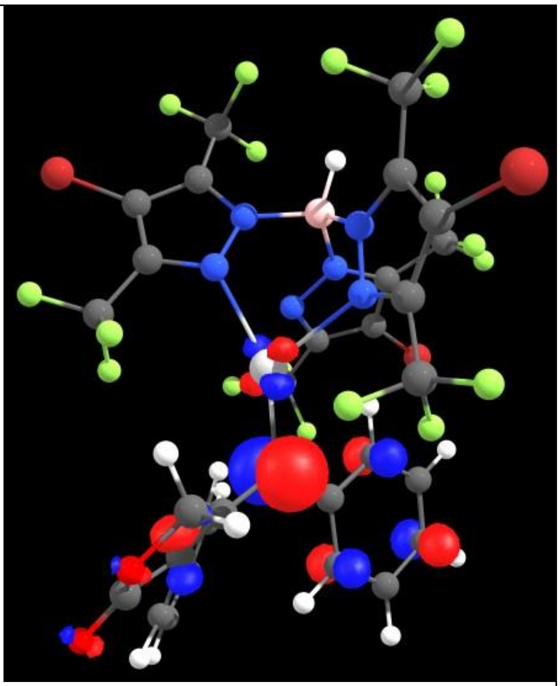
**Figure S13.** HOMO for intermediate **F**	**Figure S14.** LUMO for intermediate **F**

S16. Mechanism discussion

Formation and reactivity of vinylcarbene species F

To yield vinylcarbene species **F**, the initial diazo compound **A** can evolve through four different paths as discussed in our previous work on silver vinylcarbenes.[30] The first three paths consisting on (a) cyclopropene, (b) cyclobutene, and (c) ylide intermediates share the initial step of the transformation. The coordination of the silver complex to the carbenic carbon of the diazo species is a mildly endergonic process by 4.2 kcal/mol. Then, the extrusion of nitrogen takes place through **TS-BC** with a kinetic cost of 12.2 kcal/mol relative to starting material **A**, resulting in the formation of silver carbene species **C** with a relative Gibbs energy of -15.7 kcal/mol. On the other hand, the fourth path consisting on π-activation involves the coordination of the silver catalyst to the triple bond of diazo species **A**, forming intermediate **B1**, which has a relative energy of 1.5 kcal/mol relative to isolated reactants. Subsequently, the C-C bond formation occurs via transition state **TS-B1C1**, with an energy barrier of 18.5 kcal/mol (see Figure S15). Therefore, since the energy barrier for **TS-B1C1** is 6.3 kcal/mol higher than that for **TS-BC**, the pi-activation mechanism can be disregarded.

Once the carbene species **C** is generated, a nucleophilic attack of the alkyne onto the carbenic carbon induces a 5-exo-dig cyclization, yielding zwitterionic vinyl cationic species **D**. Formation of the cyclopropane intermediate through **TS-DE1** has a kinetic cost 20.5 kcal/mol. Given the energetic barriers of 11.0 and 0.4 kcal/mol for **TS-DE** and **TS-EF**, respectively, for the evolution from ylide intermediate **D** to the vinylcarbene species **F**, all the described paths were discarded and the overall formation of silver η^1-vinylcarbene **F** is postulated based on the mechanism depicted in the manuscript.

IRC analysis of the concerted CH insertion pathway

The IRC of the CH insertion pathway was analyzed. The results, shown in Figure S16, clearly indicate that the reaction proceeds through a synchronous pathway for the concerted pathway (TS-FG).

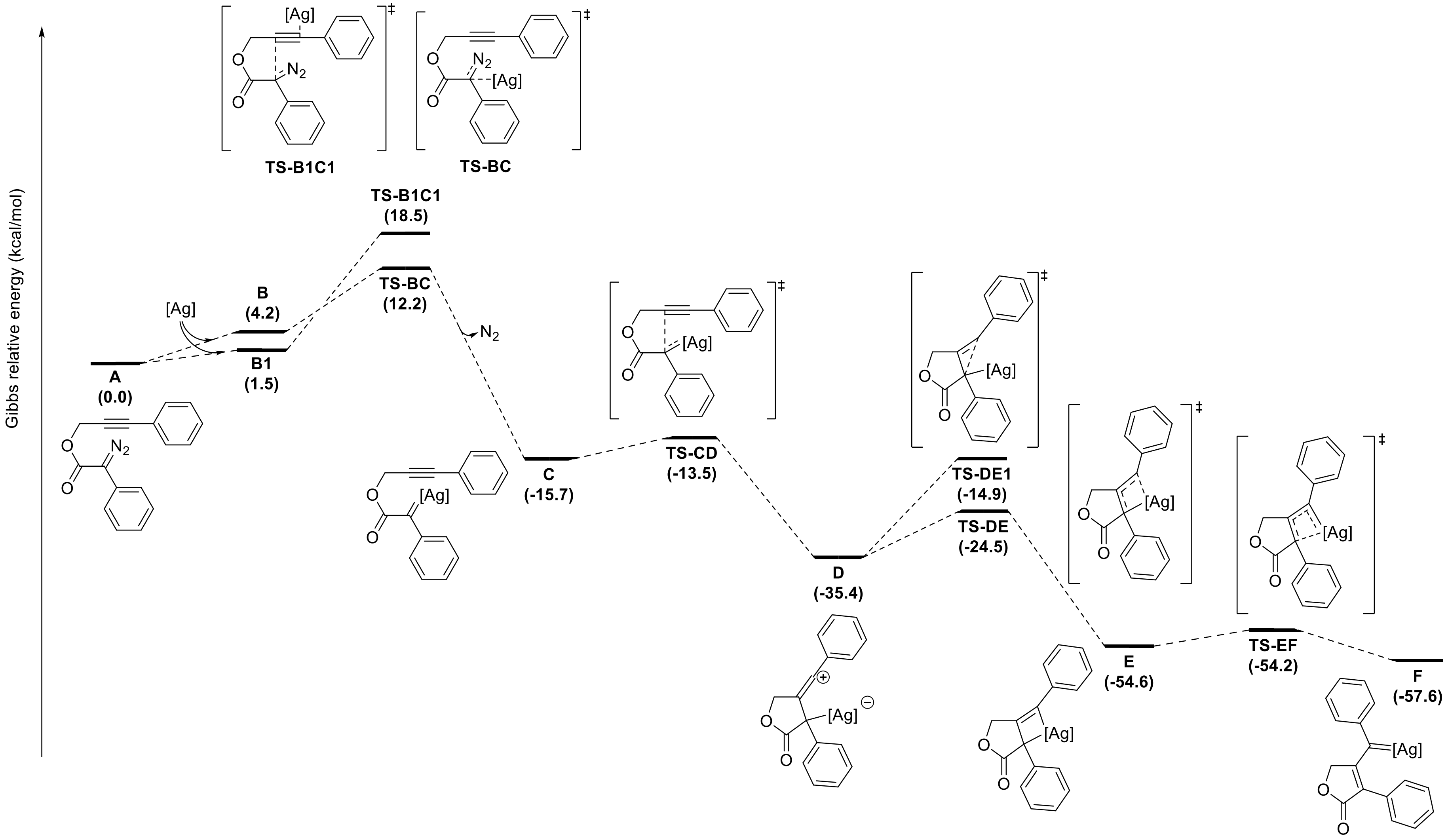

Figure S15. Gibbs energy profile (in kcal/mol) of the studied pathways for the formation of the vinylcarbene species **F** ([Ag] = Tp$^{(CF3)2,Br}$Ag).

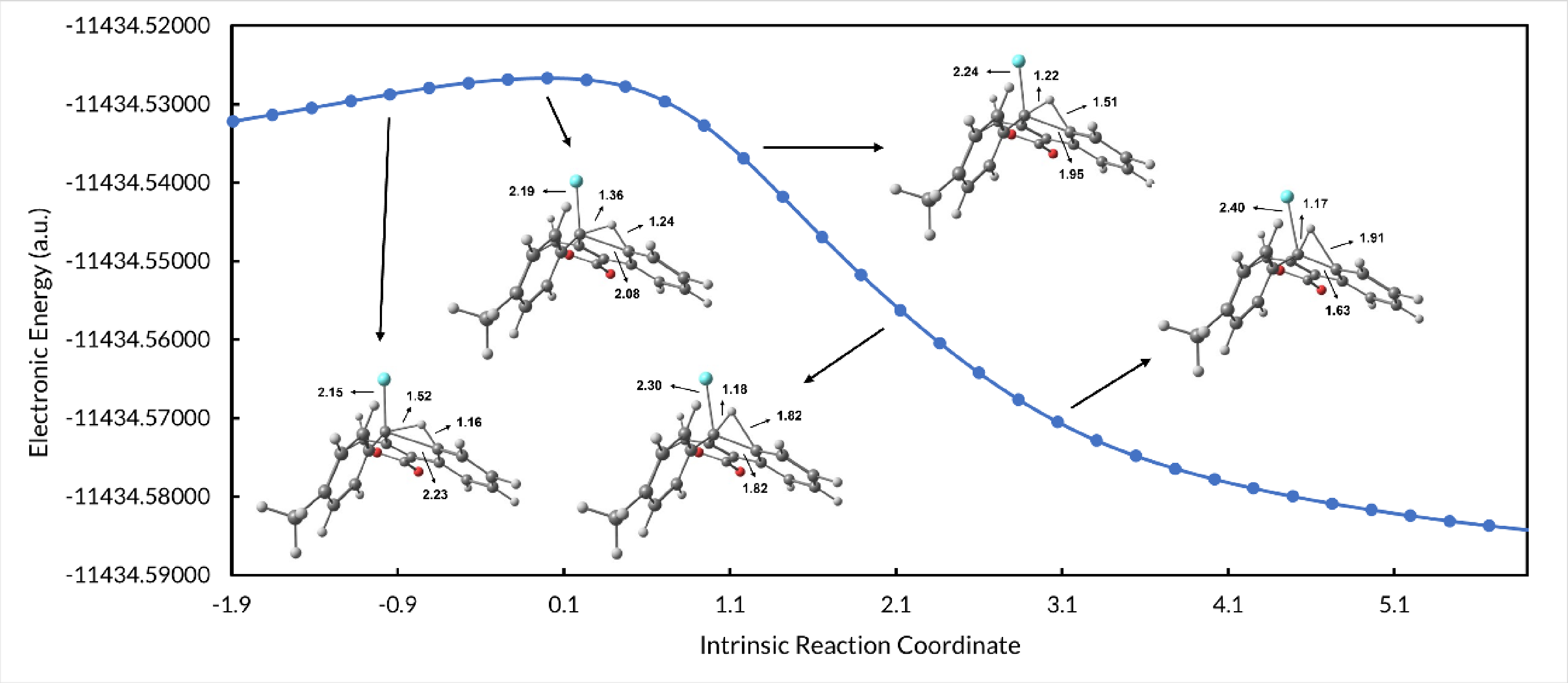

Figure S16. IRC for the concerted C-H pathway (TS-FG). Representative structures and key bond distances (in Å) are shown. The Tp$^{(CF3)2,Br}$ ligand is omitted for clarity.

Stepwise and concerted computed barriers using $[Tp^{Br3}Ag]_2$

Table S1. Computed barriers for the concerted and stepwise pathways using the $[Tp^{Br3}Ag]_2$ when R^2 = OMe, Me and F in intermediate F.

R	ΔG‡ concerted	ΔG‡ stepwise
***p*-OMe**	22.2	25.7
***p*-Me**	17.3	-
***p*-F**	19.5	20.6

S17. References

[1] M. Bao, Y. Qian, H. Su, B. Wu, L. Qiu, W. Hu and X.Xu. Gold(I)-Catalyzed and H_2O-Mediated Carbene Cascade Reaction of Propargyl Diazoacetates: Furan Synthesis and Mechanistic Insights. *Org. Lett.* **2018**, *20*, 5332-5335.

[2] M. Bao, X. Wang, L. Qiu, W. Hu, P.W. Hong Chan and X. Xu. Gold-Catalyzed 1,2-Acyloxy Migration/Coupling Cascade of Propargyl Diazoacetates: Synthesis of Isomycin Derivatives. *Org. Lett.* **2019**, *21*,6, 1813-1817.

[3] J.M. Rodriguez, L. Nevola, N.T. Ross, G. Lee and A.D. Hamilton. Synthetic Inhibitors of Extended Helix–Protein Interactions Based on a Biphenyl 4,4′-Dicarboxamide Scaffold. *ChemBioChem* **2009**, *10*, 829 – 833.

[4] Z. Chen, P. Liang, X. Ma, H. Luo, G. Xu, T. Liu, X. Wen, J. Zheng and H. Ye. Catalyst-Free Annulation of 2-Pyridylacetates and Ynals with Molecular Oxygen: An Access to 3-Acylated Indolizines. *J. Org. Chem.* **2019**, *84*, 19, 12639–12647.

[5] H. Guo, Q. Zhang, W. Pan, H. Yang, K. Pei, J. Zhai, T. Li, Z. Wang, Y. Wang and Y. Yin. One-pot Synthesis of Substituted Pyrazoles from Propargyl Alcohols via Cyclocondensation of in situ-Generated α-Iodo Enones/Enals and Hydrazine Hydrate. *Asian J. Org. Chem.* **2021**, 10, 2231–2237.

[6] Gaussian 16, Revision C.01, Frisch, M. J.; Trucks, G. W.; Schlegel, H. B.; Scuseria, G. E.; Robb, M. A.; Cheeseman, J. R.; Scalmani, G.; Barone, V.; Petersson, G. A.; Nakatsuji, H.; Li, X.; Caricato, M.; Marenich, A. V.; Bloino, J.; Janesko, B. G.; Gomperts, R.; Mennucci, B.; Hratchian, H. P.; Ortiz, J. V.; Izmaylov, A. F.; Sonnenberg, J. L.; Williams-Young, D.; Ding, F.; Lipparini, F.; Egidi, F.; Goings, J.; Peng, B.; Petrone, A.; Henderson, T.; Ranasinghe, D.; Zakrzewski, V. G.; Gao, J.; Rega, N.; Zheng, G.; Liang, W.; Hada, M.; Ehara, M.; Toyota, K.; Fukuda, R.; Hasegawa, J.; Ishida, M.; Nakajima, T.; Honda, Y.; Kitao, O.; Nakai, H.; Vreven, T.; Throssell, K.; Montgomery, J. A., Jr.; Peralta, J. E.; Ogliaro, F.; Bearpark, M. J.; Heyd, J. J.; Brothers, E. N.; Kudin, K. N.; Staroverov, V. N.; Keith, T. A.; Kobayashi, R.; Normand, J.; Raghavachari, K.; Rendell, A. P.; Burant, J. C.; Iyengar, S. S.; Tomasi, J.; Cossi, M.; Millam, J. M.; Klene, M.; Adamo, C.; Cammi, R.; Ochterski, J. W.; Martin, R. L.; Morokuma, K.; Farkas, O.; Foresman, J. B.; Fox, D. J. Gaussian, Inc., Wallingford CT, **2016**.

[7] Becke, A. Density-functional exchange-energy approximation with correct asymptotic behaviour. *Phys. Rev. A: At. Mol. Opt. Phys.* **1988**, *38*, 3098−3100.

[8] Perdew, J.P. Density-functional approximation for the correlation energy of the inhomogeneous electron gas. *Phys. Rev. B: Condens. Matter Mater. Phys.* **1986**, *33*, 8822−8824.

[9] Perdew, J.P. Erratum: Density-functional approximation for the correlation energy of the inhomogeneous electron gas. *Phys. Rev. B: Condens. Matter Mater. Phys.* **1986**, *34*, 7406−7406.

[10] Grimme, S.; Antony, J.; Ehrlich, S.; Krieg, H. A consistent and accurate ab initio parametrization of density functional dispersion correction (DFT-D) for the 94 elements H–Pu, *J. Chem. Phys.* **2010**, *132*, 154104.

[11] Schäfer, S.; Horn, H.; Ahlrichs, R. Fully optimized contracted Gaussian basis sets for atoms Li to Kr, *J. Chem. Phys.* **1992**, *97*, 2571−2577.

[12] Haeusermann, U.; Dolg, M.; Stoll, H.; Preuss, H.; Schwerdtfeger, P.; Pitzer, S. Accuracy of energy-adjusted quasirelativistic ab initio pseudopotentials: all-electron and pseudopotential benchmark calculations for Hg, HgH and their cations. *Mol. Phys.* **1993**, *78*, 1211−1224.

[13] Küchle, W.; Dolg, M.; Stoll, H.; Preuss, H. Energy-adjusted pseudopotentials for the actinides. Parameter sets and test calculations for thorium and thorium monoxide. *J. Chem. Phys.* **1994**, *100*, 7535−7542.

[14] Leininger, T.; Nicklass, A.; Stoll, H.; Dolg, M.; Schwerdtfeger, P. The accuracy of the pseudopotential approximation. II. A comparison of various core sizes for indium pseudopotentials in calculations for spectroscopic constants of InH, InF, and InCl. *J. Chem. Phys.* **1996**, *105*, 1052−1059.

[15] Weigend, F.; Ahlrichs, R. Balanced basis sets of split valence, triple zeta valence and quadruple zeta valence quality for H to Rn: Design and assessment of accuracy. *Phys. Chem. Chem. Phys.* **2005**, *7*, 3297–3305.

[16] Becke, A.D. Density-functional thermochemistry. III. The role of exact exchange. *J. Chem. Phys.* **1993**, *98*, 5648–5652.

[17] Lee, C.; Yang, W.; Parr, R.G. Development of the Colle-Salvetti correlation-energy formula into a functional of the electron density. *Phys. Rev. B: Condens. Matter Mater. Phys.* **1988**, *37*, 785–789.

[18] Marenich, A.V.; Cramer, C.J.; Truhlar, D.G. Universal solvation model based on solute electron density and on a continuum model of the solvent defined by the bulk dielectric constant and atomic surface tensions. *J. Phys. Chem. B* **2009**, *113*, 6378–6396.

[19] Neese, F. Software update: the ORCA program system, version 5.0. *WIREs Comput. Mol. Sci.* **2022**, *12*, e1606.

[20] Tao, J.; Perdew, J. P.; Staroverov, V. N.; Scuseria, G. E. Climbing the density functional ladder: Nonempirical meta–generalized gradient approximation designed for molecules and solids. *Phys. Rev. Lett.* **2003**, *91*, 146401.

[21] Schreckenbach, G.; Ziegler, T. Calculation of NMR Shielding Tensors Using Gauge-Including Atomic Orbitals and Modern Density Functional Theory. *J. Phys. Chem.* **1995**, *99*, 606–611.

[22] van Lenthe, E.; Baerends, E. J.; Snijders, J. G. Relativistic Regular Two-Component Hamiltonians. *J. Chem. Phys.* **1993**, *99*, 4597–4610.

[23] Weigend, F.; Ahlrichs, R. Balanced basis sets of split valence, triple zeta valence and quadruple zeta valence quality for H to Rn: Design and assessment of accuracy. *Phys. Chem. Chem. Phys.* **2005**, *7*, 3297–3305.

[24] Rolfes, J. D.; Bahmann, H.; Neese, F. All-electron scalar relativistic basis sets for the elements Rb–Xe. *J. Chem. Theory Comput.* **2020**, *16*, 6988−7003.

[25] Stoychev, G. L.; Auer, A. A.; Neese, F. Automatic Generation of Auxiliary Basis Sets. *J. Chem. Theory Comput.* **2017**, *13*, 554–562.

[26] Cossi, M.; Barone, V.; Cammi, R.; Tomasi, J. Quantum chemistry and solvation models: Solvent effects on the electronic structure of molecules. *Chem. Phys. Lett.* **1996**, *255*, 327–335.

[27] Neese, F. Prediction of electron paramagnetic resonance g values using coupled perturbed Kohn–Sham theory. *J. Chem. Phys.* **2005**, *122*, 034107.

[28] Hussong, M. W.; Hoffmeister, W. T.; Rominger, F.; Straub B. F. Copper and Silver Carbene Complexes without Heteroatom-Stabilization: Structure, Spectroscopy, and Relativistic Effects. *Angew. Chem. Int. Ed.* **2015**, *54*, 10331−10335.

[29] Tskhovrebov, A. G.; Goddard, R.; Fürstner, A. Two Amphoteric Silver Carbene Clusters. *Angew. Chem. Int. Ed*. **2018**, *57*, 8089−8094.

[30] Díaz-Jiménez, À.; Monreal-Corona, R.; Poater, A.; Álvarez, M.; Borrego, E.; Pérez, P. J.; Caballero, A.; Roglans, A.; Pla-Quintana, A. Intramolecular Interception of the Remote Position of Vinylcarbene Silver Complex Intermediates by C(sp^3)-H Bond Insertion. *Angew. Chem. Int. Ed.* **2022**, *62*, e202215163.

S17 NMR Spectra

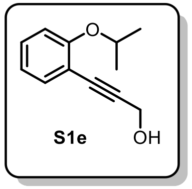

^{1}H NMR (400.13 MHz, $CDCl_3$)

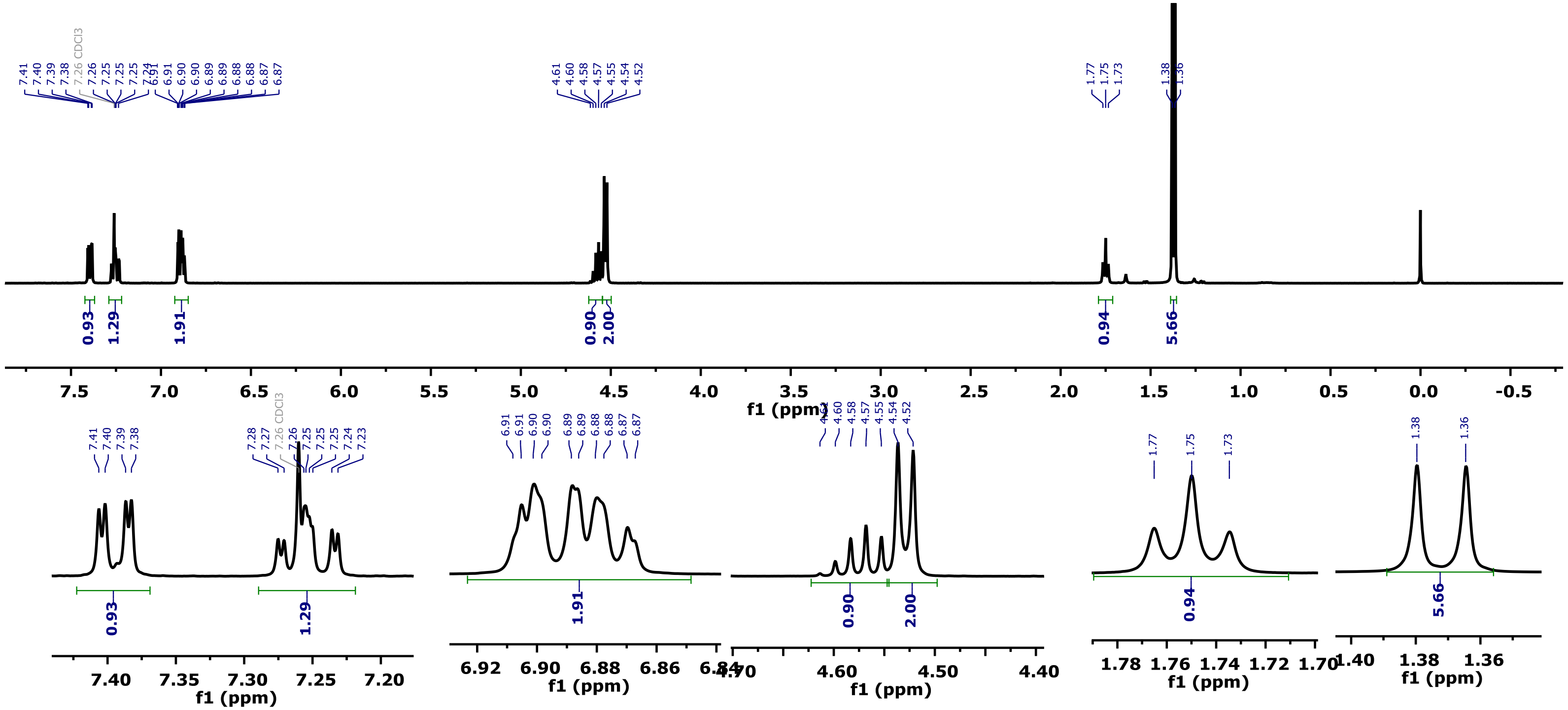

$^{13}C\{H\}$ NMR ($CDCl_3$, 100.6 MHz)

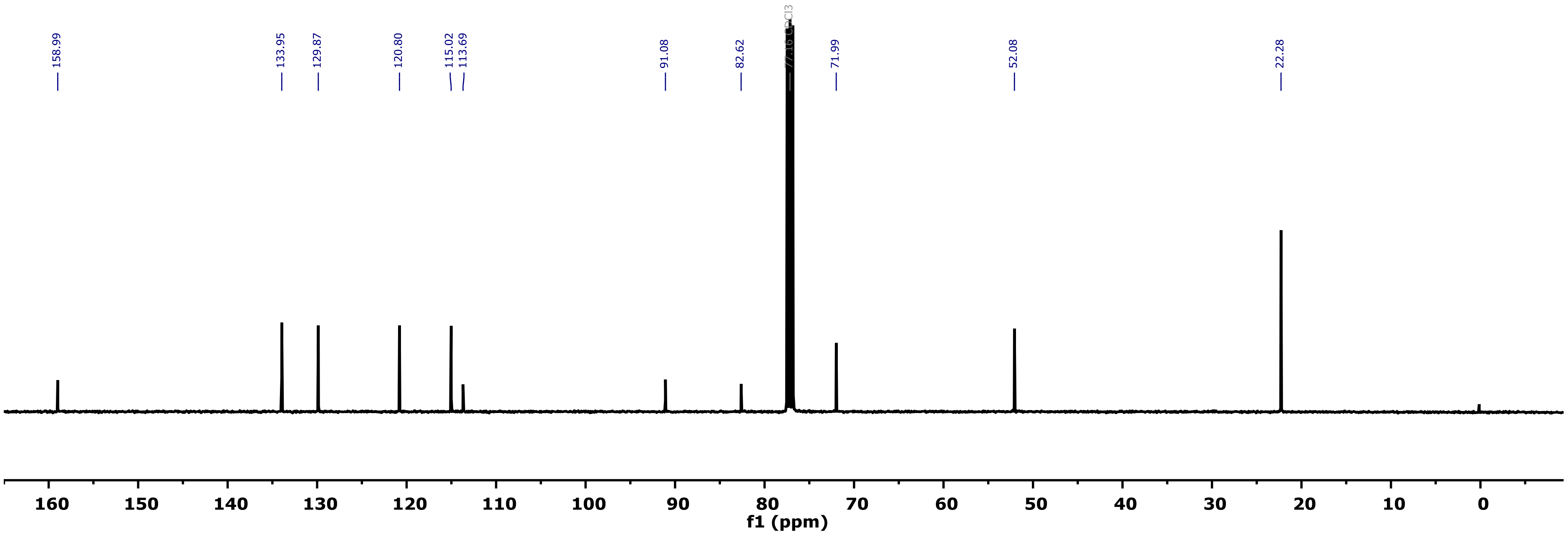

*

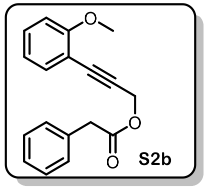

S2b

^{1}H NMR (400.13 MHz, $CDCl_3$)

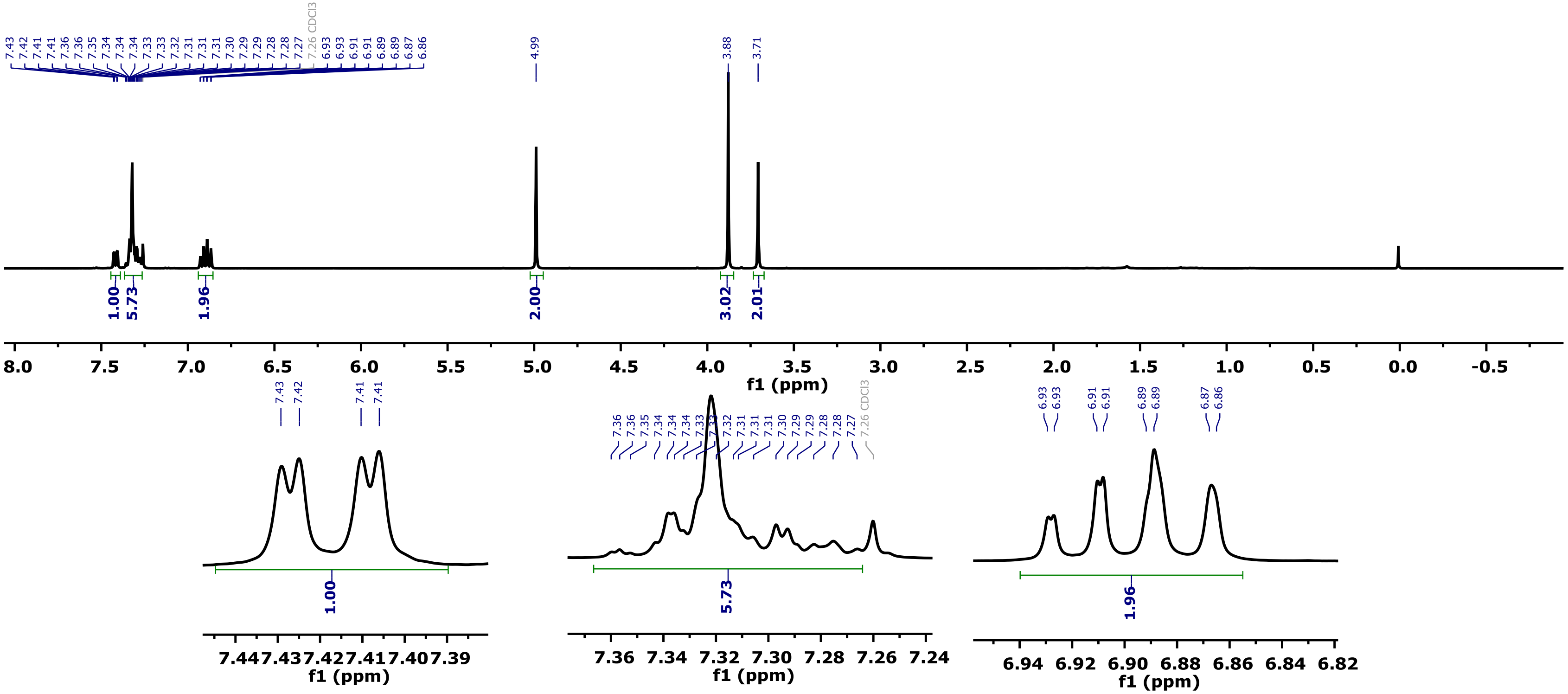

$^{13}C\{H\}$ NMR ($CDCl_3$, 100.6 MHz)

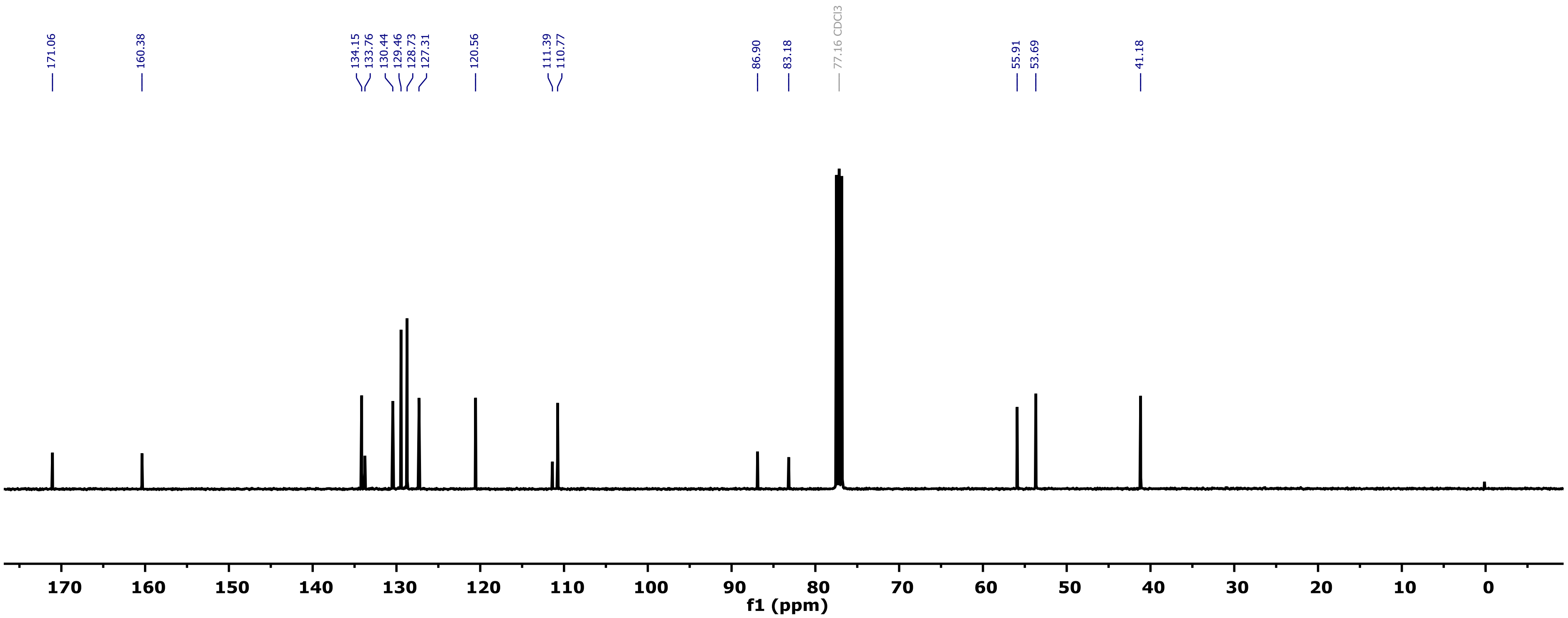

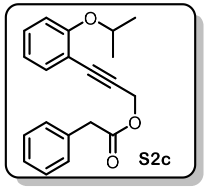

S2c

^{1}H NMR (400.13 MHz, $CDCl_3$)

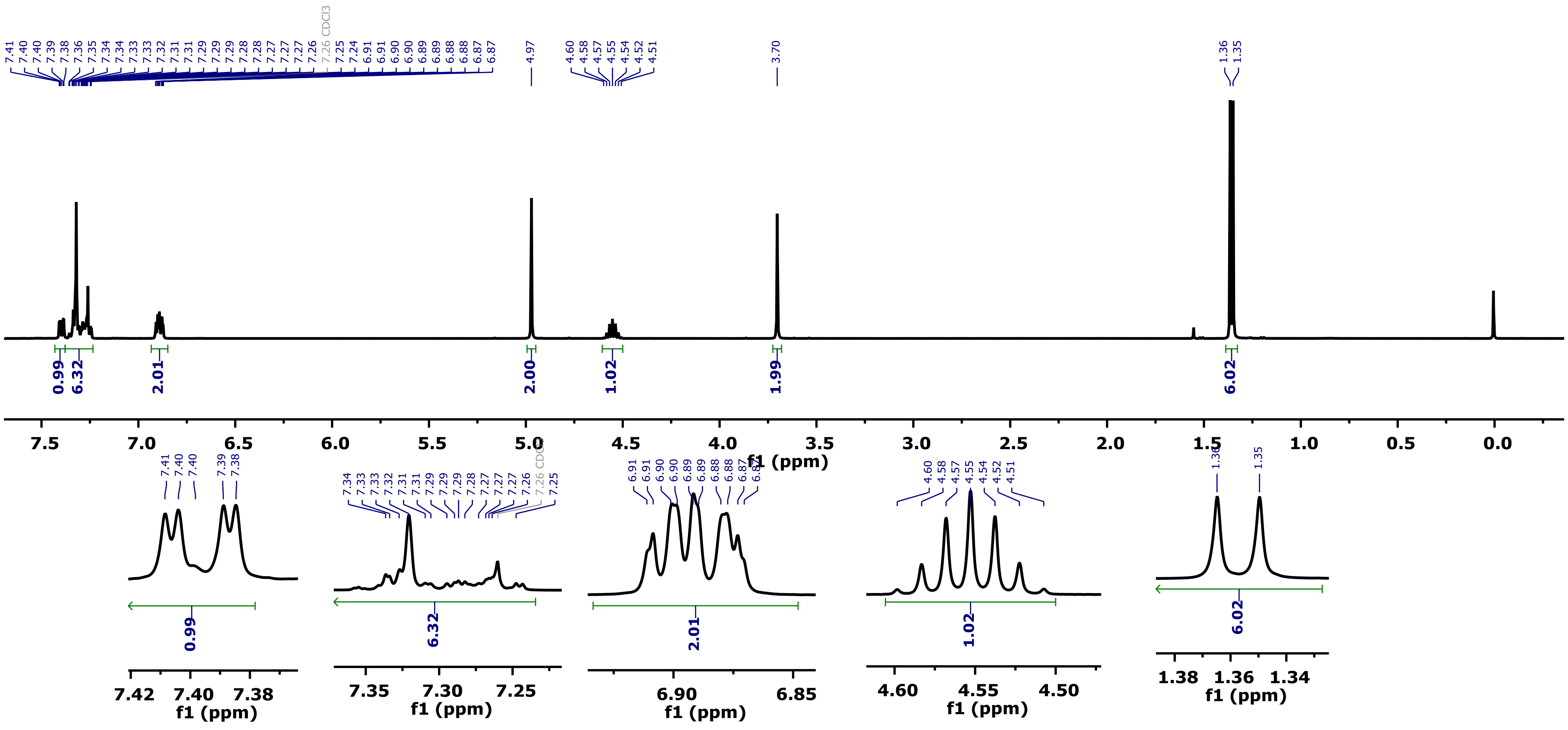

$^{13}C\{H\}$ NMR ($CDCl_3$, 100.6 MHz)

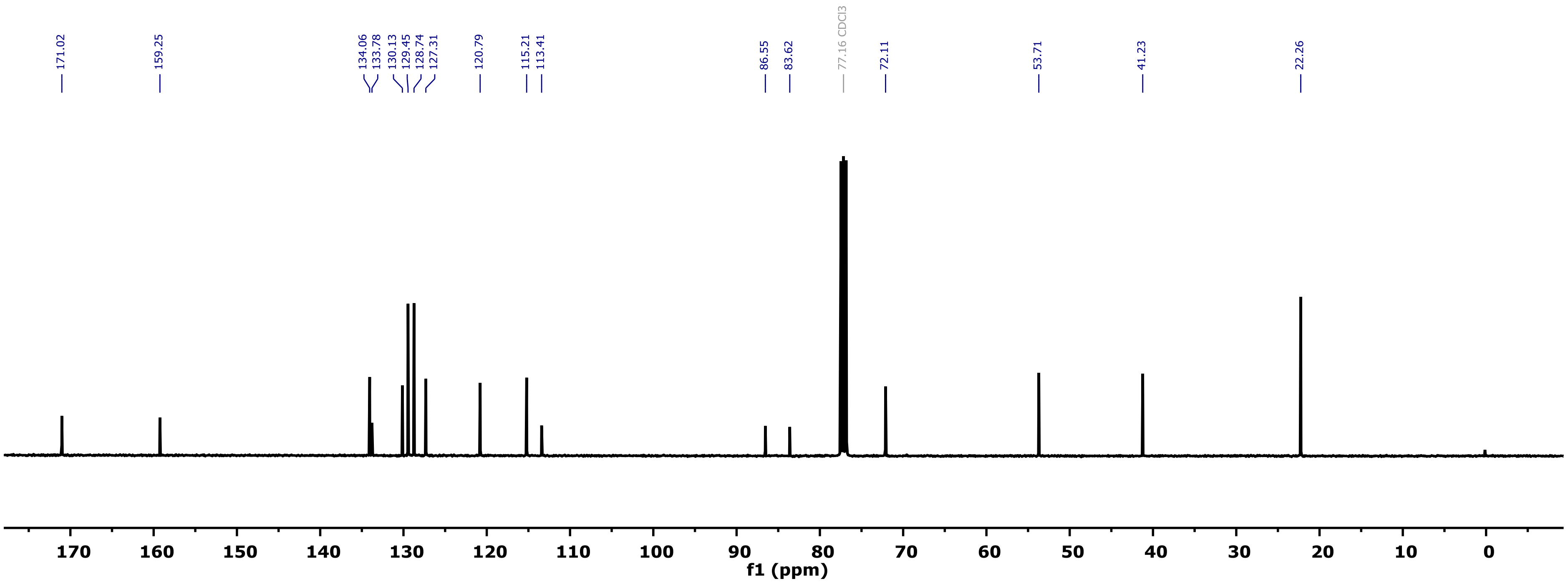

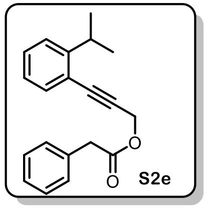

^{1}H NMR (400.13 MHz, $CDCl_3$)

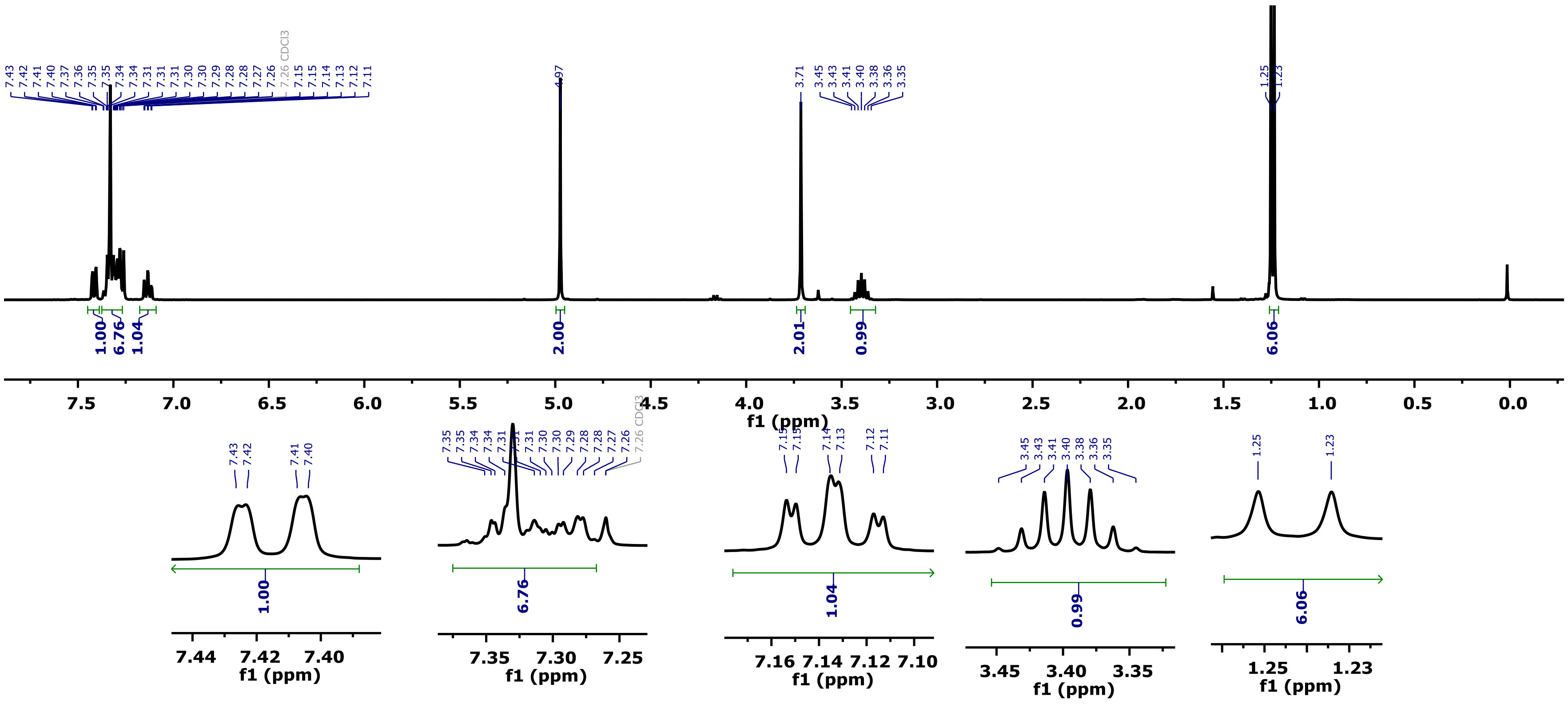

$^{13}C\{H\}$ NMR ($CDCl_3$, 100.6 MHz)

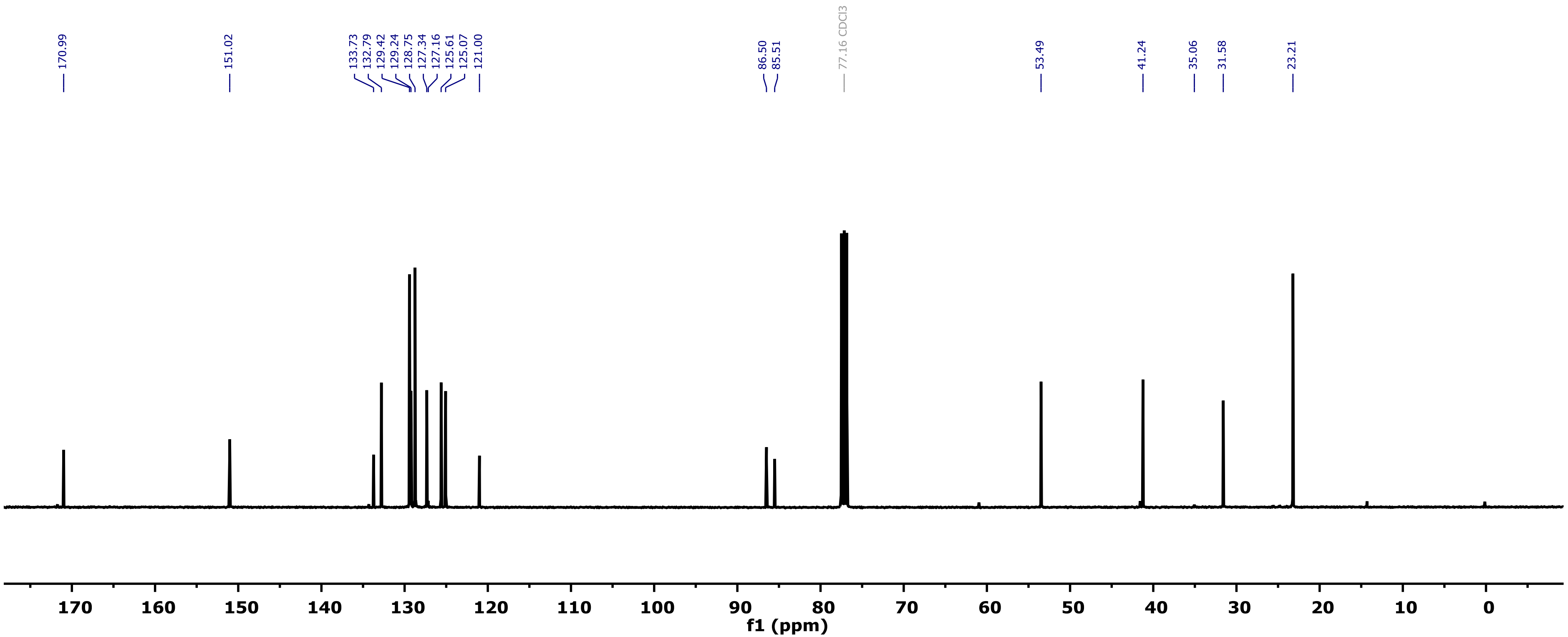

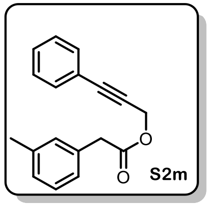
S2m

^{1}H NMR (400.13 MHz, $CDCl_3$)

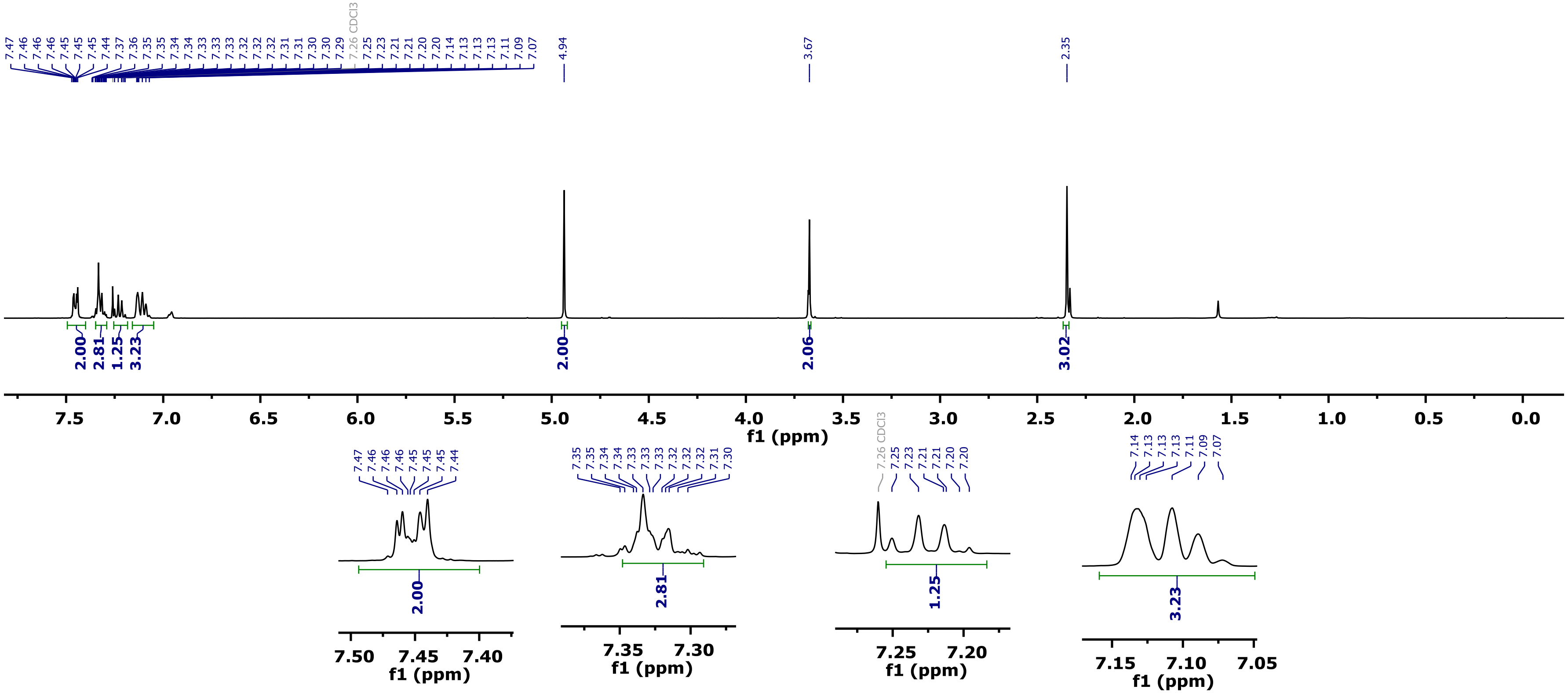

$^{13}C\{H\}$ NMR ($CDCl_3$, 100.6 MHz)

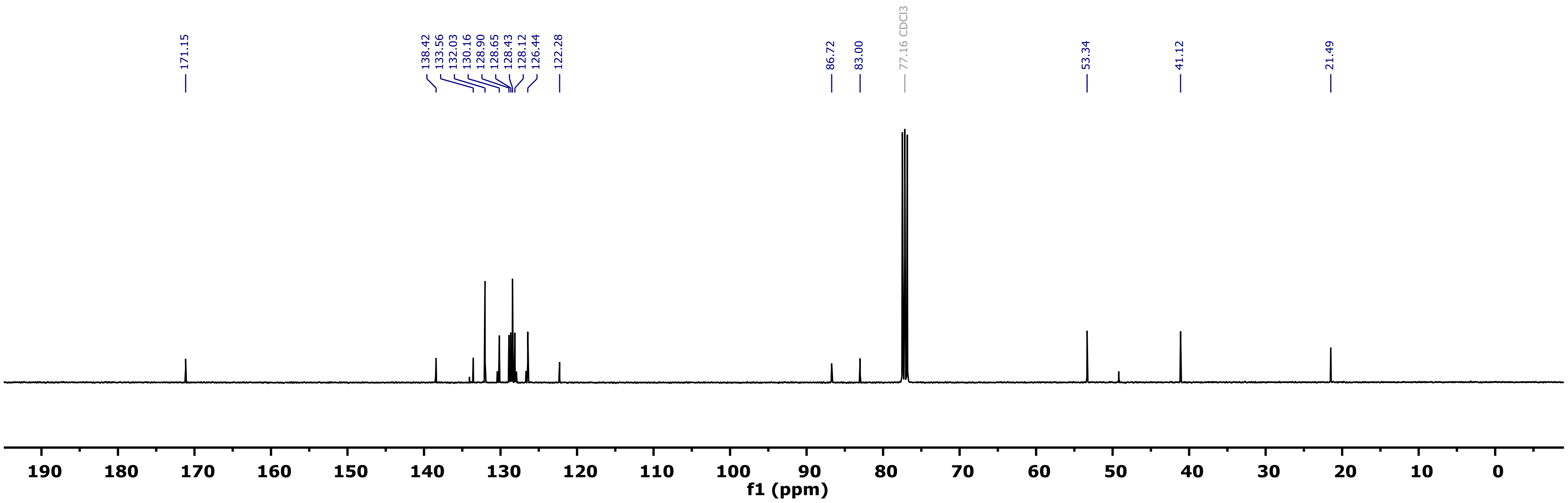

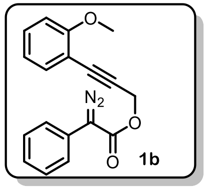

1b

[1] H NMR (400.13 MHz, $CDCl_3$)

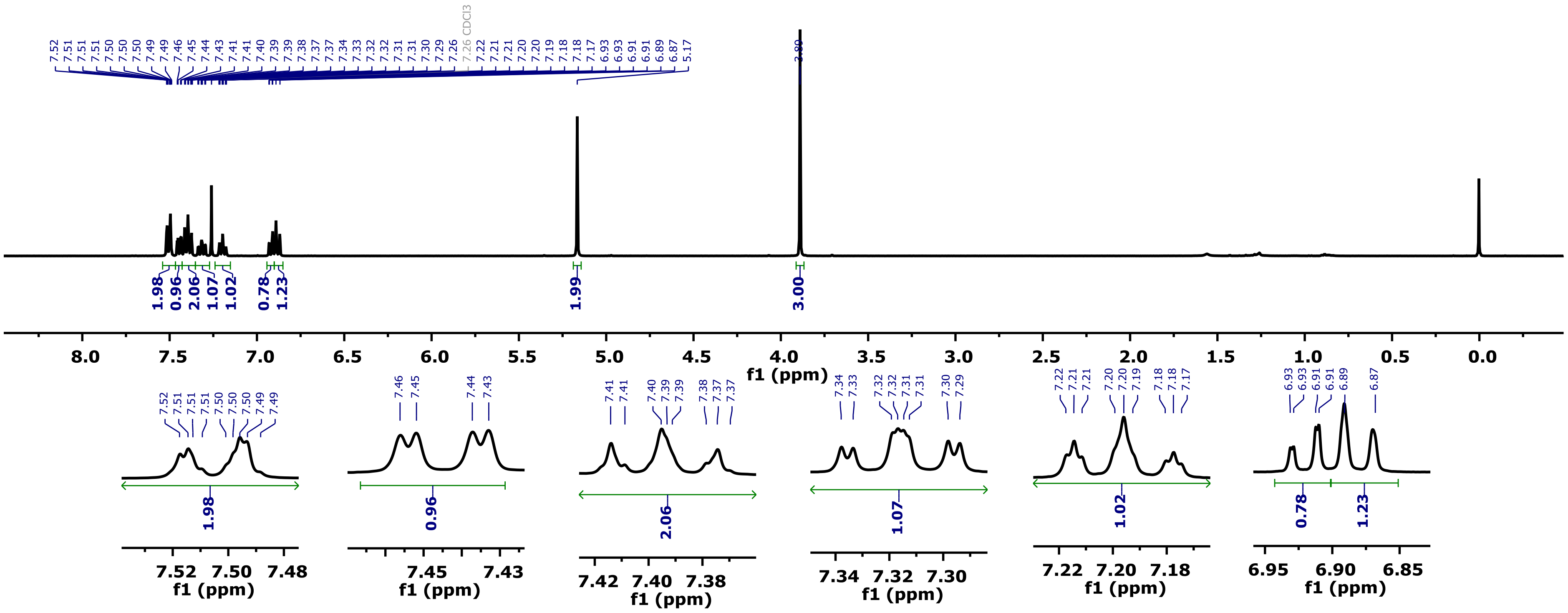

$^{13}C\{H\}$ NMR ($CDCl_3$, 100.6 MHz)

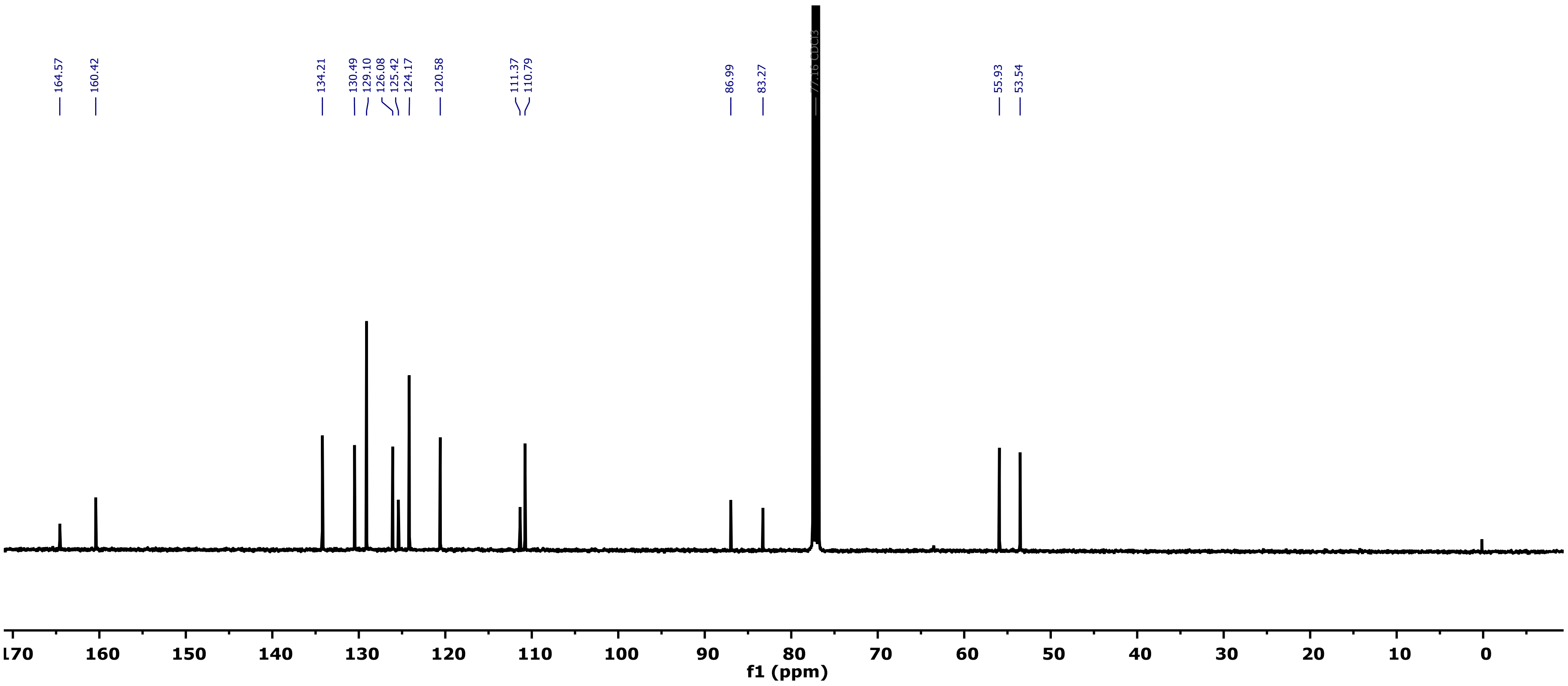

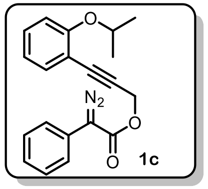

1c

^{1}H NMR (400.13 MHz, $CDCl_3$)

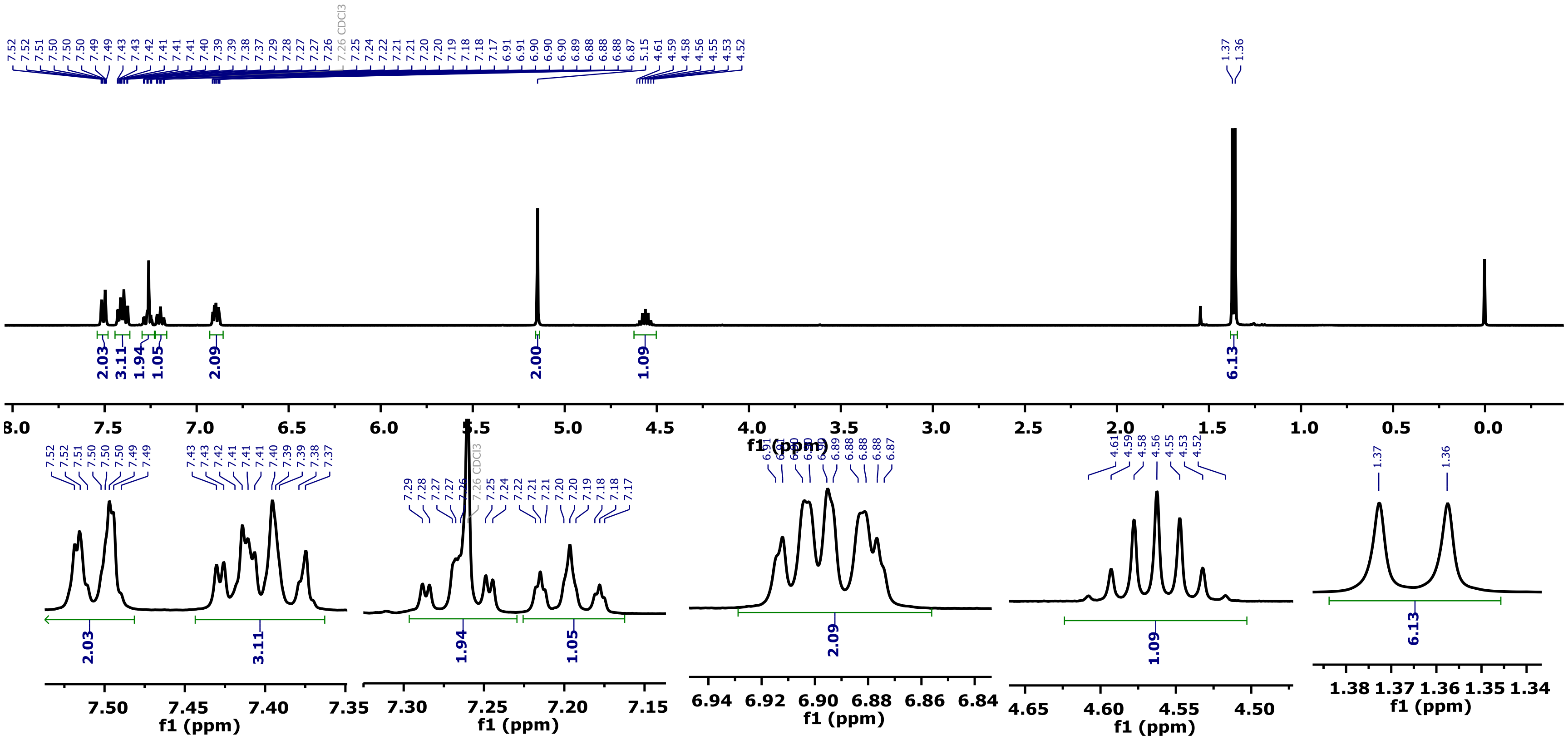

$^{13}C\{H\}$ NMR ($CDCl_3$, 100.6 MHz)

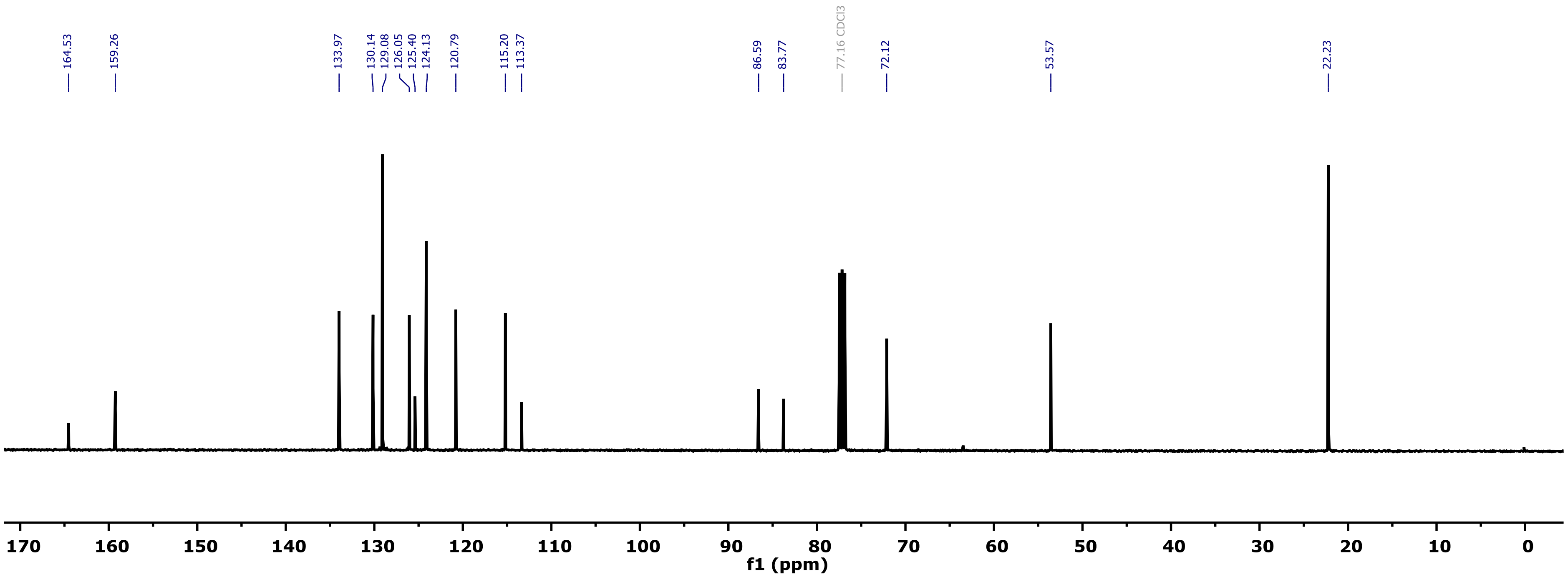

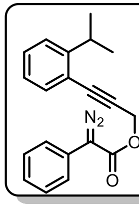

1e

^{1}H NMR (400.13 MHz, $CDCl_3$)

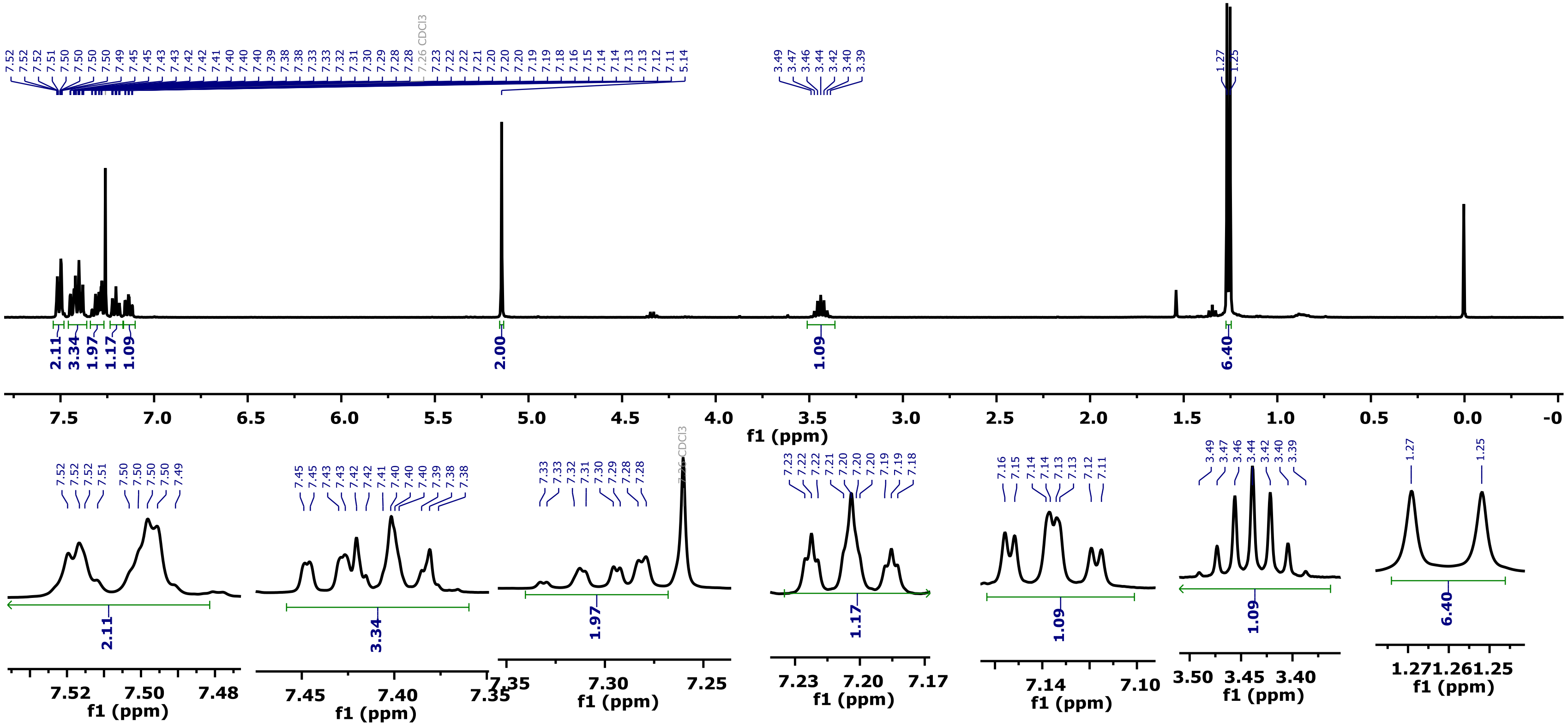

$^{13}C\{H\}$ NMR ($CDCl_3$, 100.6 MHz)

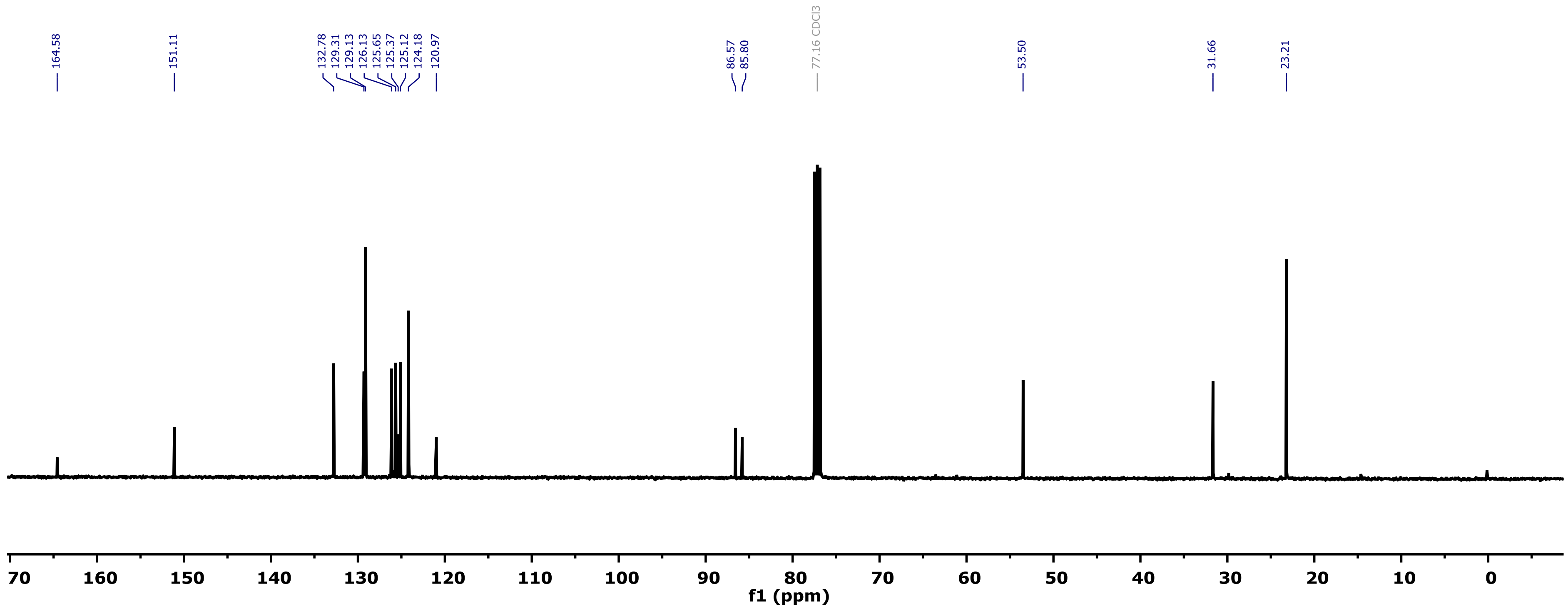

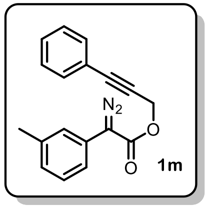

^1H NMR (400.13 MHz, $CDCl_3$)

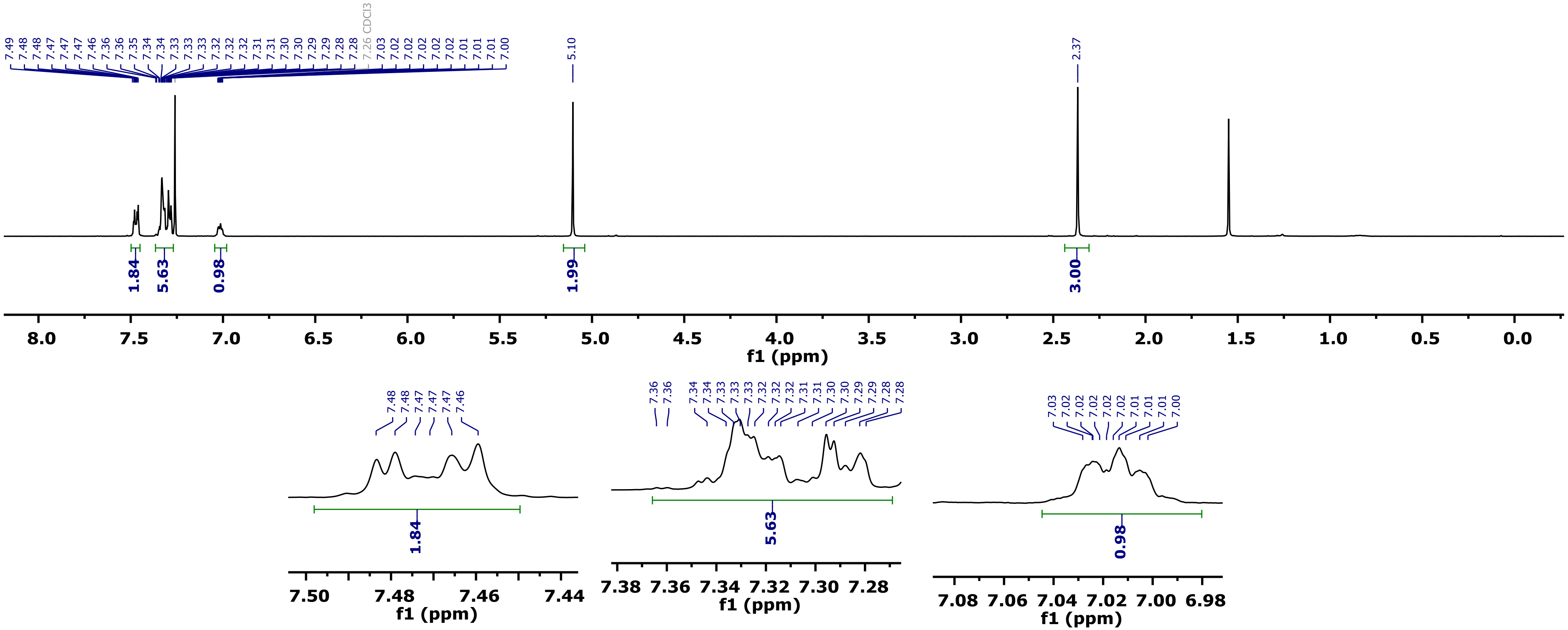

$^{13}C\{H\}$ NMR ($CDCl_3$, 100.6 MHz)

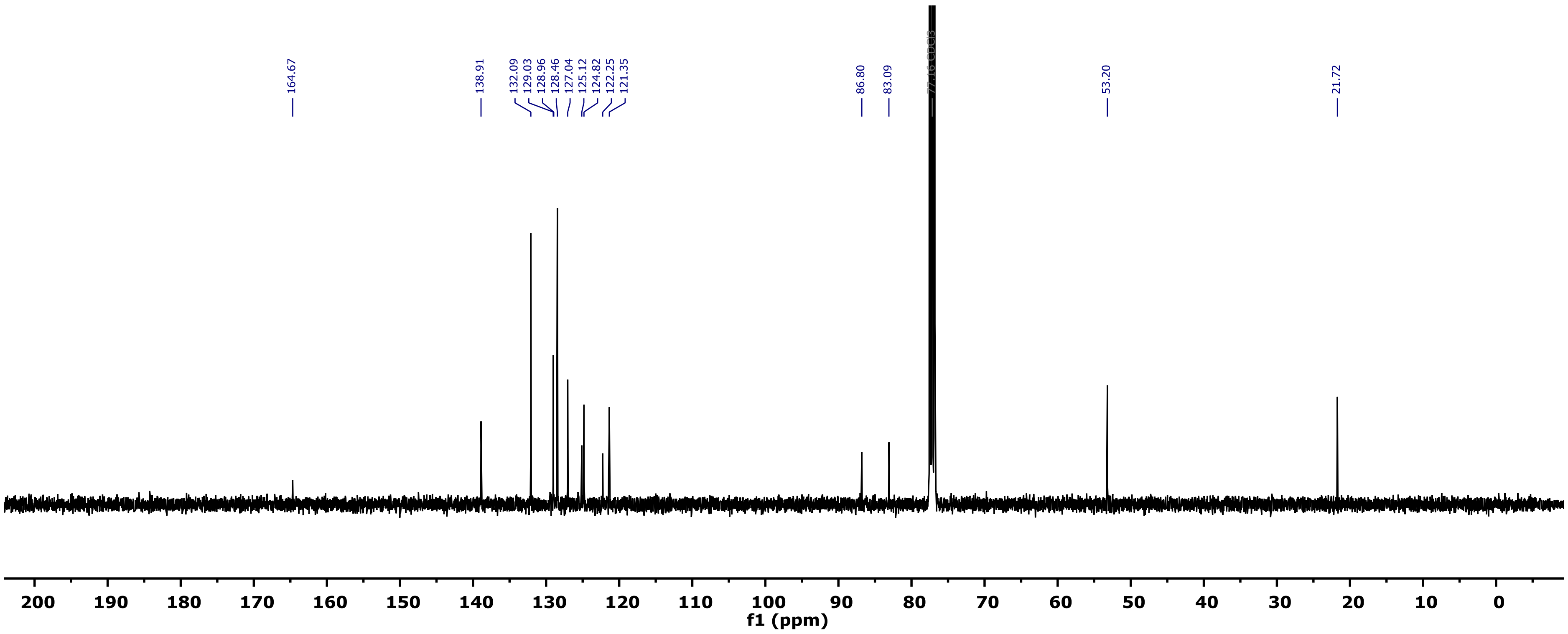

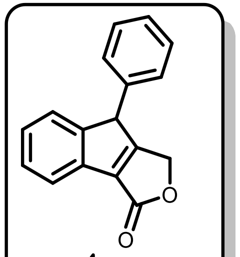

4a

^{1}H NMR (400.13 MHz, $CDCl_3$)

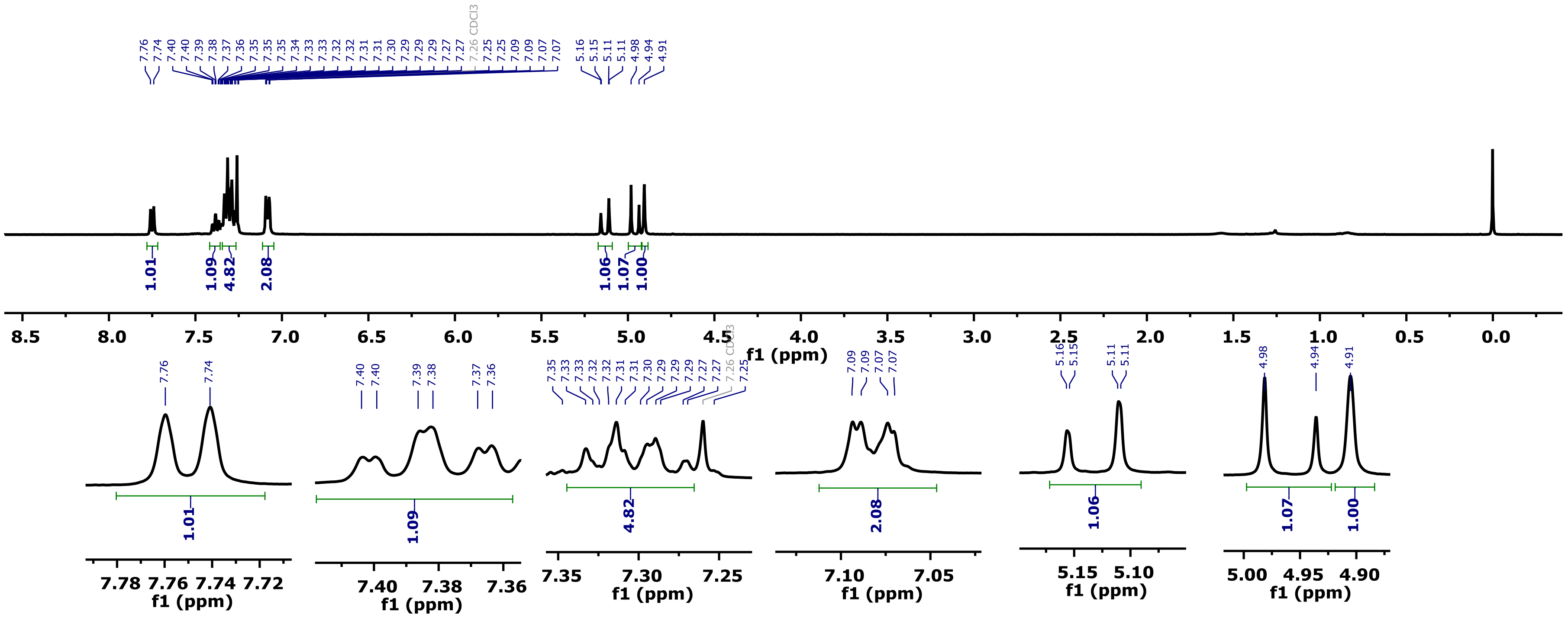

^{13}C{H} NMR ($CDCl_3$, 100.6 MHz)

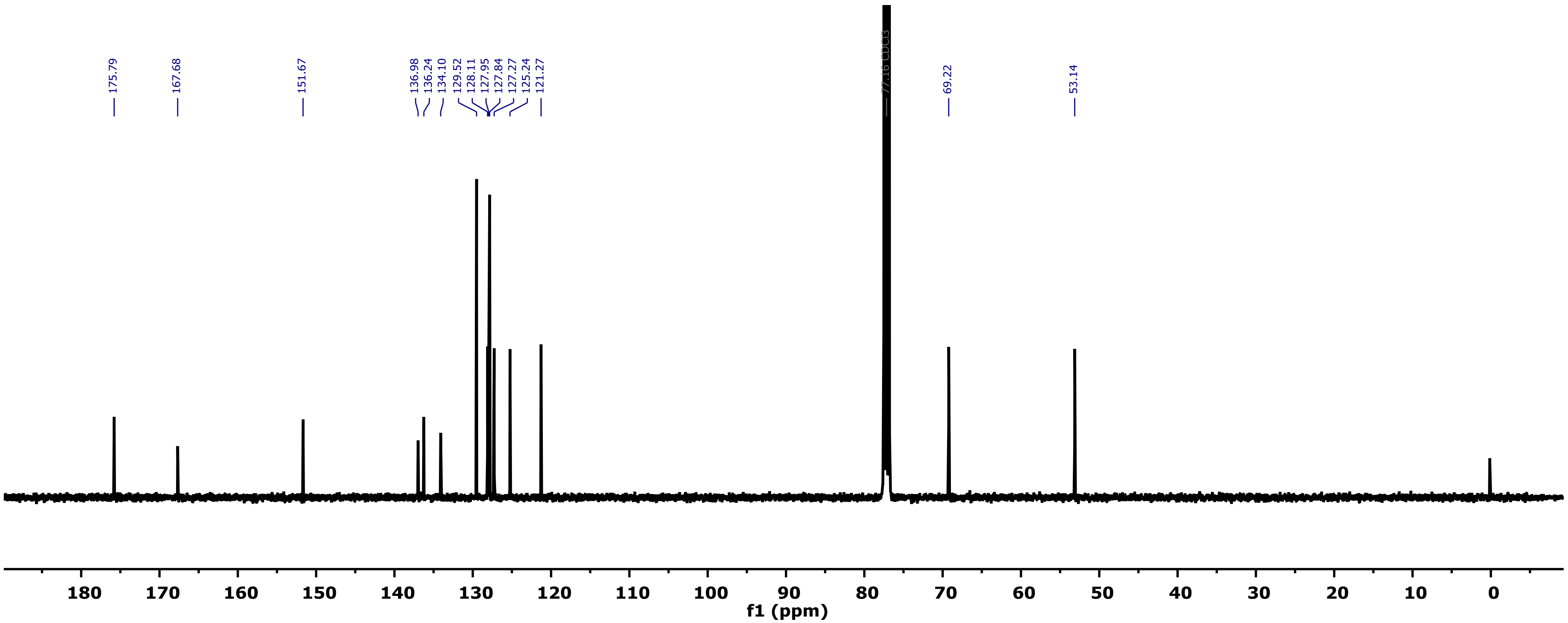

2D NMR HSQC ($CDCl_3$)

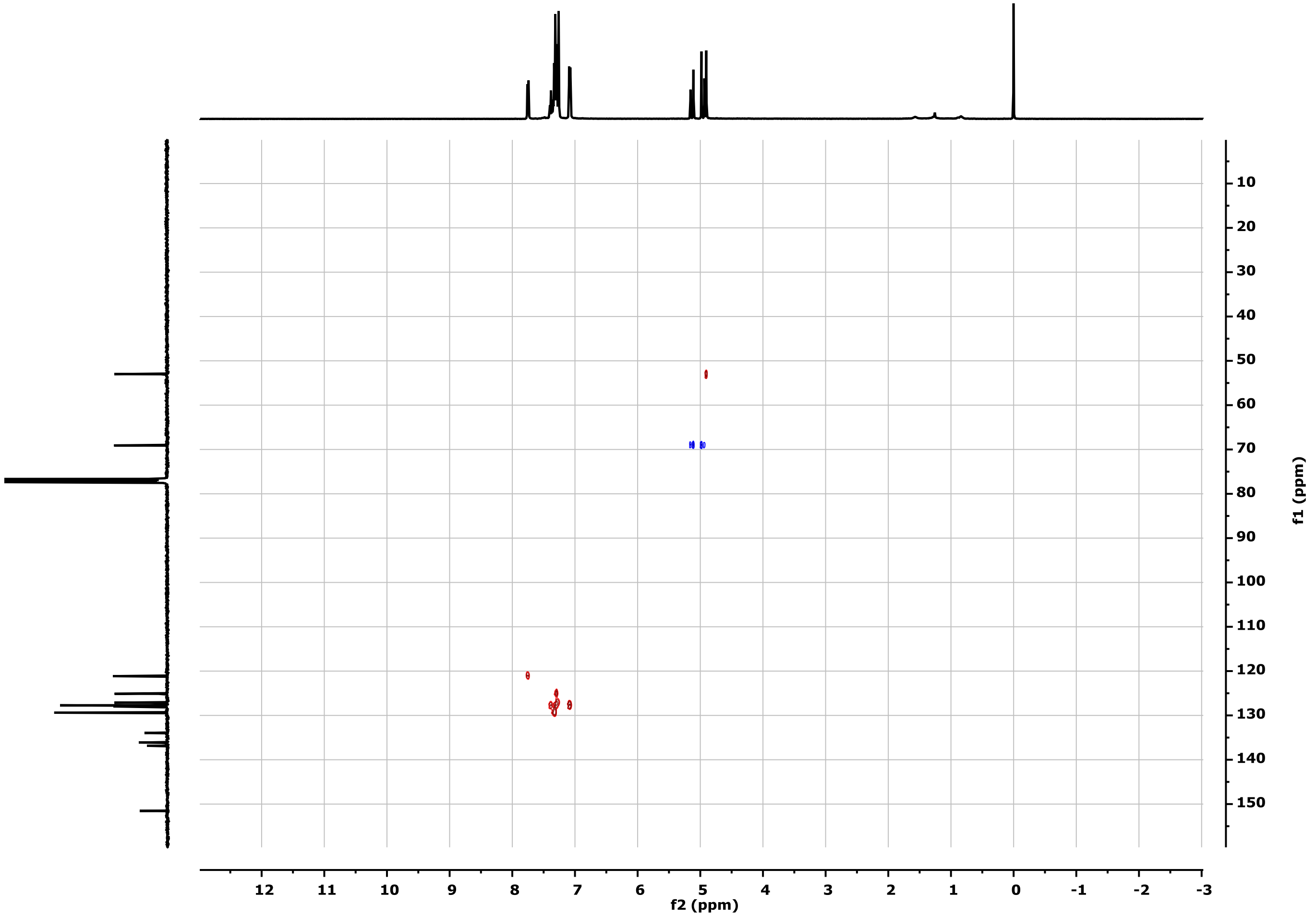

2D NMR COSY ($CDCl_3$)

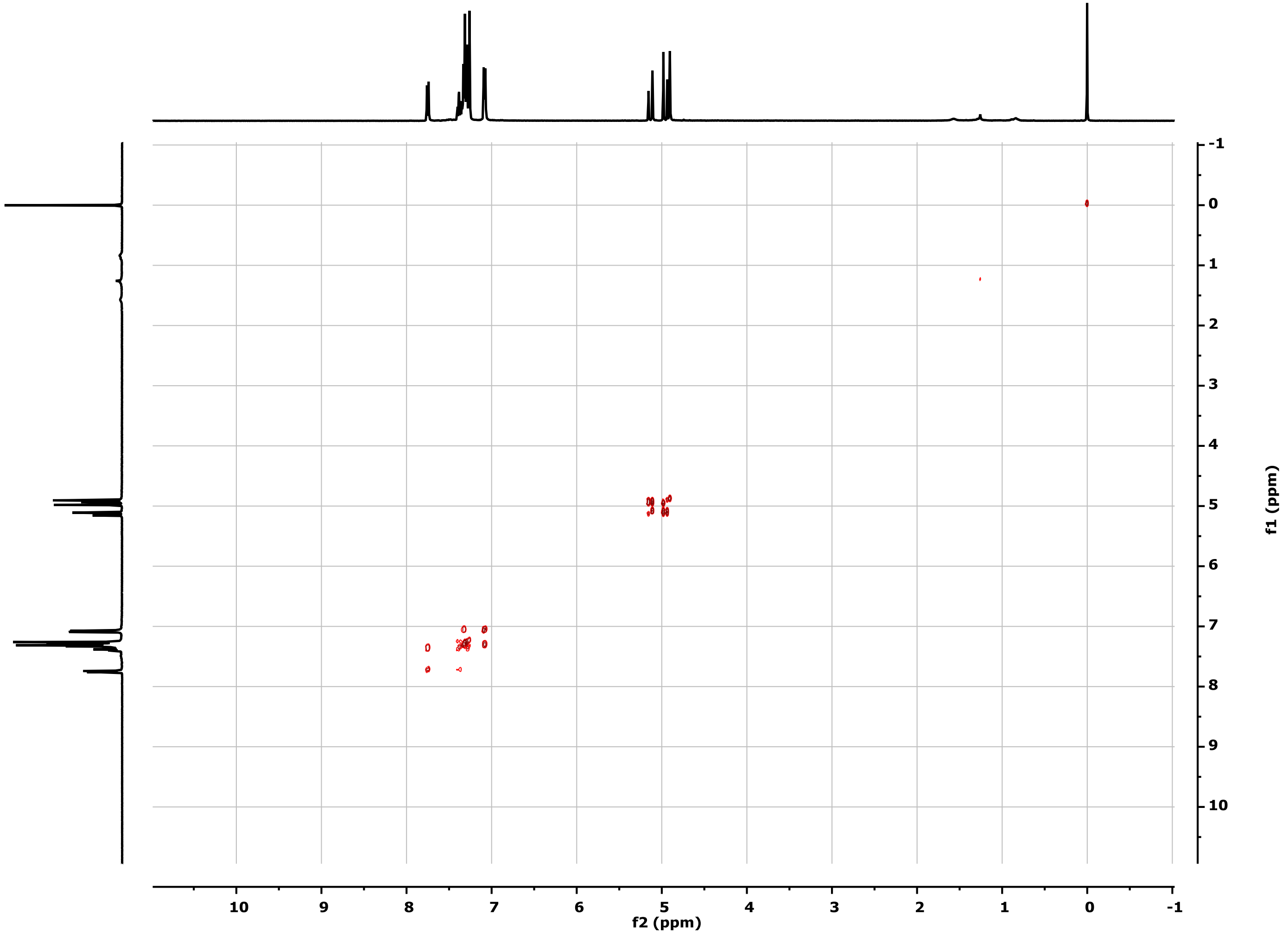

2D NMR HMBC ($CDCl_3$)

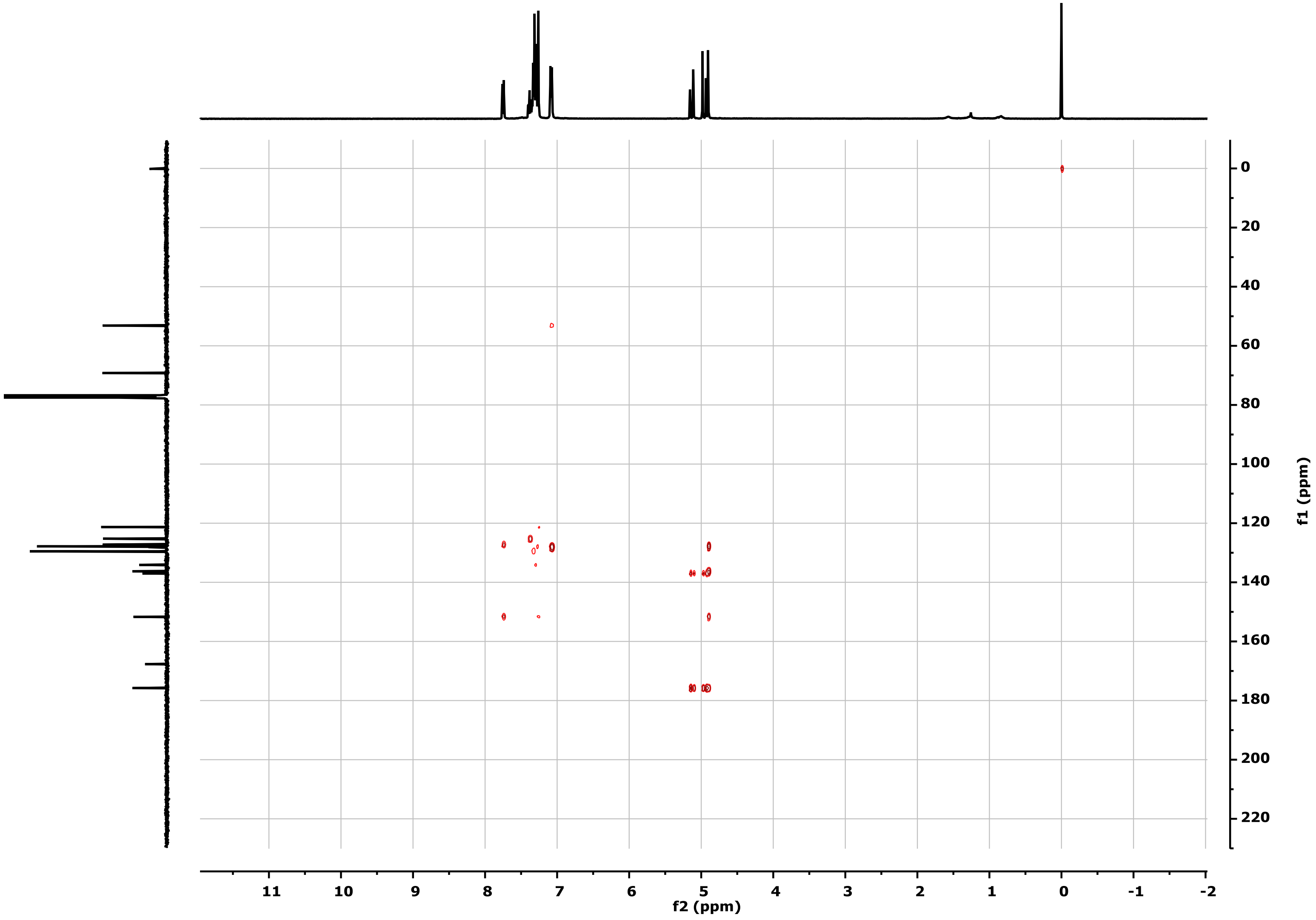

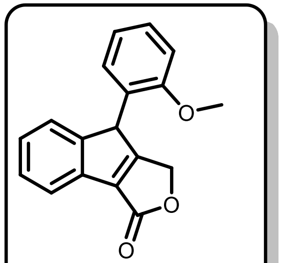

4b

^1H NMR (400.13 MHz, $CDCl_3$)

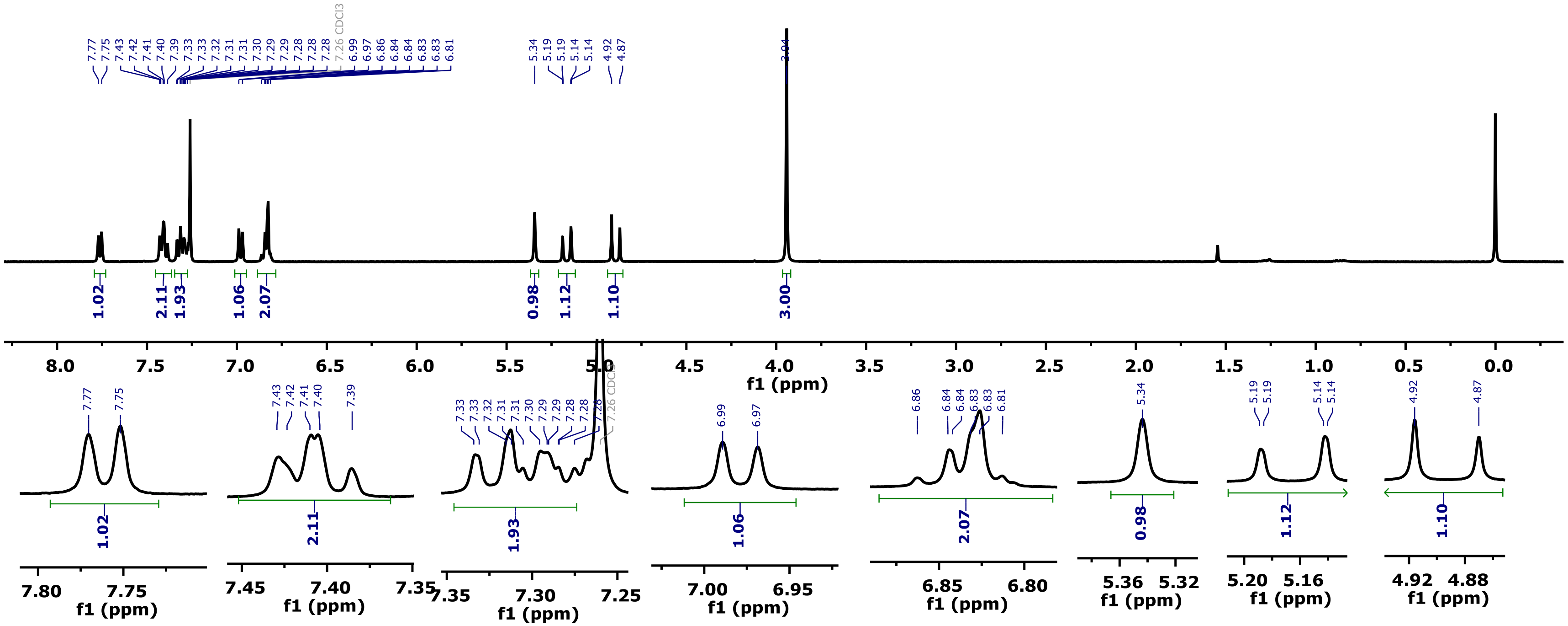

$^{13}C\{H\}$ NMR ($CDCl_3$, 100.6 MHz)

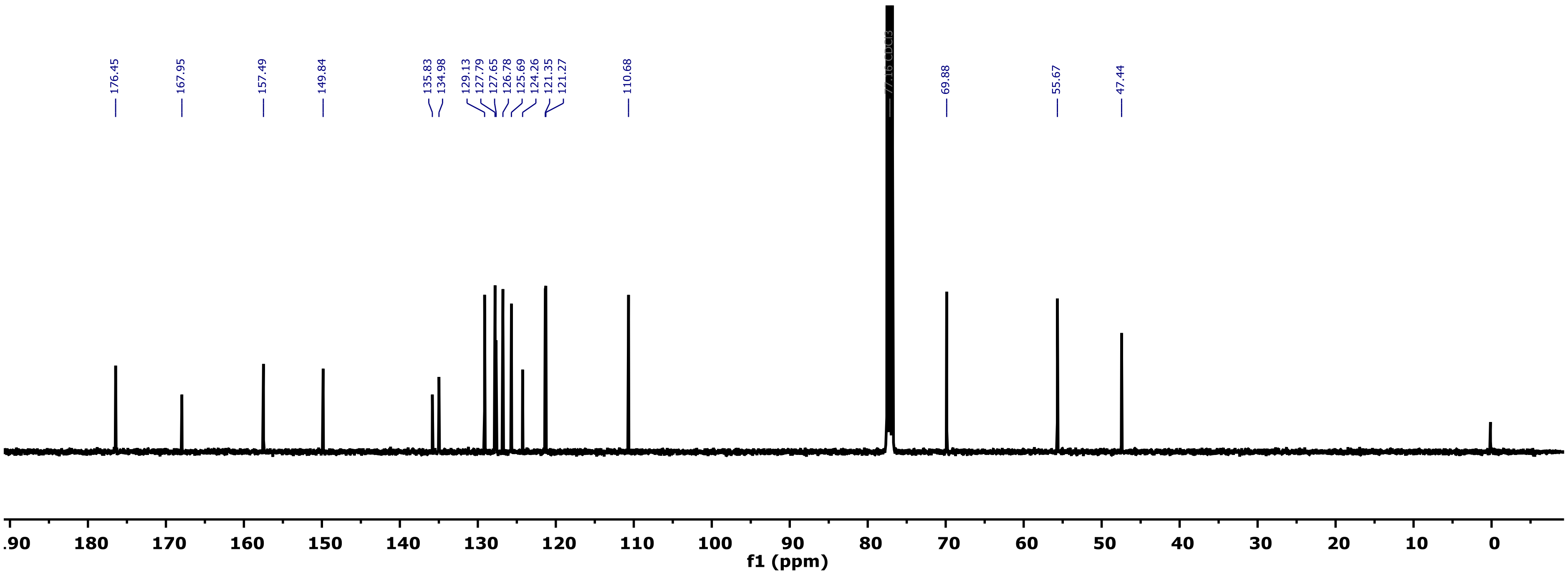

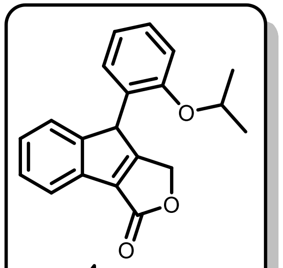

4c

^{1}H NMR (400.13 MHz, $DMSO_{d6}$, 65ºC)

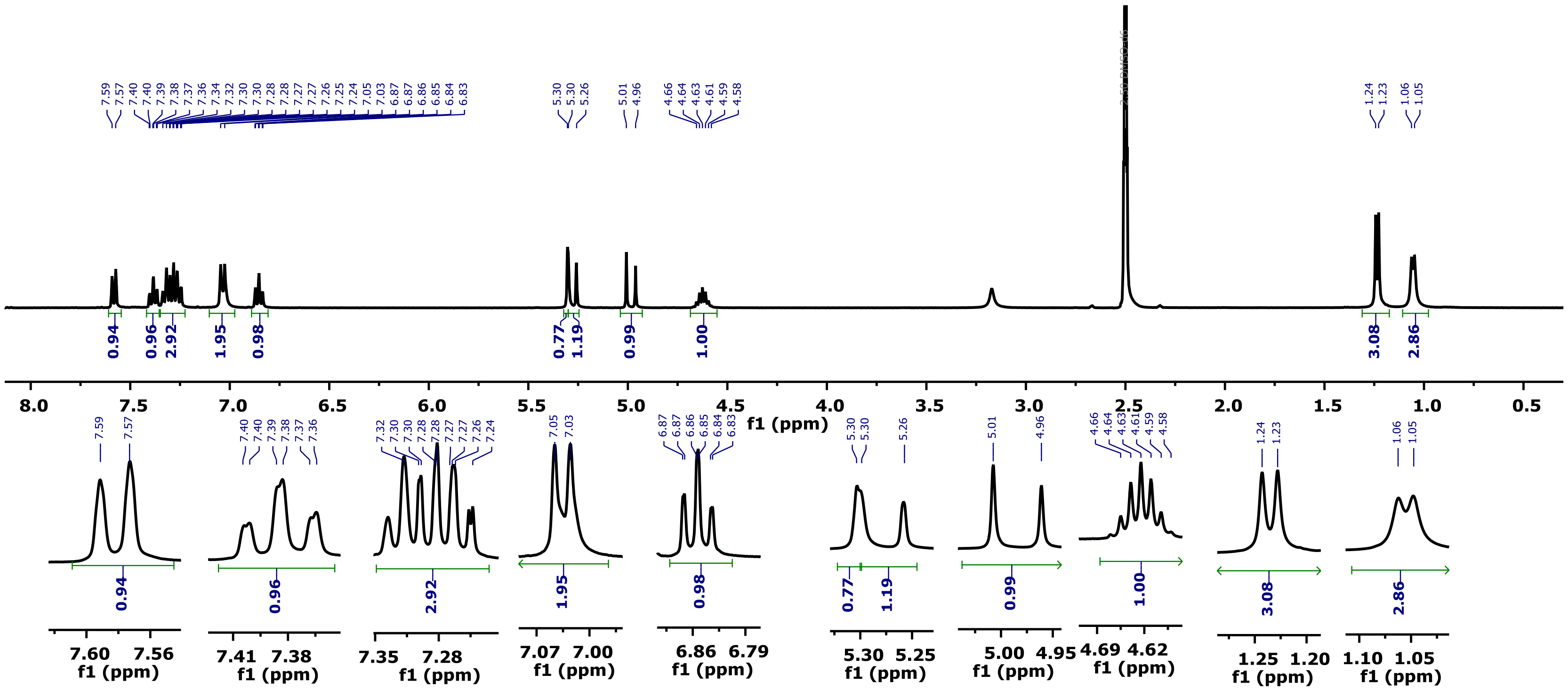

$^{13}C\{H\}$ NMR ($CDCl_3$, $DMSO_{d6}$, 65ºC)

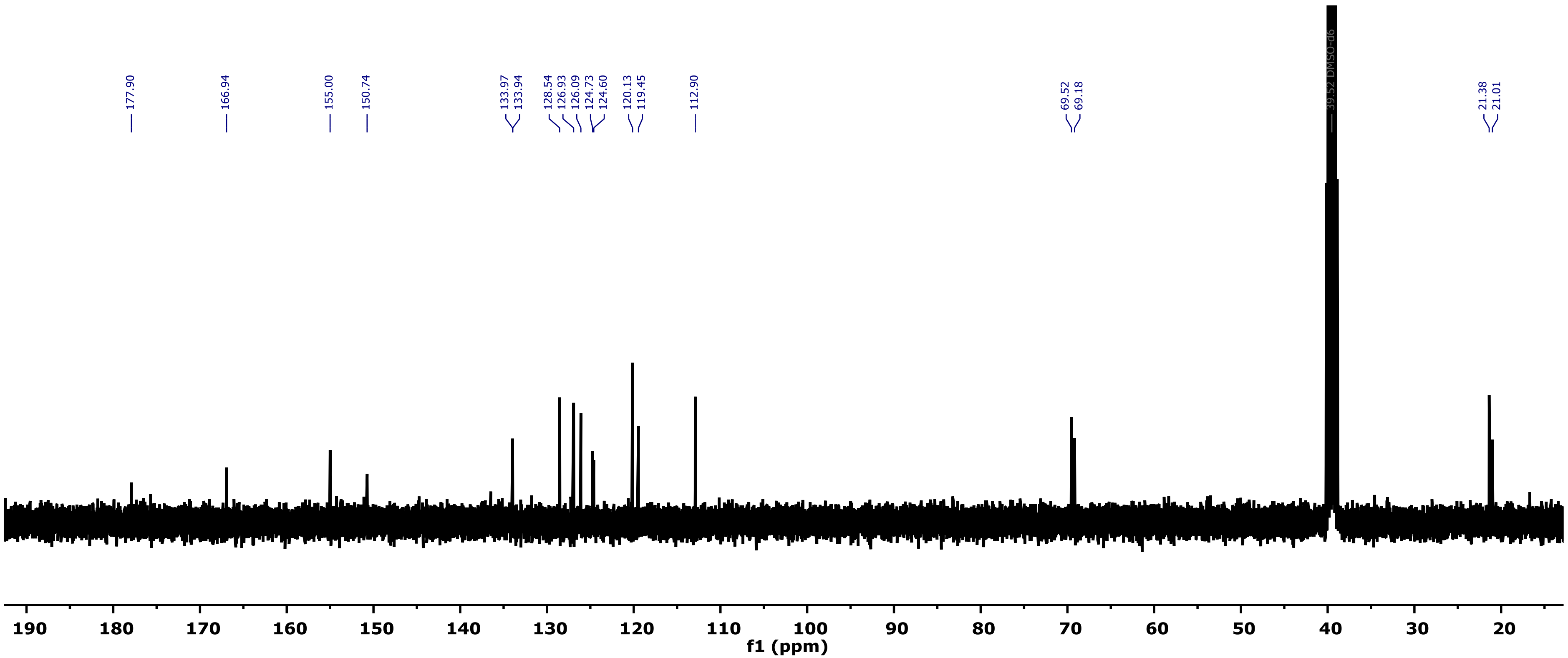

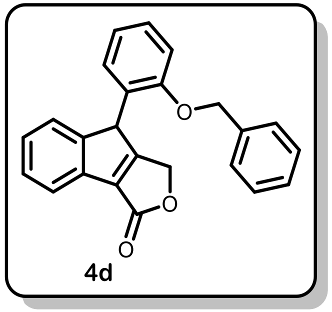

4d

^{1}H NMR (400.13 MHz, $CDCl_3$)

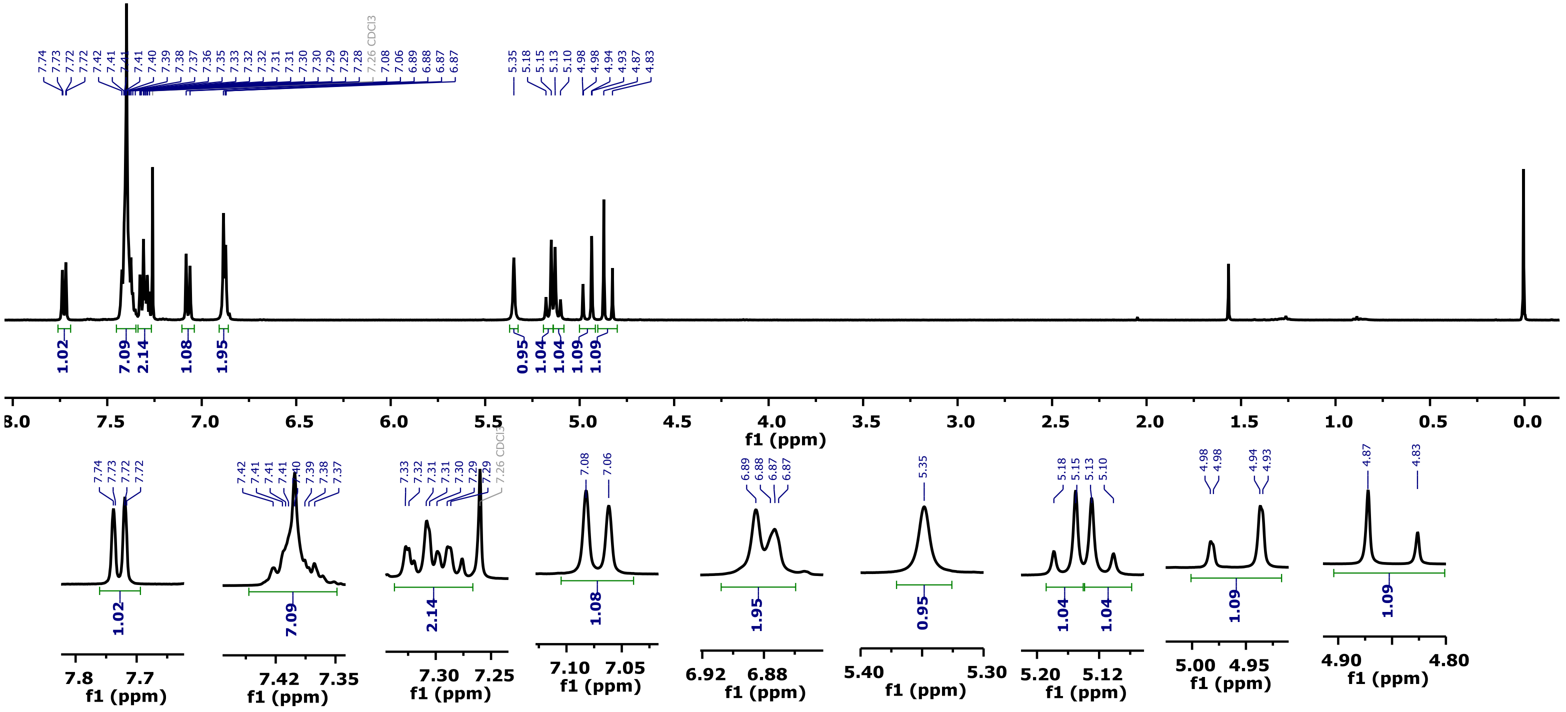

$^{13}C\{H\}$ NMR ($CDCl_3$, 100.6 MHz)

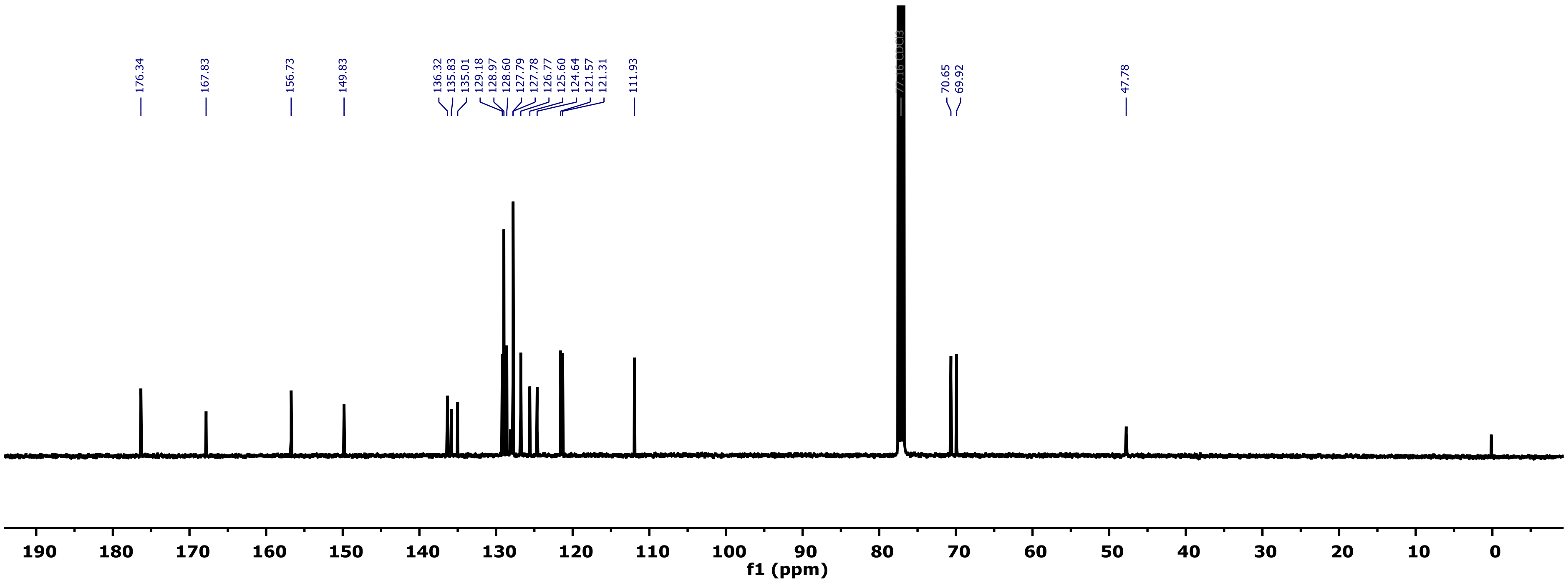

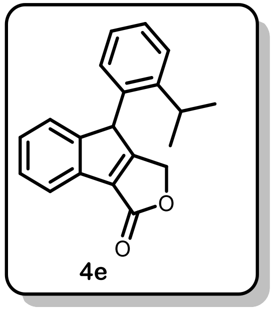

4e

^{1}H NMR (400.13 MHz, $CDCl_3$)

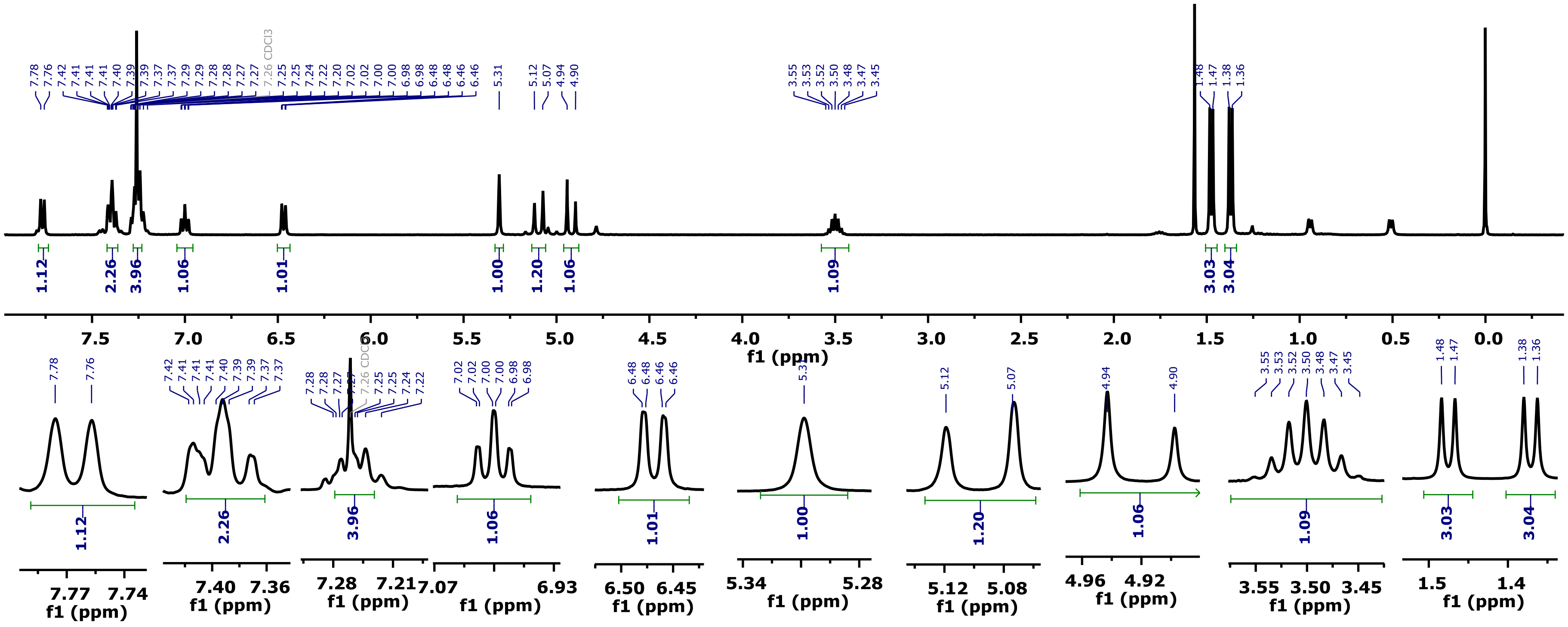

$^{13}C\{H\}$ NMR ($CDCl_3$, 100.6 MHz)

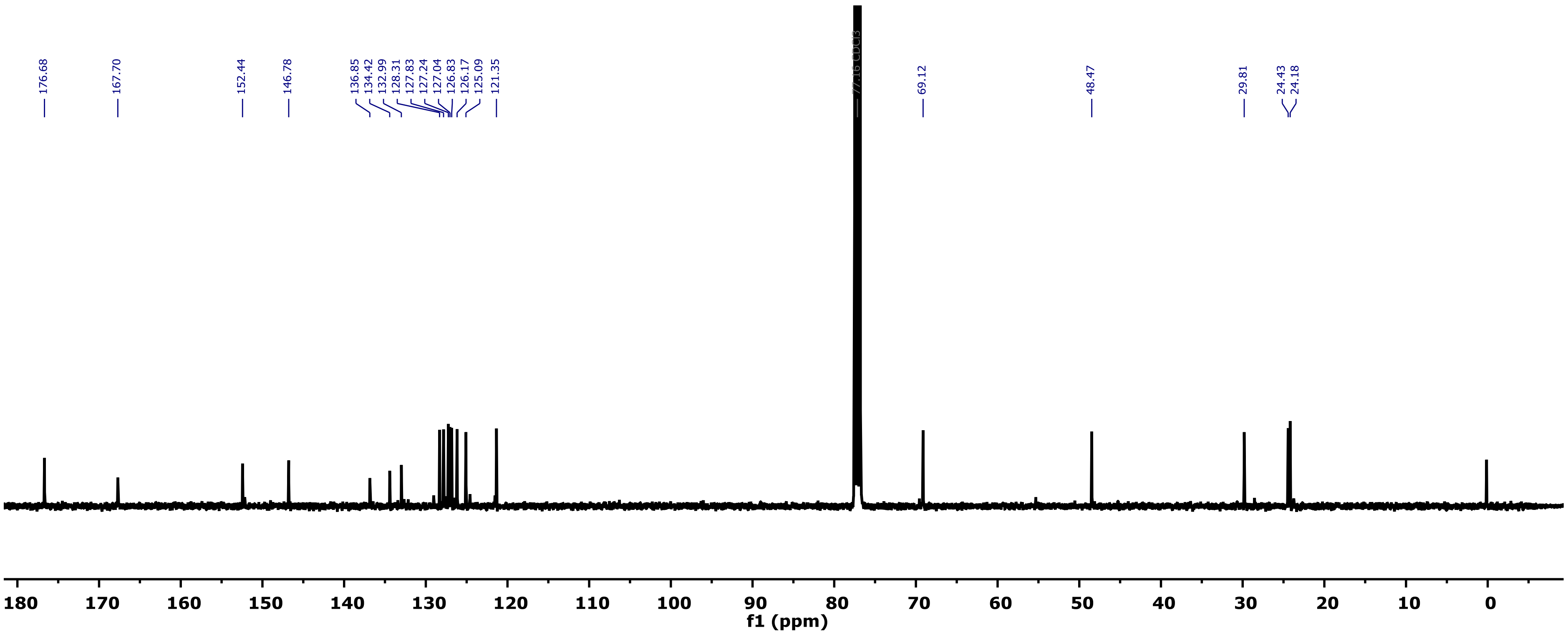

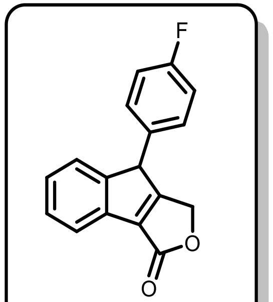

4f

^{1}H NMR (400.13 MHz, $CDCl_3$):

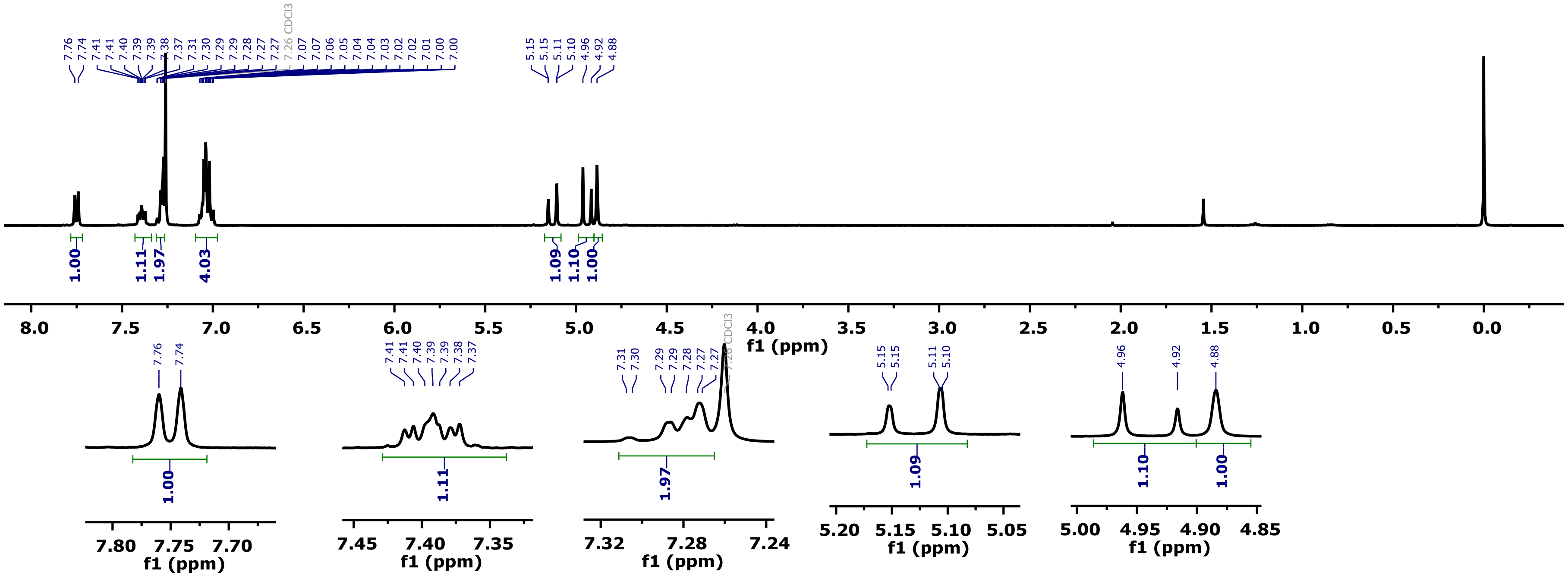

$^{13}C\{H\}$ NMR ($CDCl_3$, 100.6 MHz):

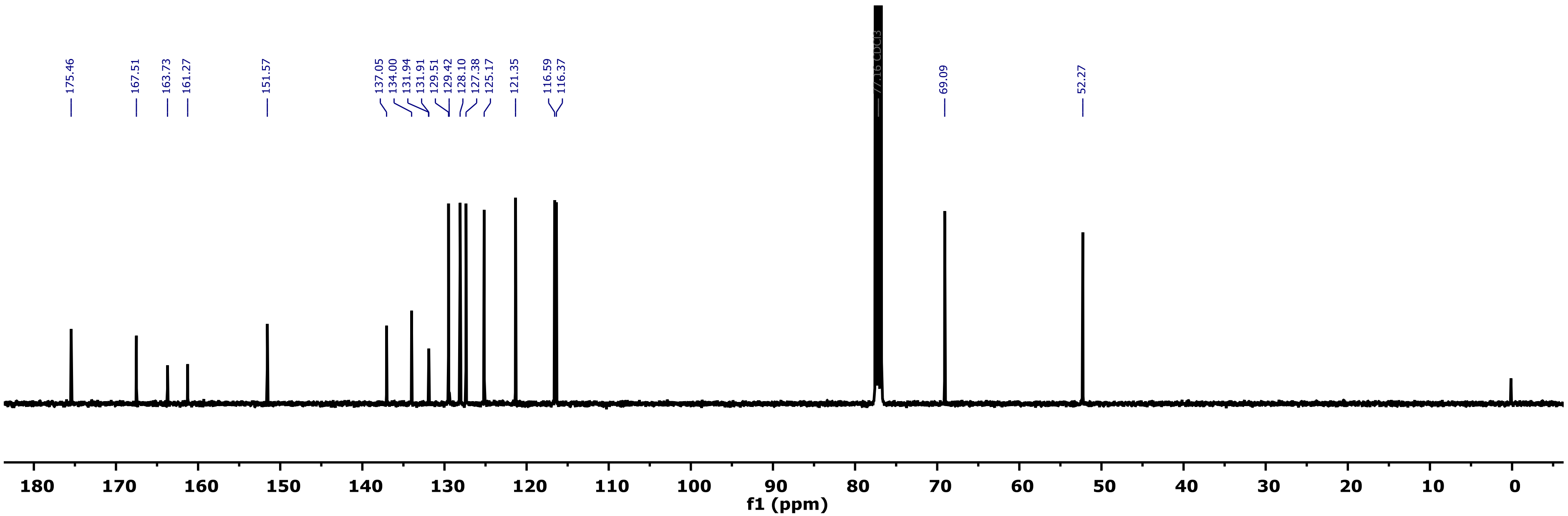

^{19}F NMR ($CDCl_3$, 376 MHz):

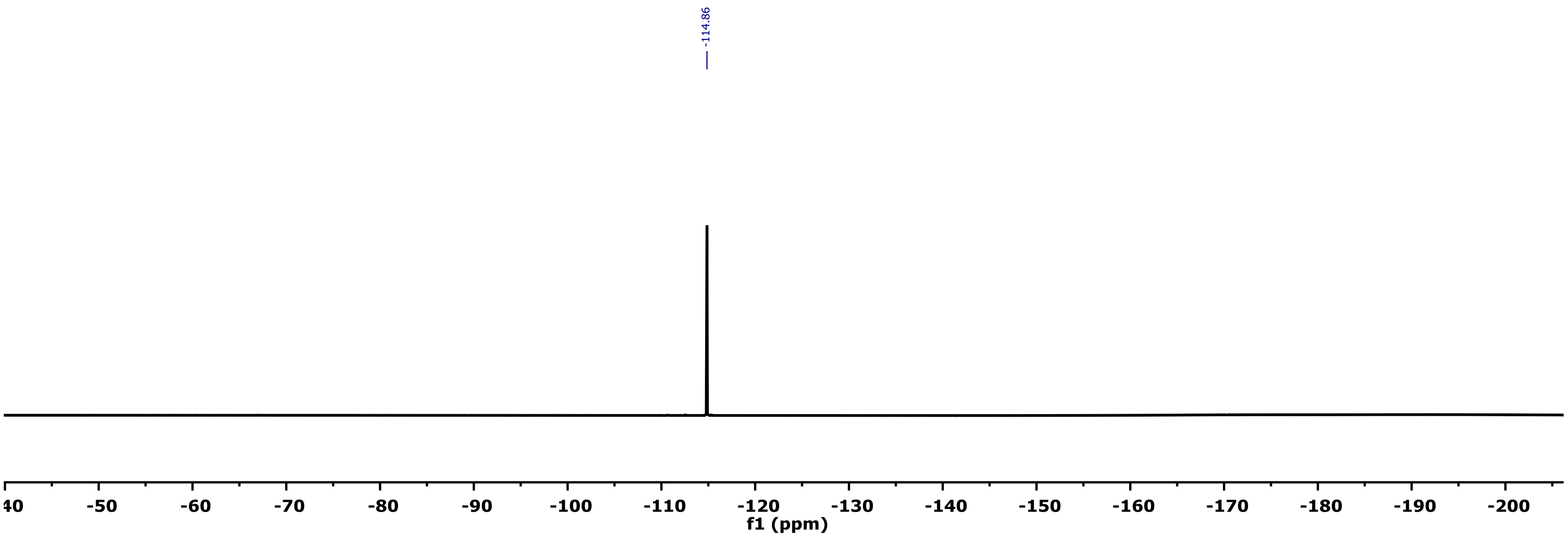

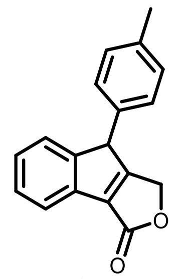

4g

^{1}H NMR (400.13 MHz, $CDCl_3$)

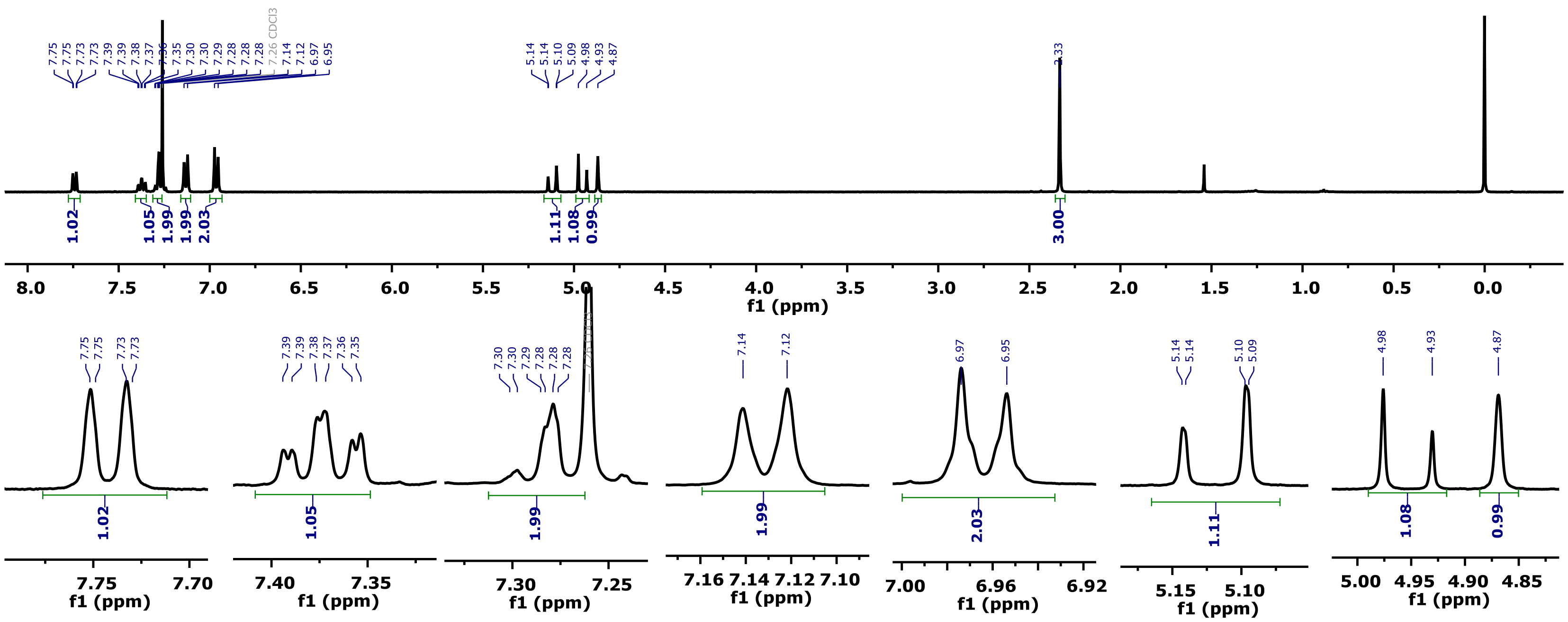

$^{13}C\{H\}$ NMR ($CDCl_3$, 100.6 MHz)

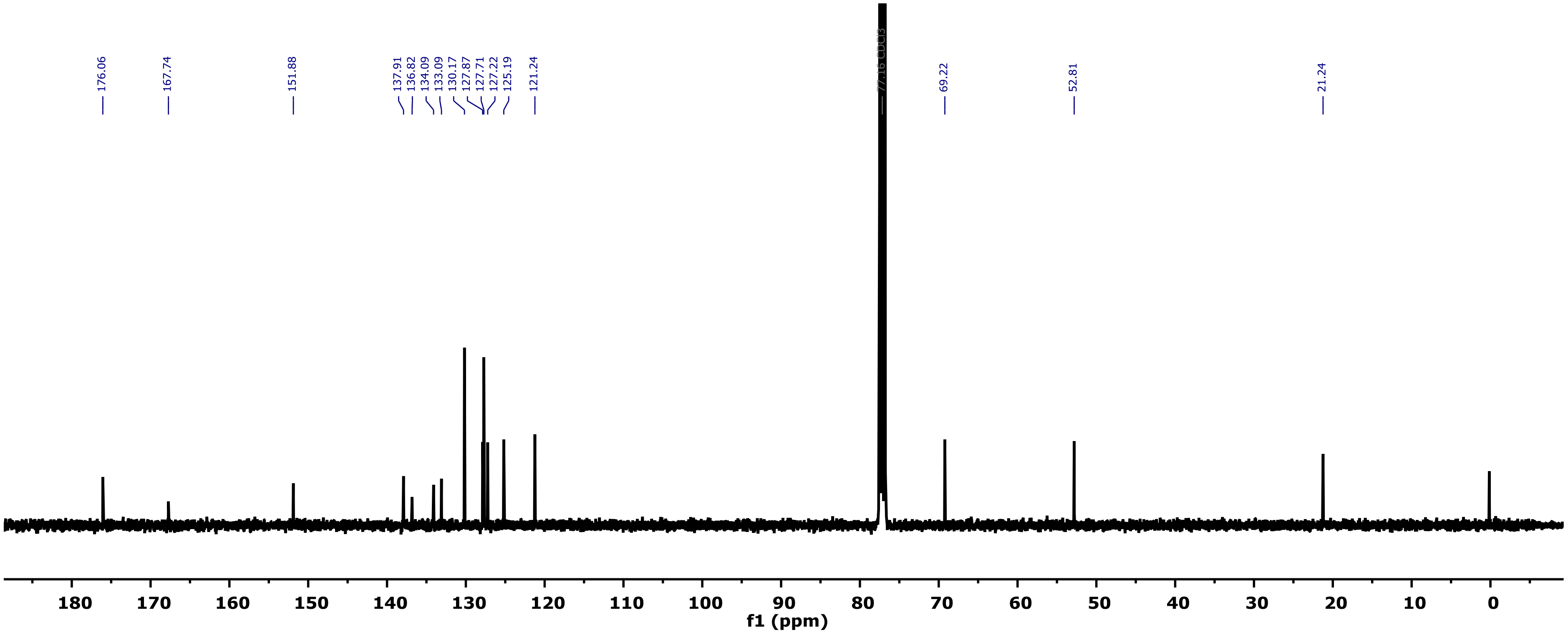

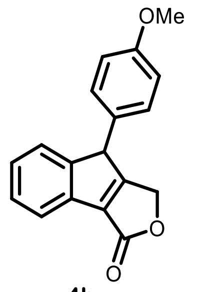

4h

^{1}H NMR (400.13 MHz, CDCl$_3$)

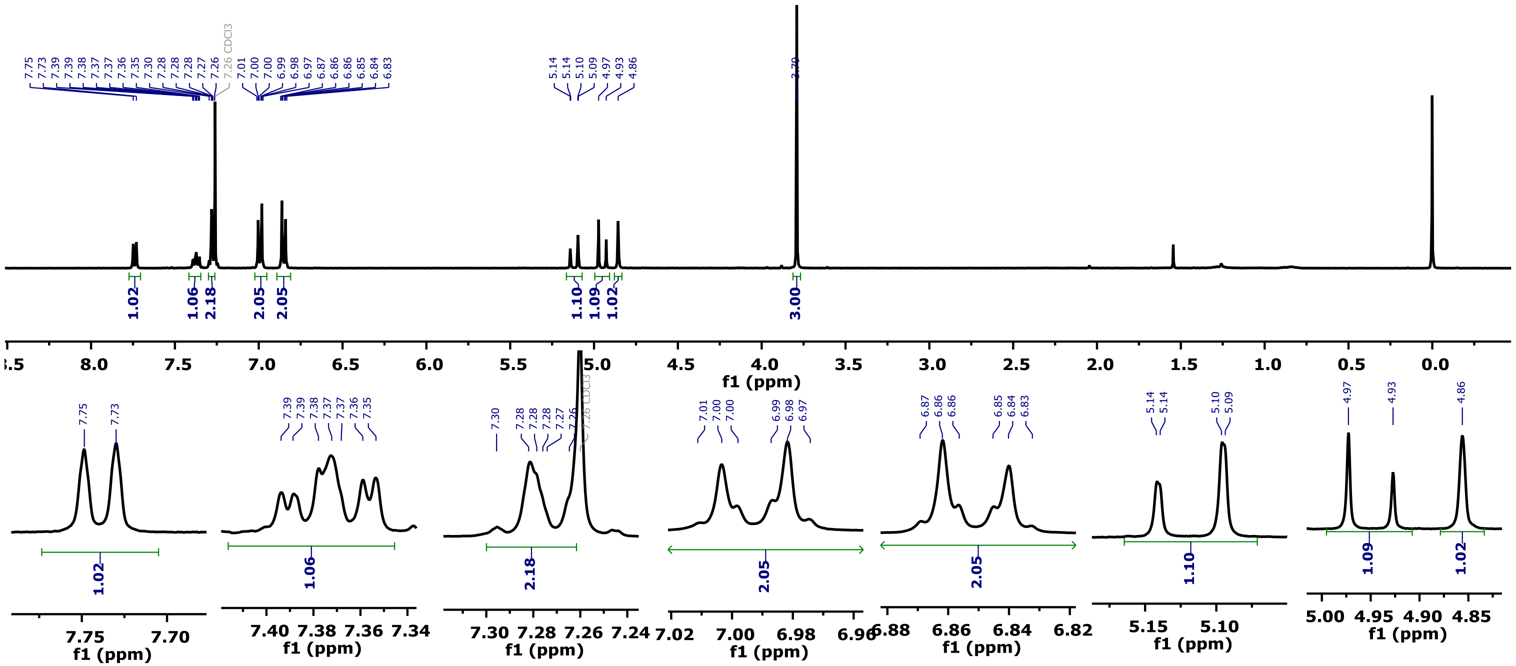

$^{13}C\{H\}$ NMR ($CDCl_3$, 100.6 MHz)

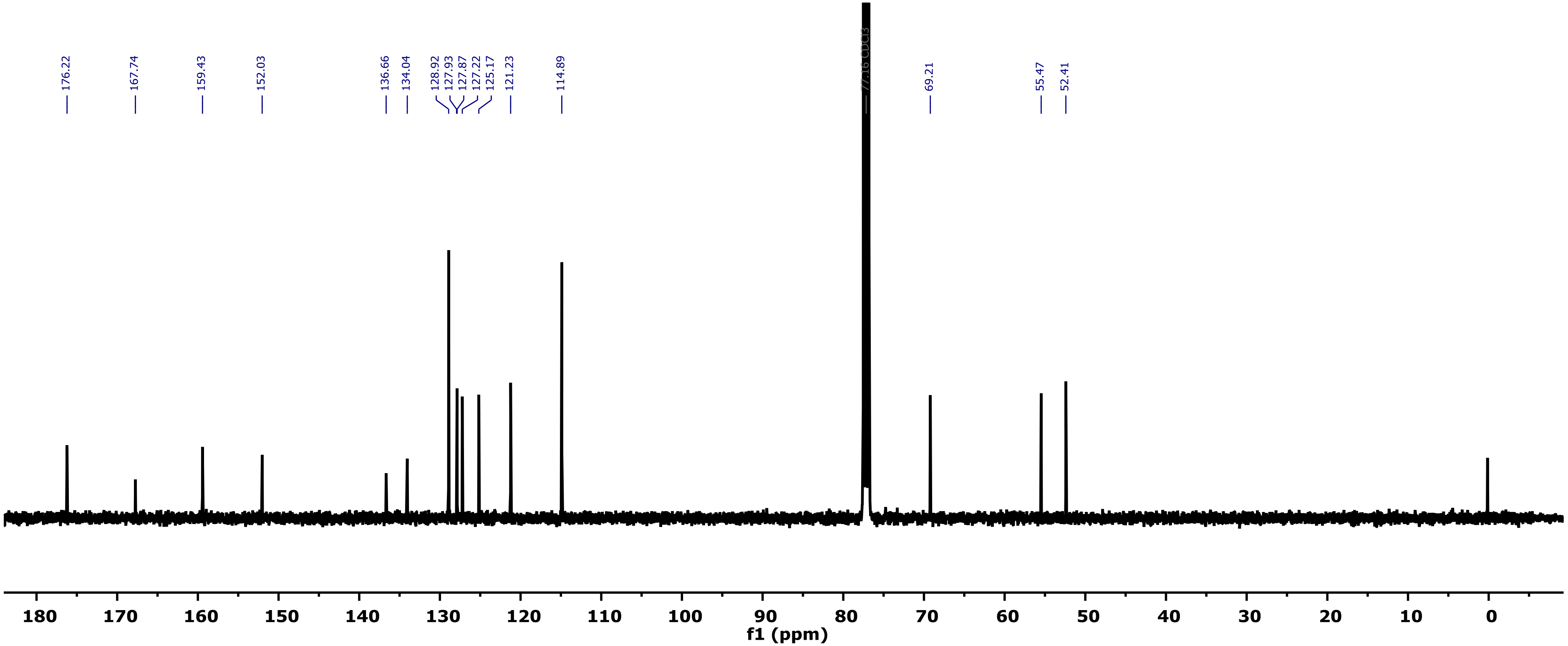

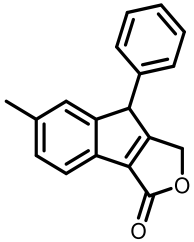

4i

^{1}H NMR (400.13 MHz, $CDCl_3$)

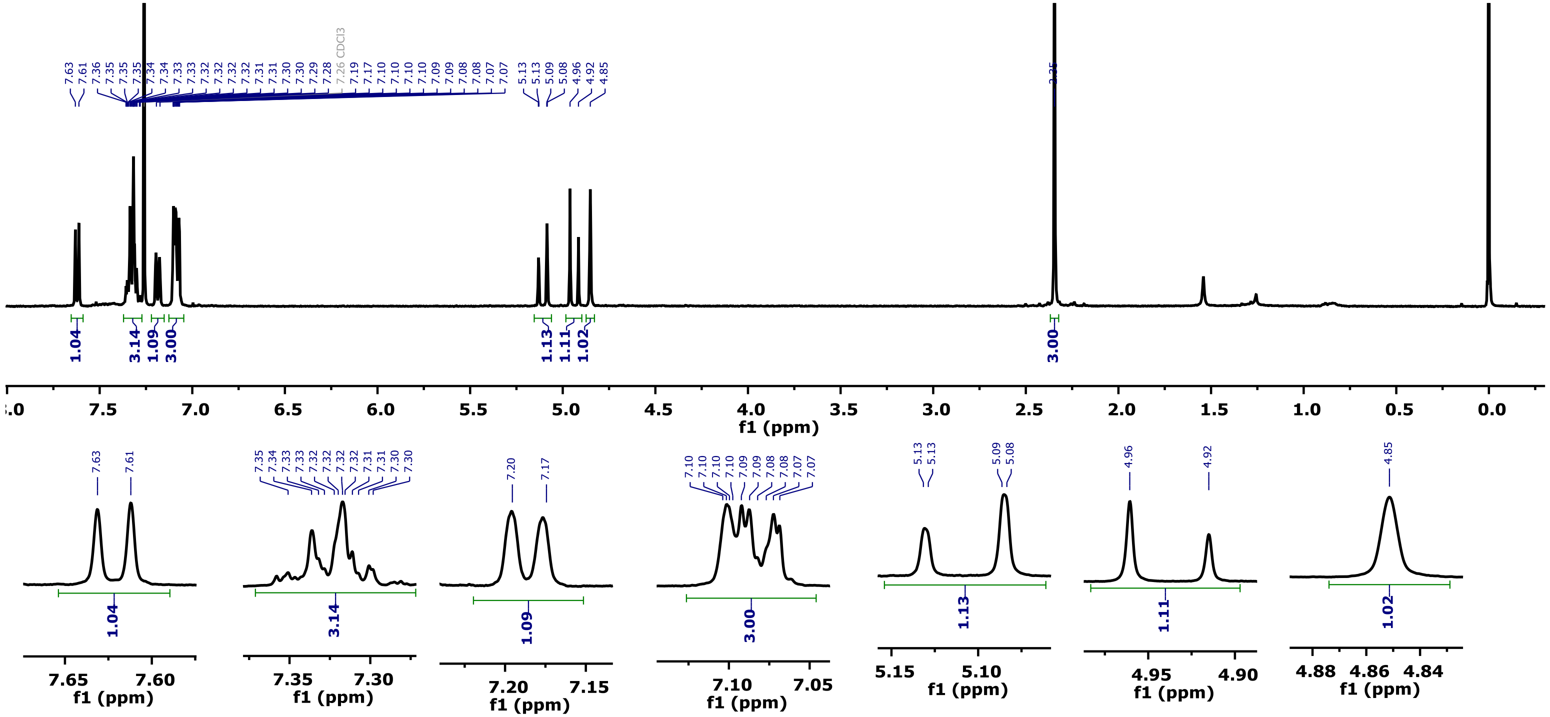

$^{13}C\{H\}$ NMR ($CDCl_3$, 100.6 MHz)

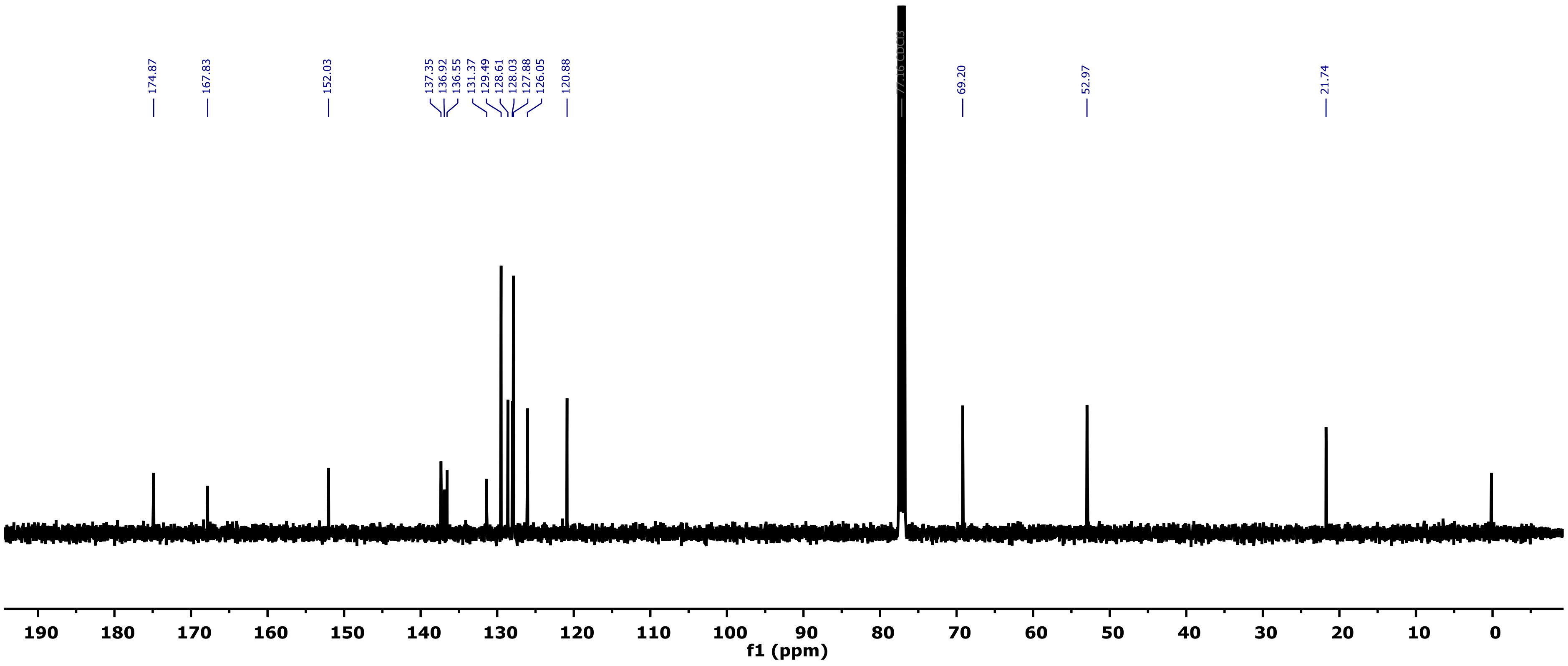

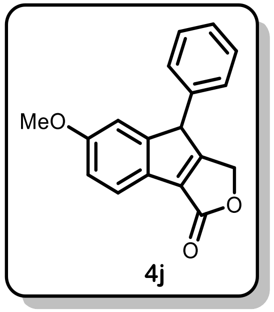

4j

^{1}H NMR (400.13 MHz, $CDCl_3$)

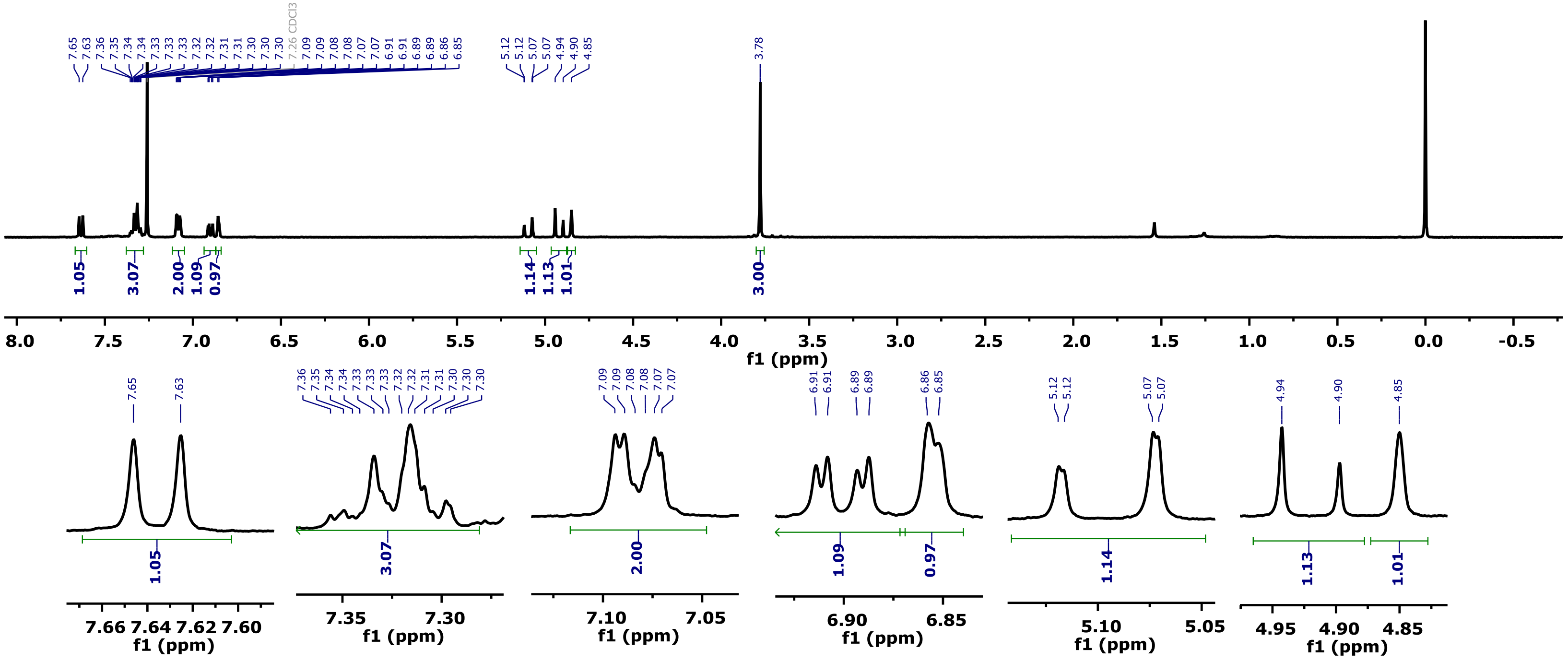

$^{13}C\{H\}$ NMR ($CDCl_3$, 100.6 MHz)

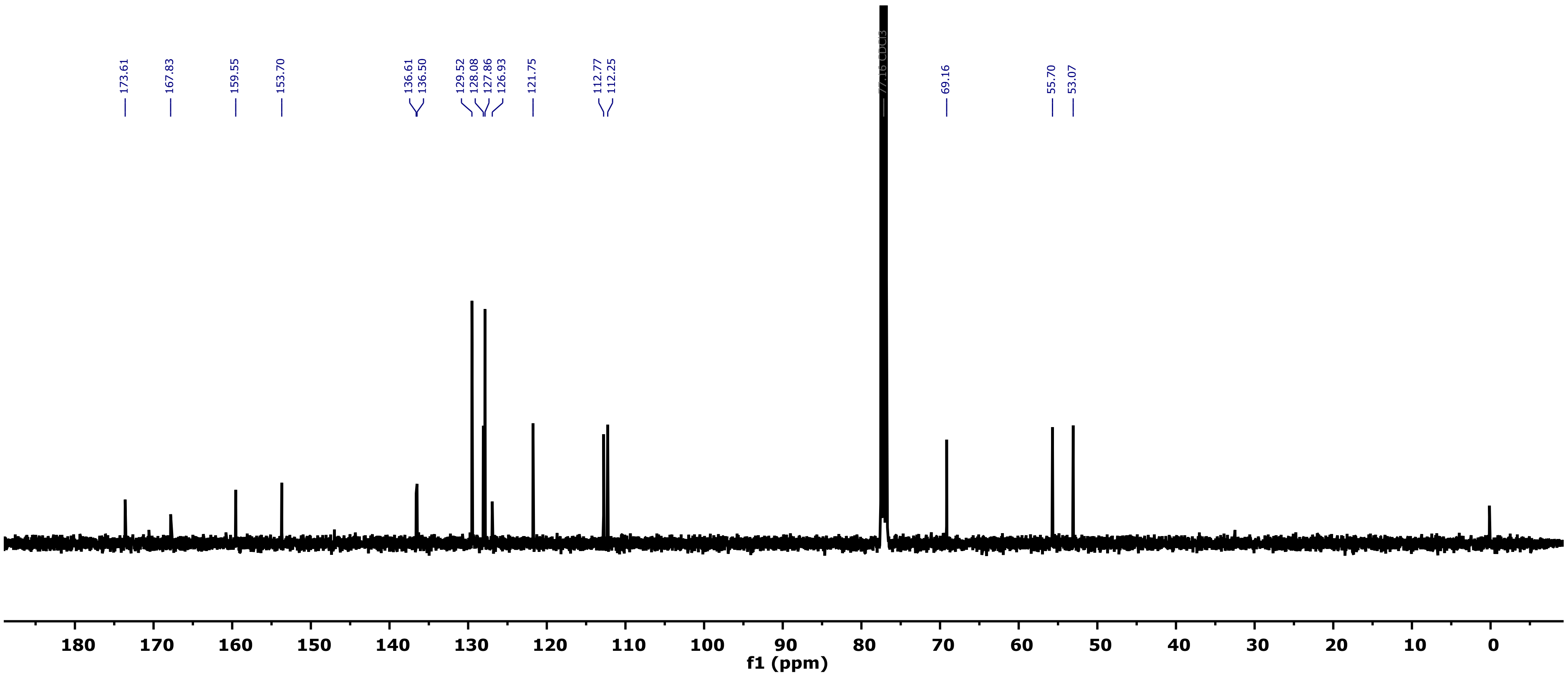

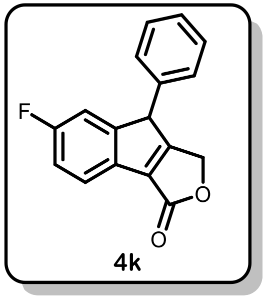

^{1}H NMR (400.13 MHz, $CDCl_3$)

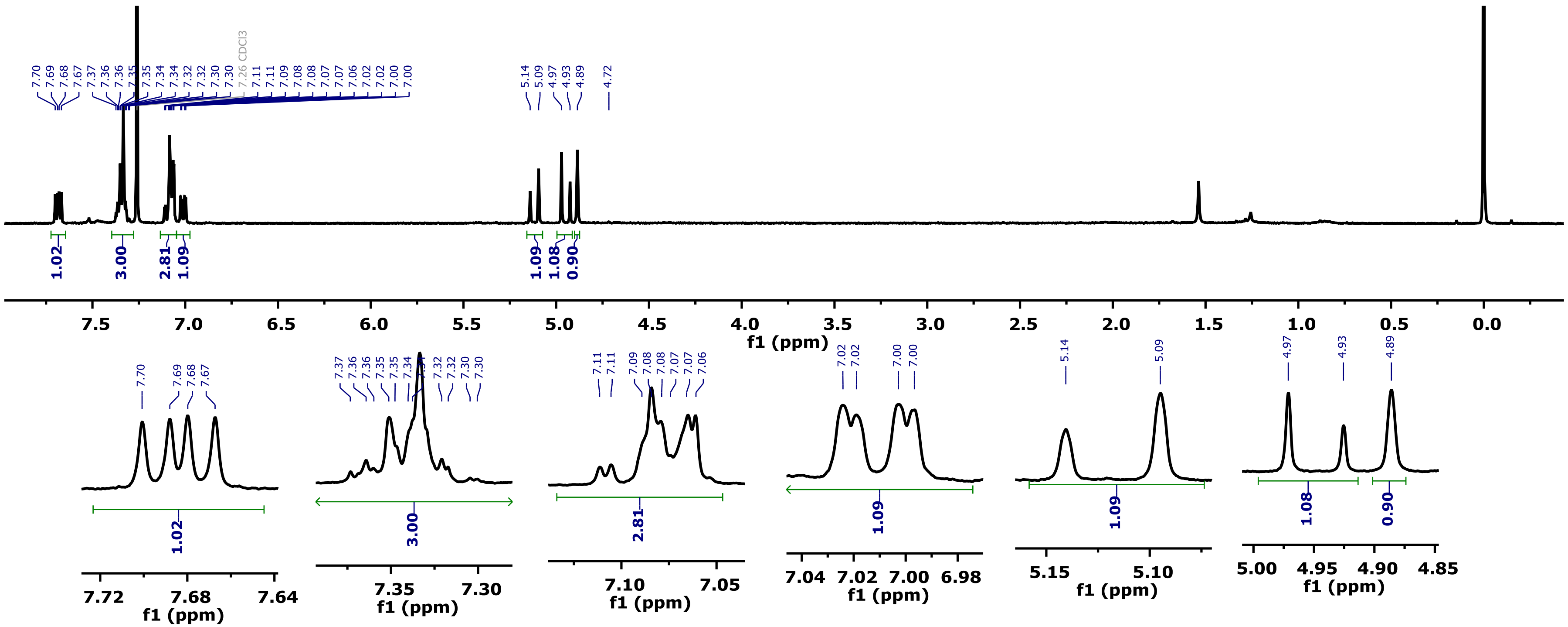

$^{13}C\{H\}$ NMR ($CDCl_3$, 100.6 MHz)

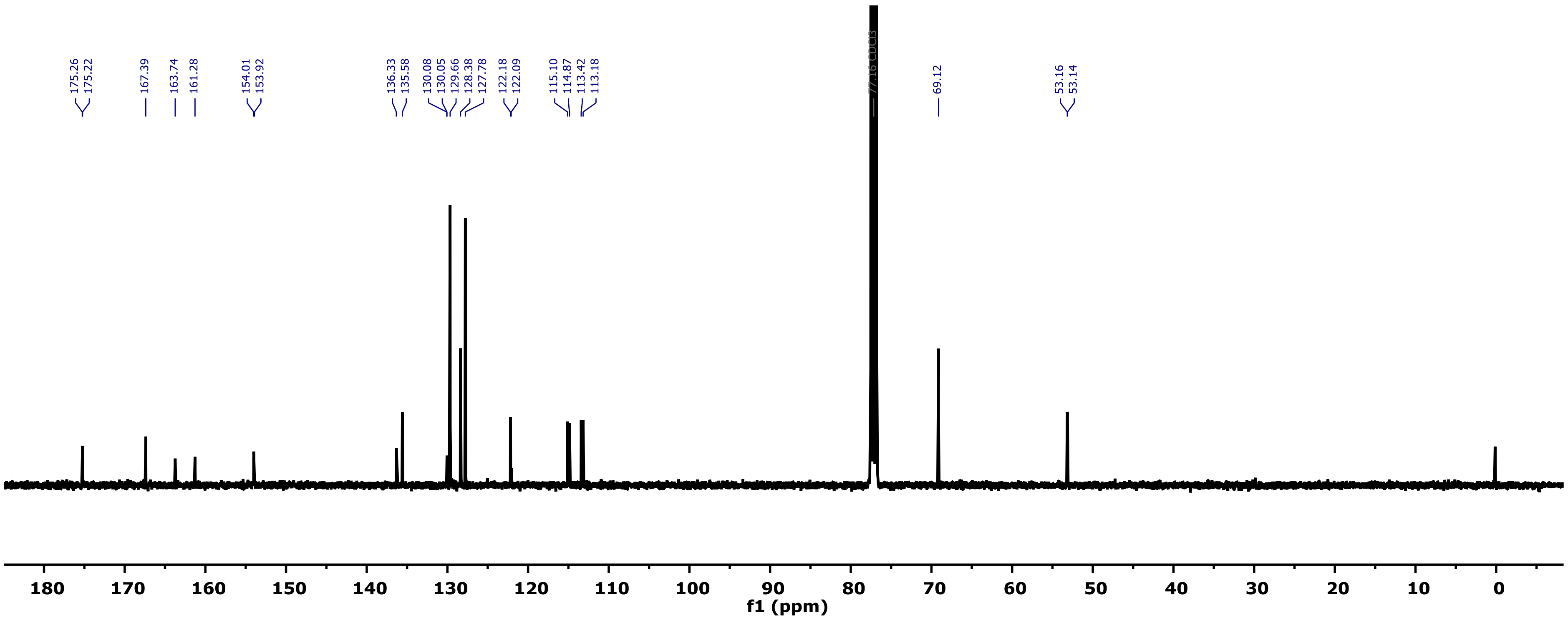

^{19}F NMR ($CDCl_3$, 376 MHz):

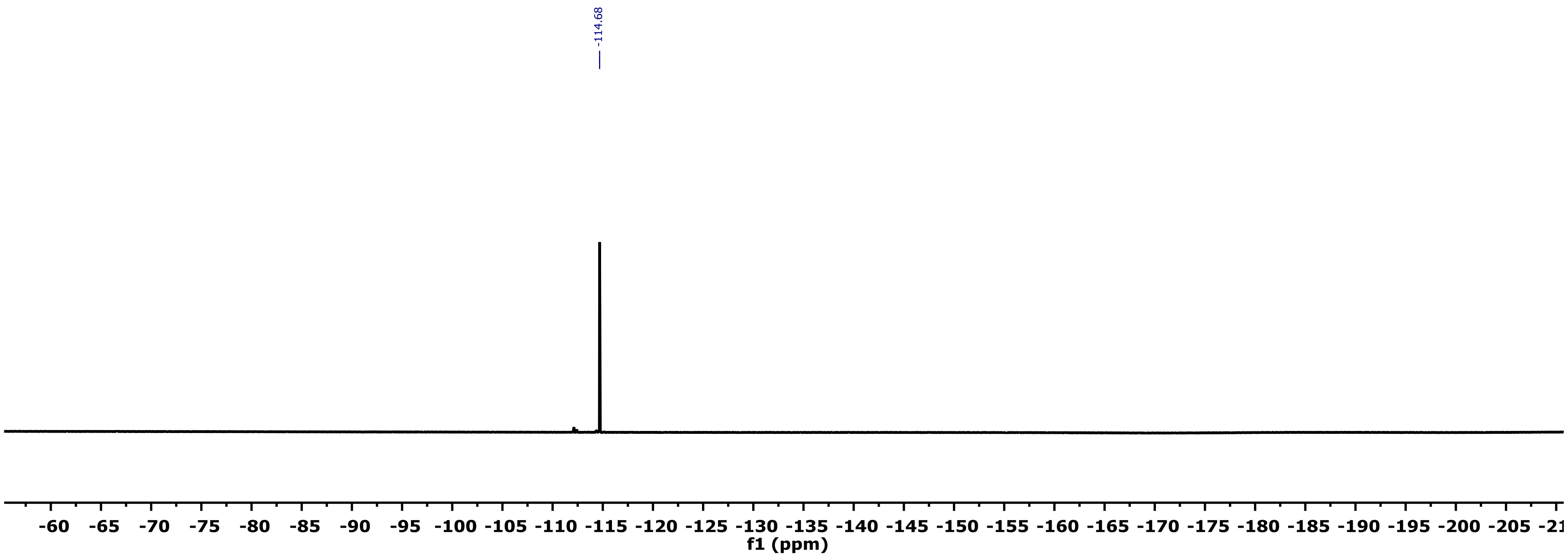

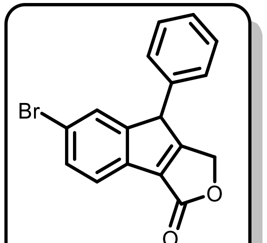

4l

^{1}H NMR (400.13 MHz, $CDCl_3$)

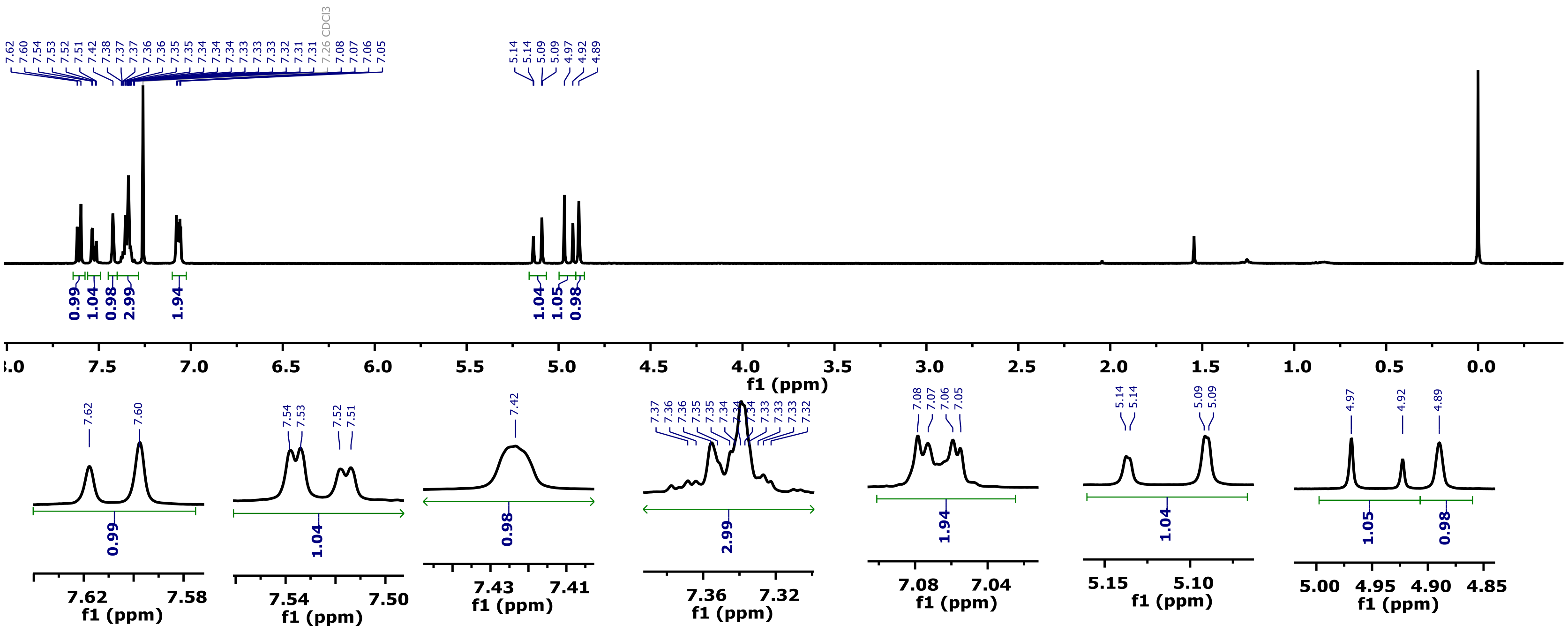

$^{13}C\{H\}$ NMR ($CDCl_3$, 100.6 MHz)

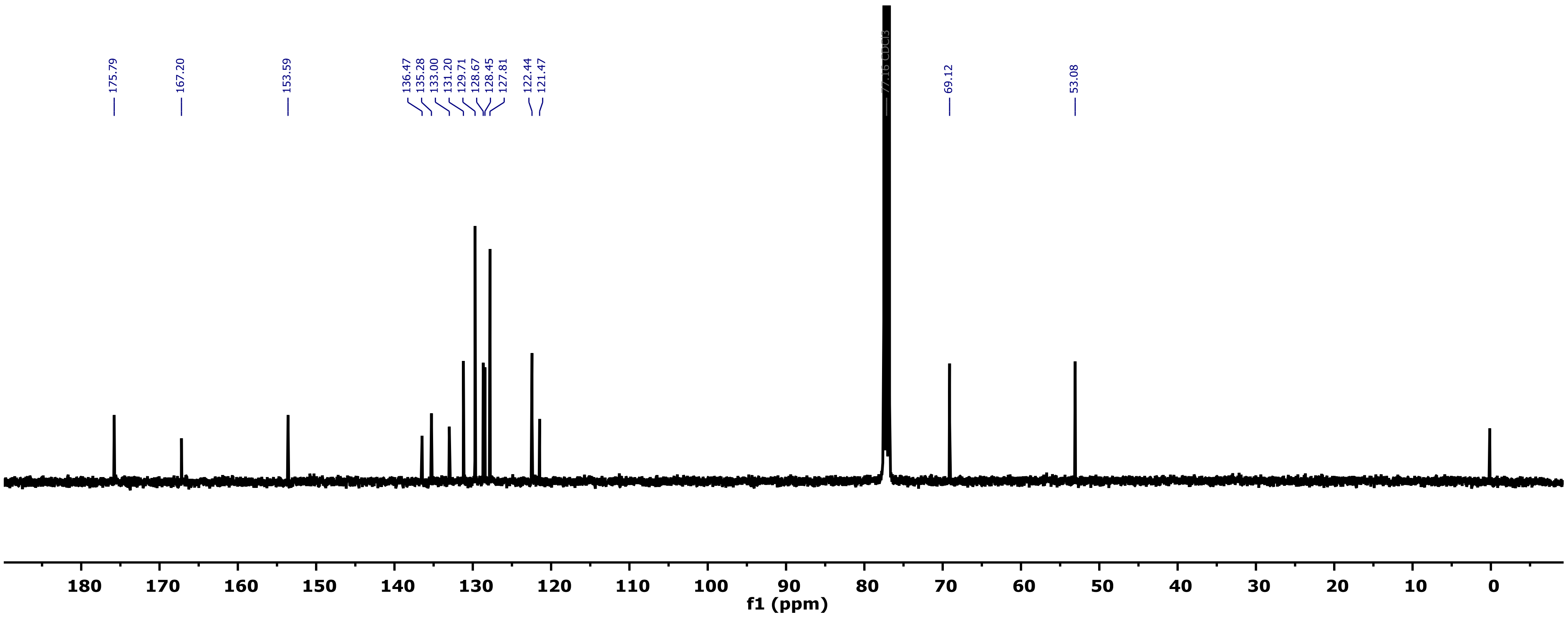

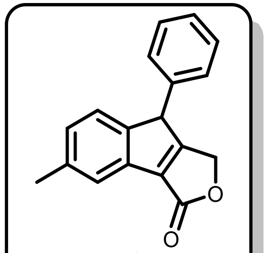

4m

^{1}H NMR (400.13 MHz, $CDCl_3$)

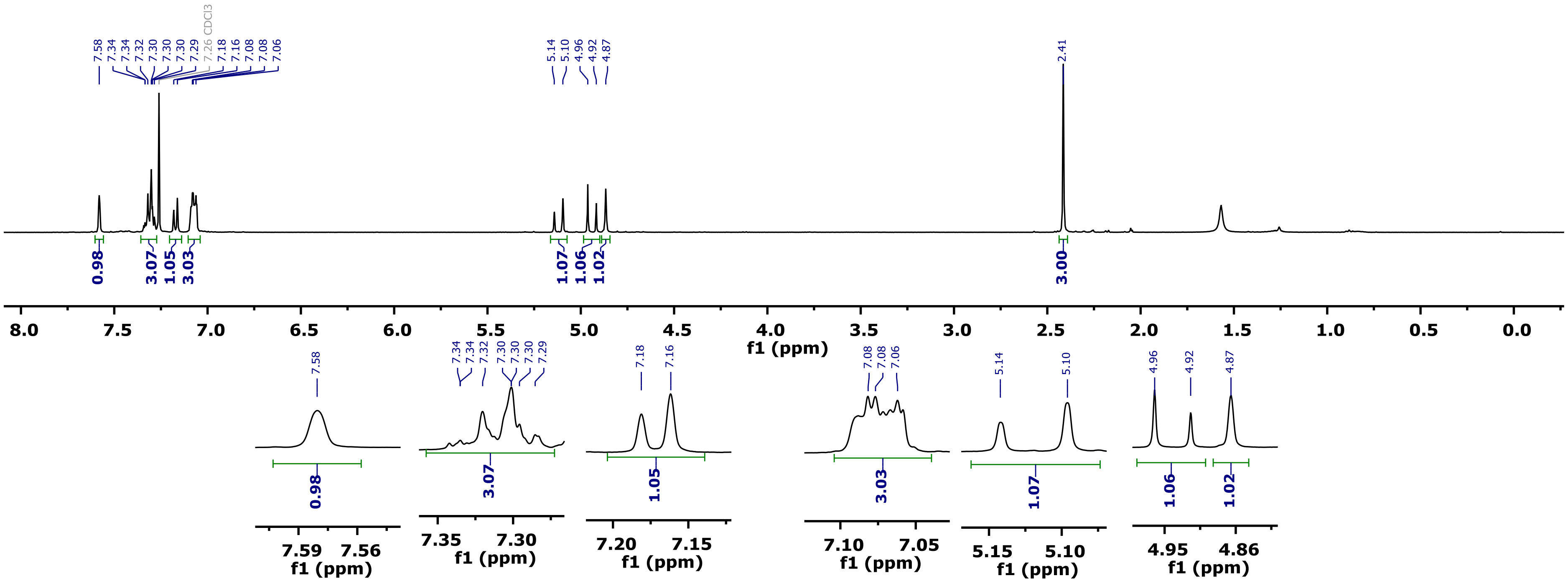

$^{13}C\{H\}$ NMR ($CDCl_3$, 100.6 MHz)

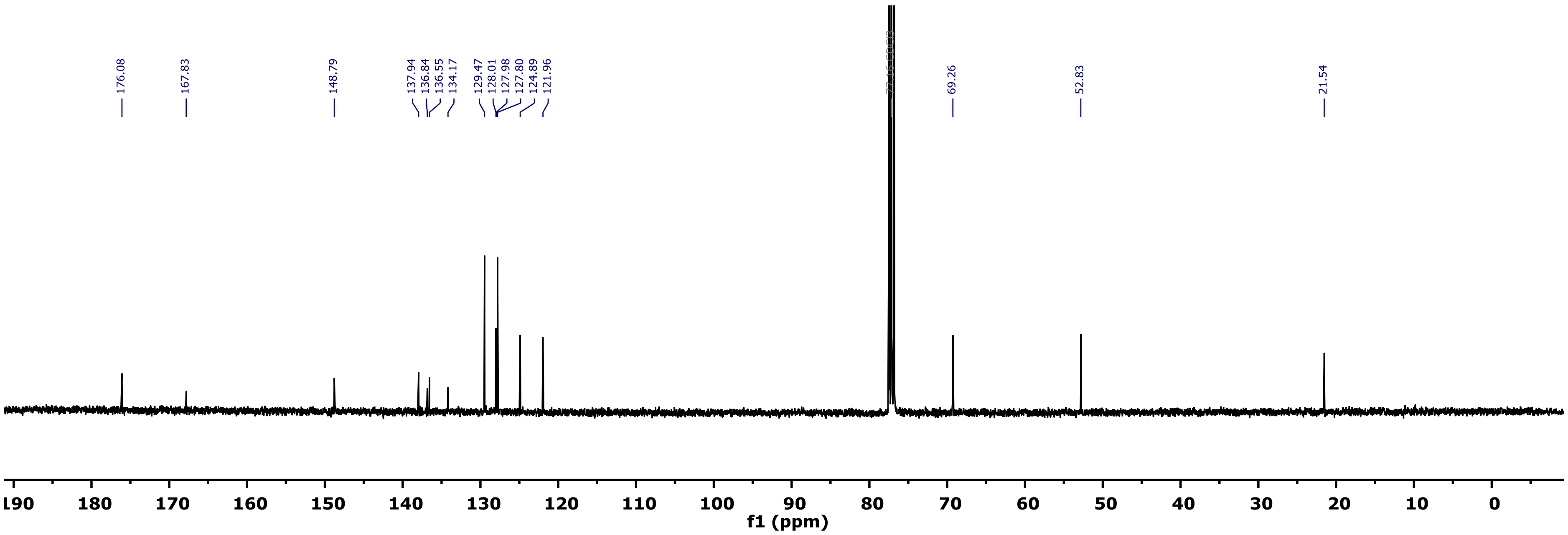

2D NMR SELECTIVE NOESY at 4.87 ppm ($CDCl_3$)

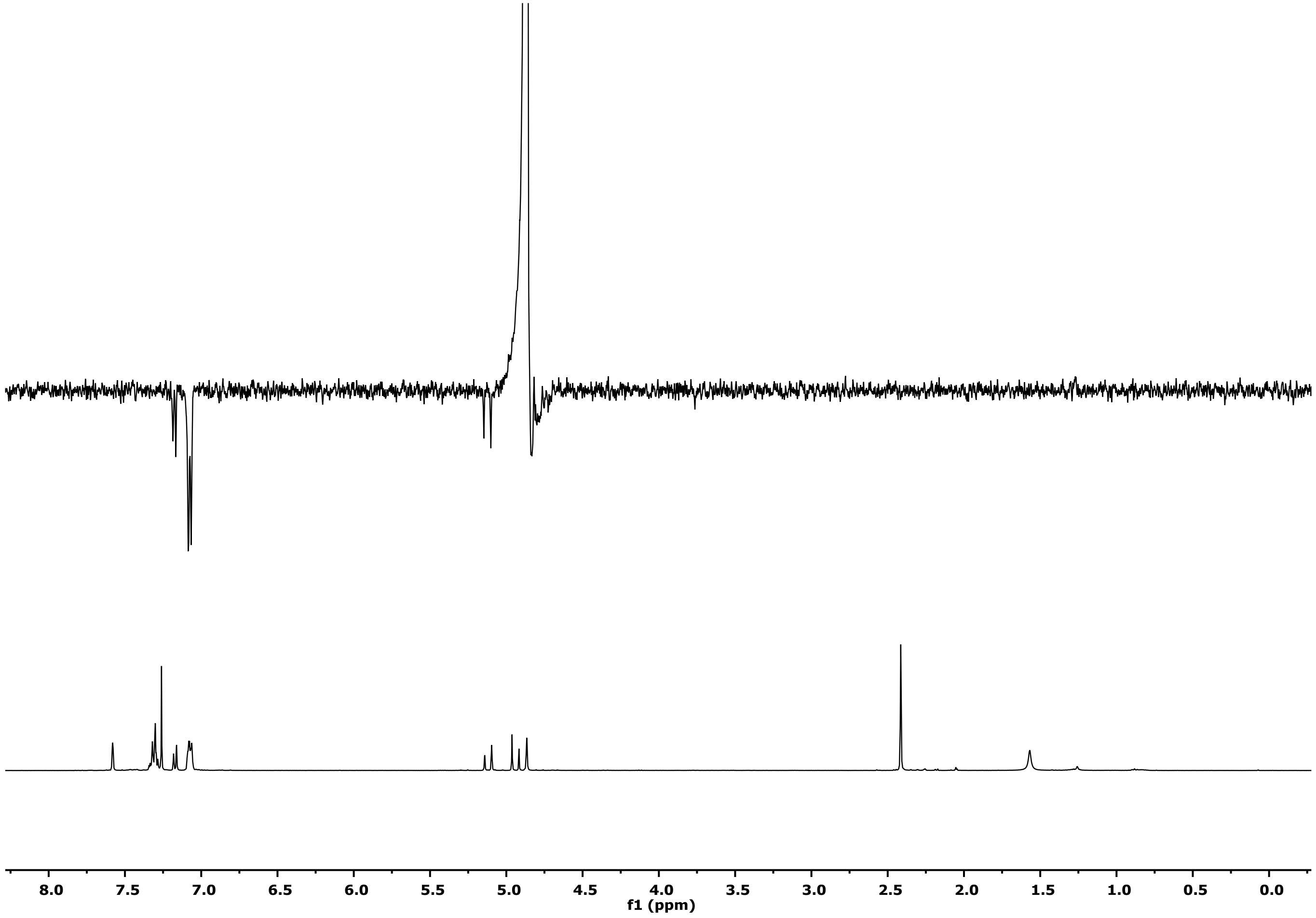

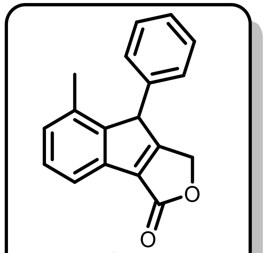

4m'

^{1}H NMR (400.13 MHz, $CDCl_3$)

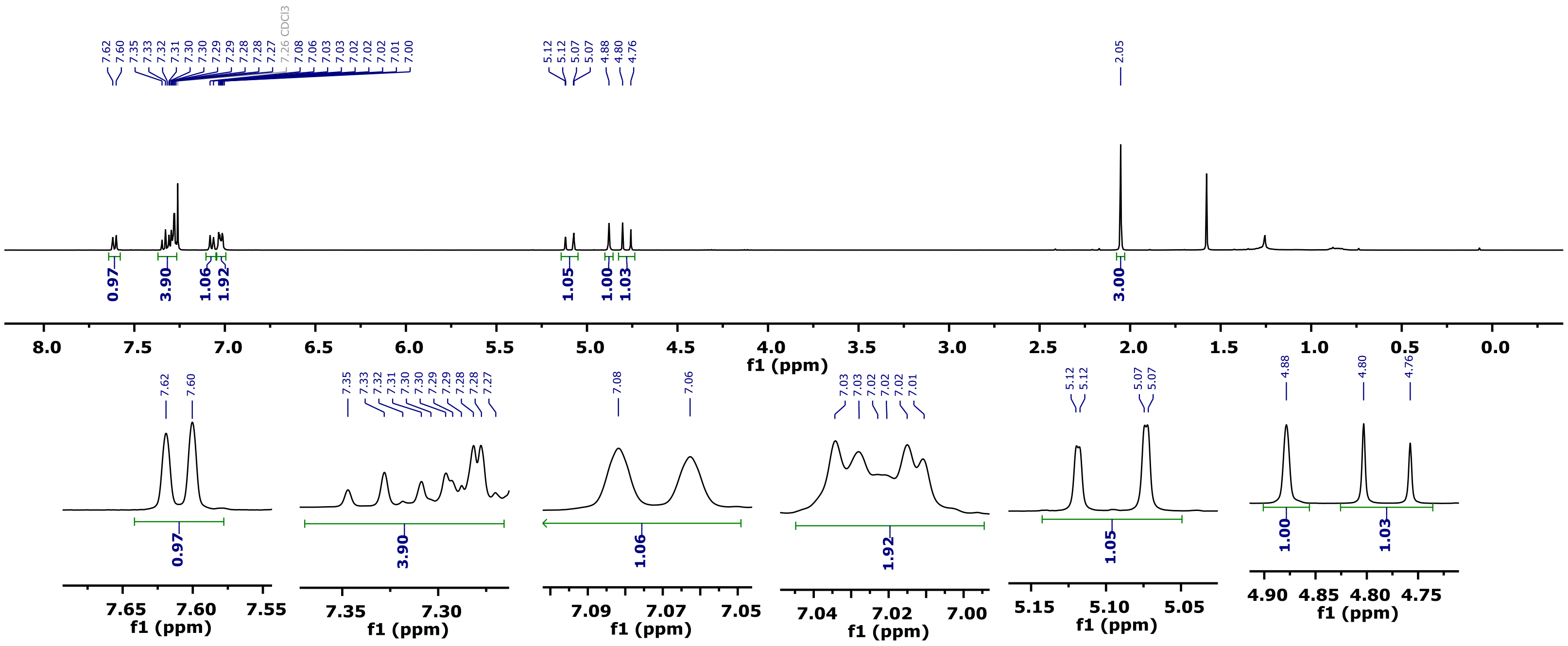

$^{13}C\{H\}$ NMR ($CDCl_3$, 100.6 MHz)

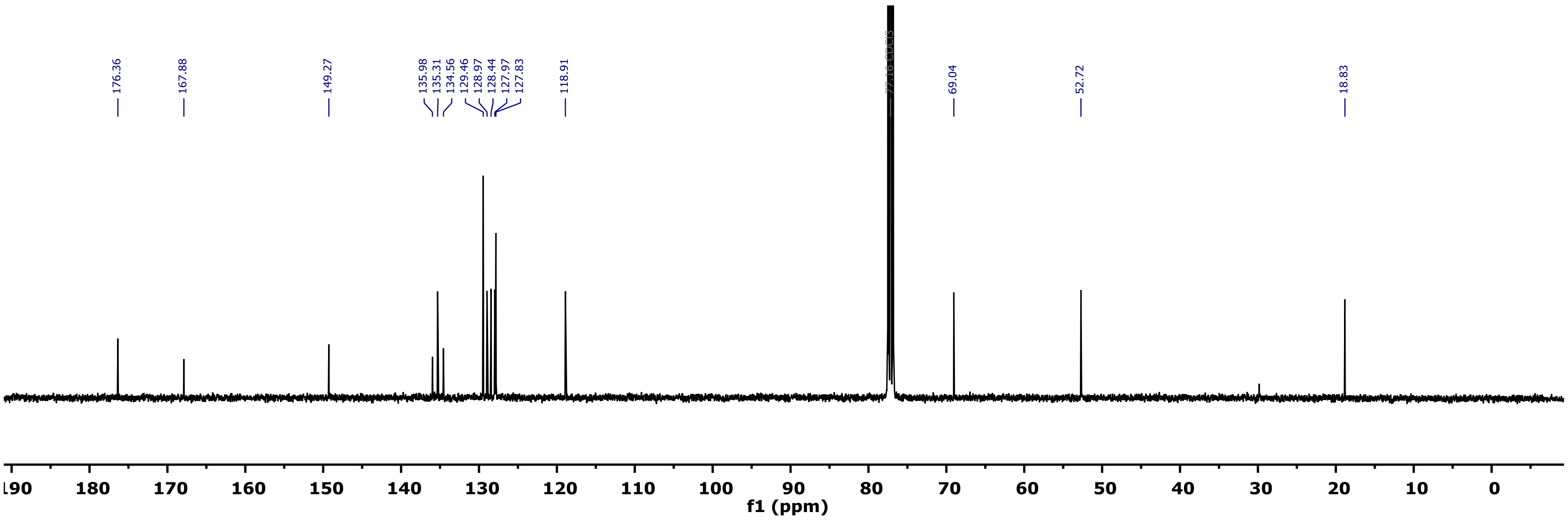

2D NMR SELECTIVE NOESY at 4.88 ppm ($CDCl_3$)

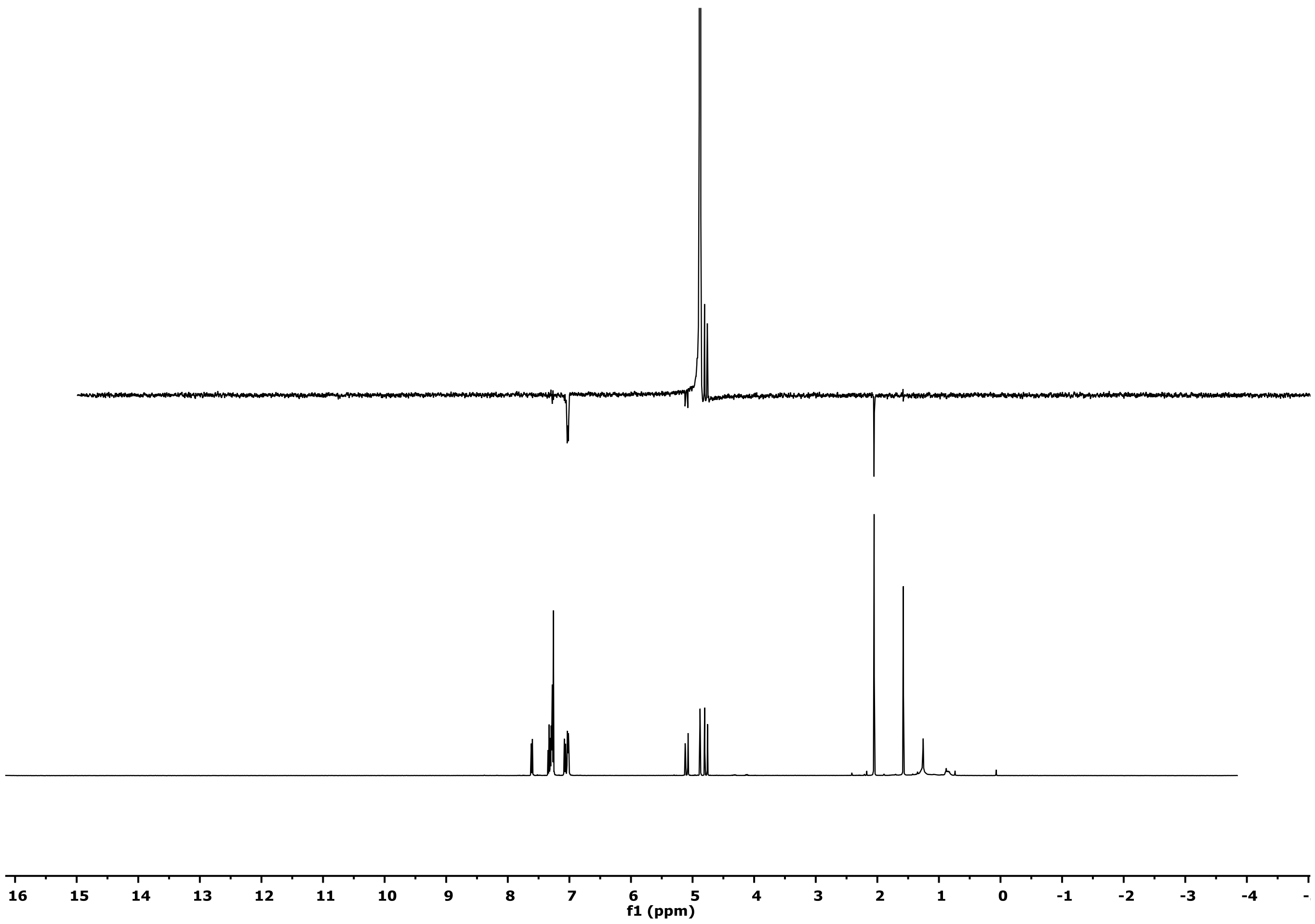

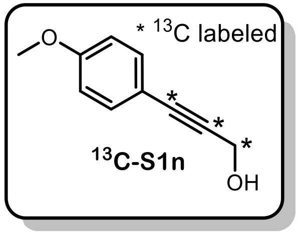

^{1}H NMR (400.13 MHz, $CDCl_3$)

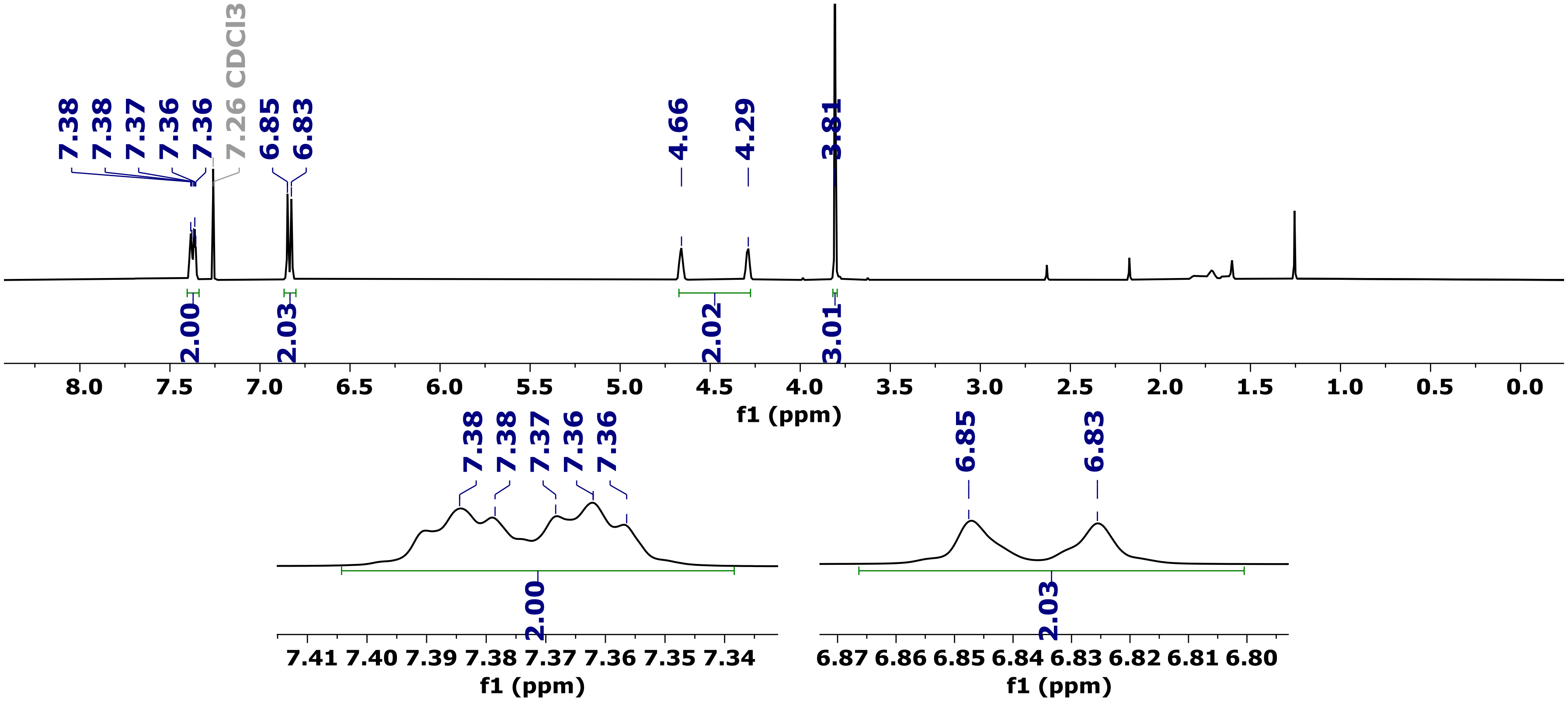

$^{13}C\{H\}$ NMR ($CDCl_3$, 100.6 MHz)

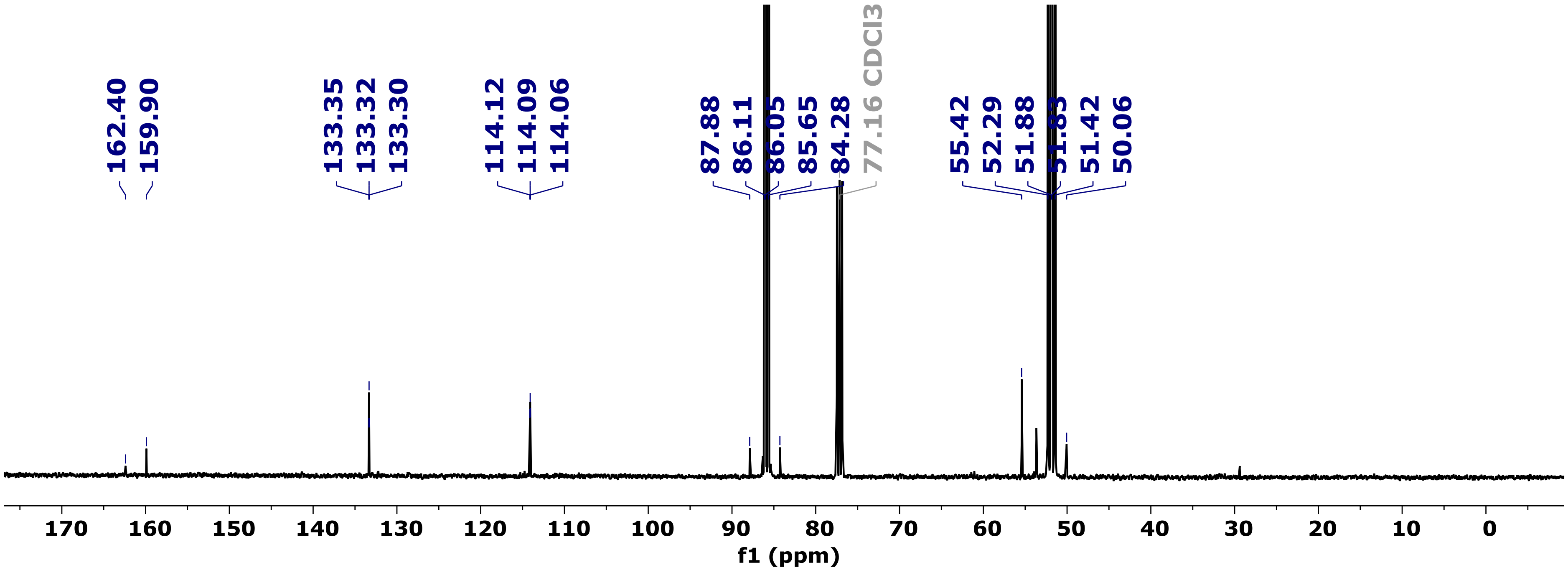

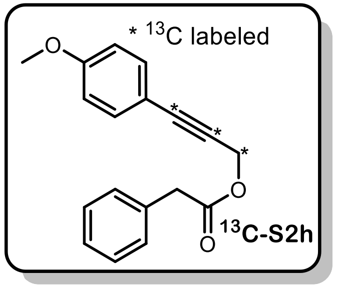

^{1}H NMR (400.13 MHz, $CDCl_3$)

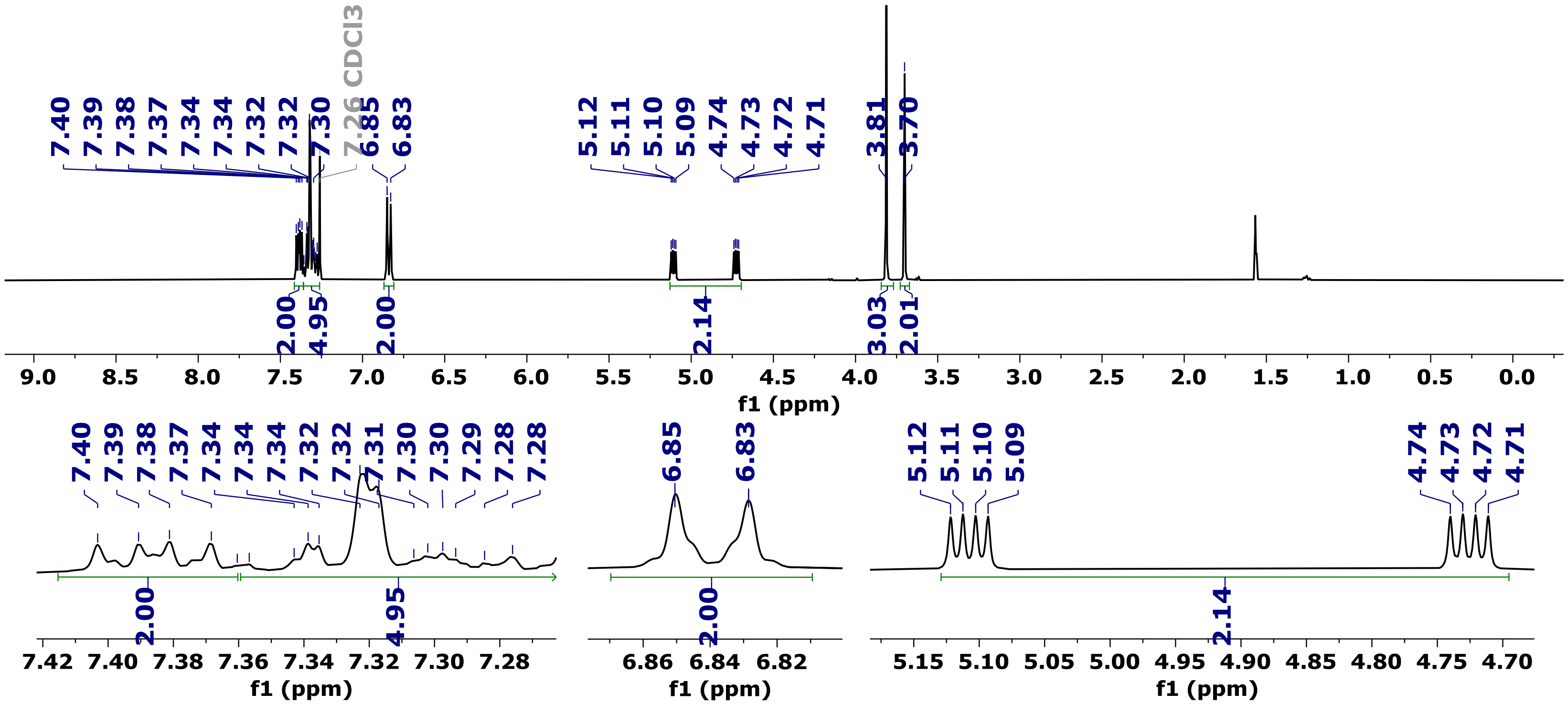

$^{13}C\{H\}$ NMR ($CDCl_3$, 100.6 MHz)

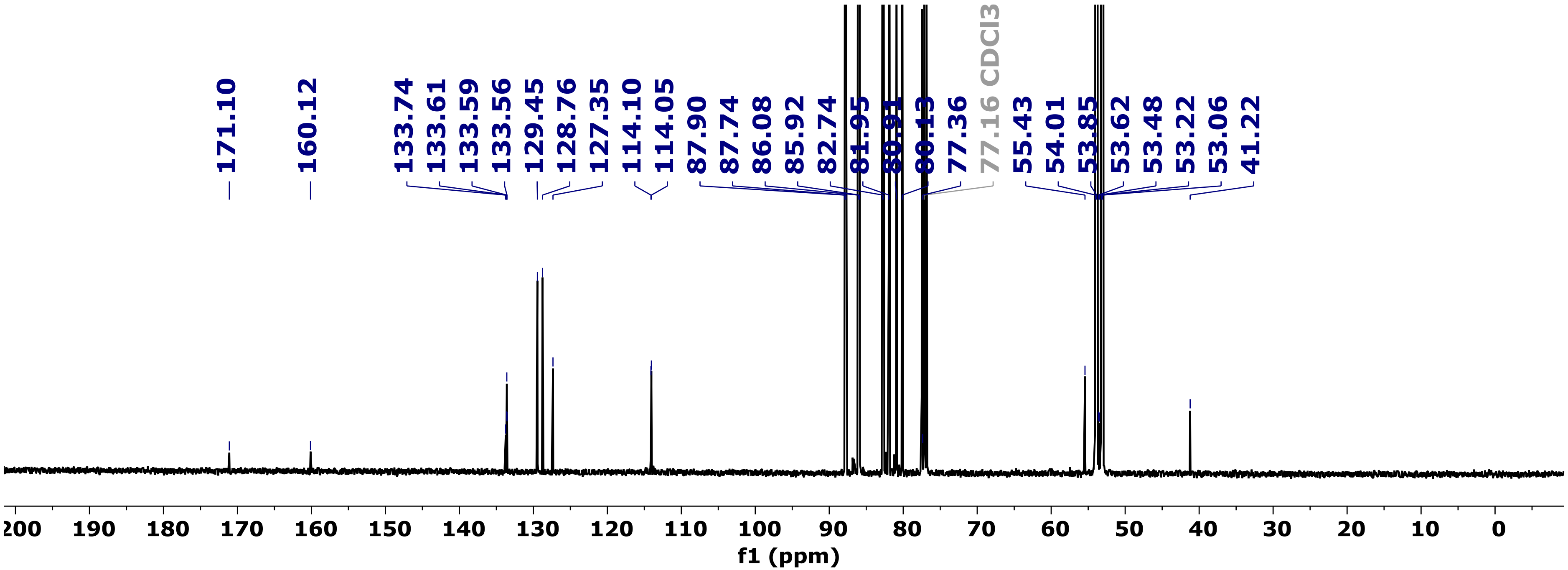

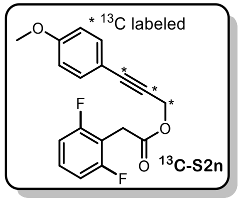

^{1}H NMR (400.13 MHz, $CDCl_3$)

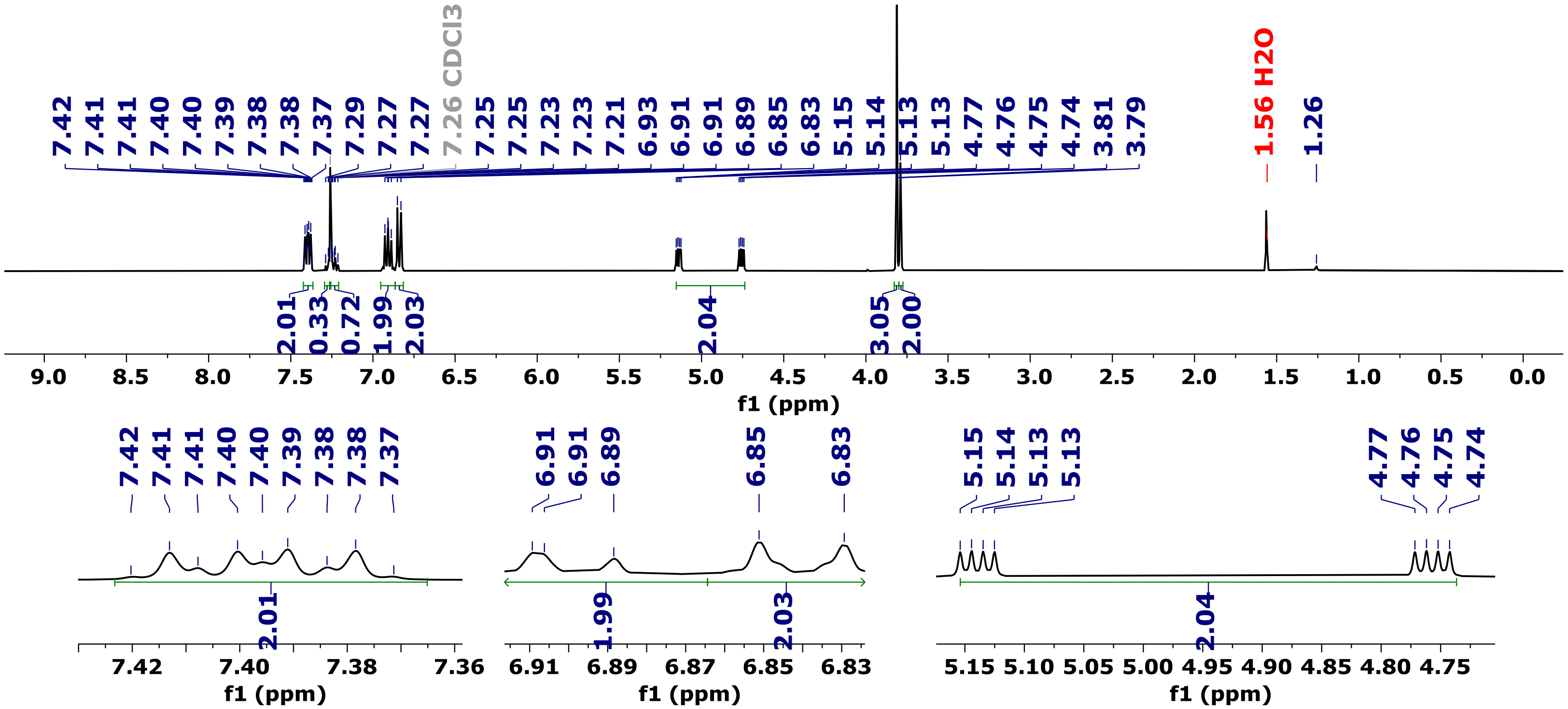

^{19}F NMR ($CDCl_3$, 376 MHz)

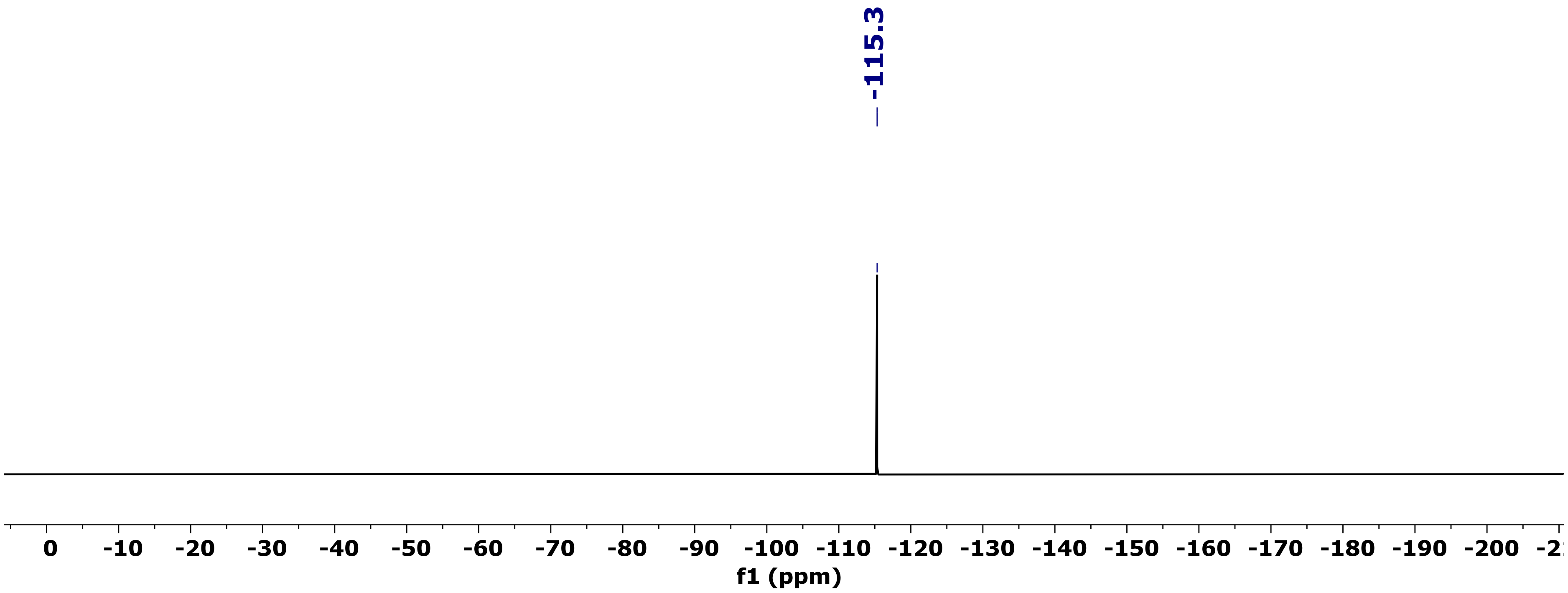

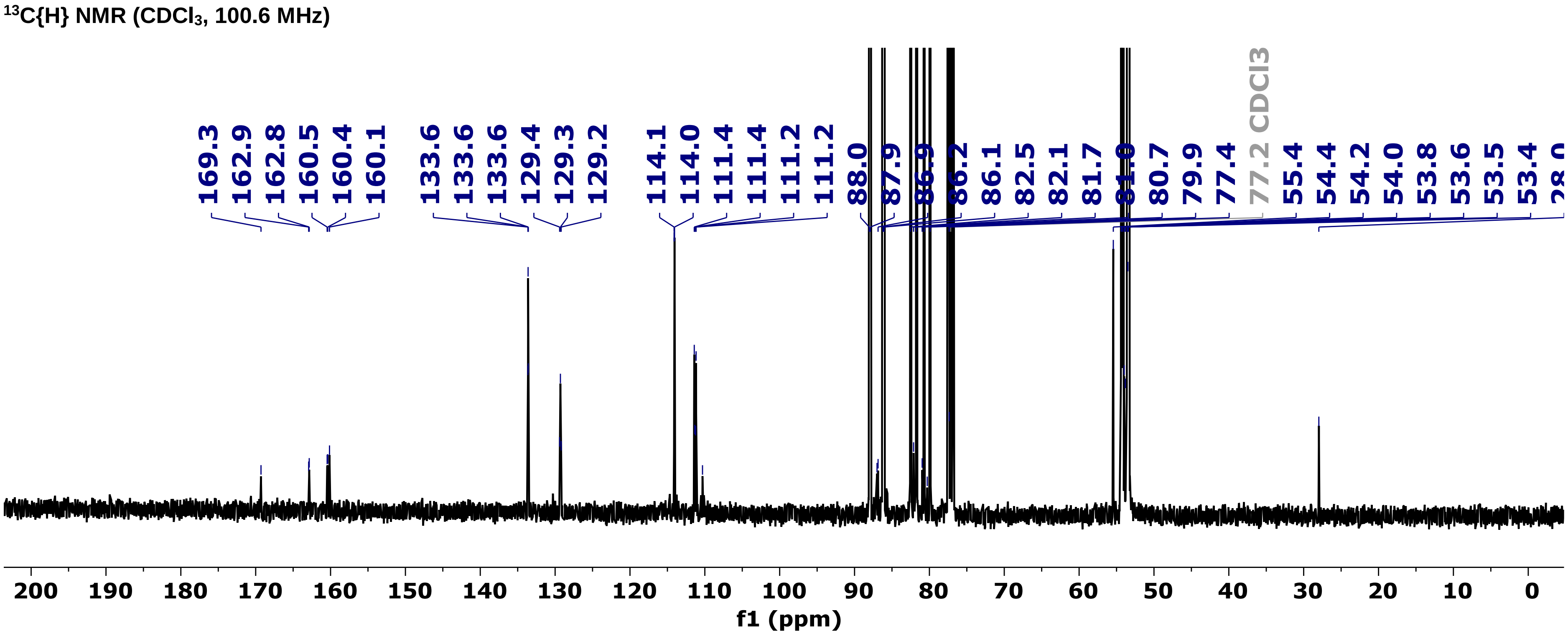
$^{13}C\{H\}$ NMR ($CDCl_3$, 100.6 MHz)
169.3
162.9
162.8
160.5
160.4
160.1
133.6
133.6
133.6
129.4
129.3
129.2
114.1
114.0
111.4
111.4
111.2
111.2
88.0
87.9
86.9
86.2
86.1
82.5
82.1
81.7
81.0
80.7
79.9
77.4
77.2 CDCl3
55.4
54.4
54.2
54.0
53.8
53.6
53.5
53.4
200
190
180
170
160
150
140
130
120
110
100
90
80
70
60
50
40
30
20
10
0
f1 (ppm)

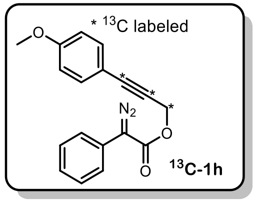

^{1}H NMR (400.13 MHz, $CDCl_3$)

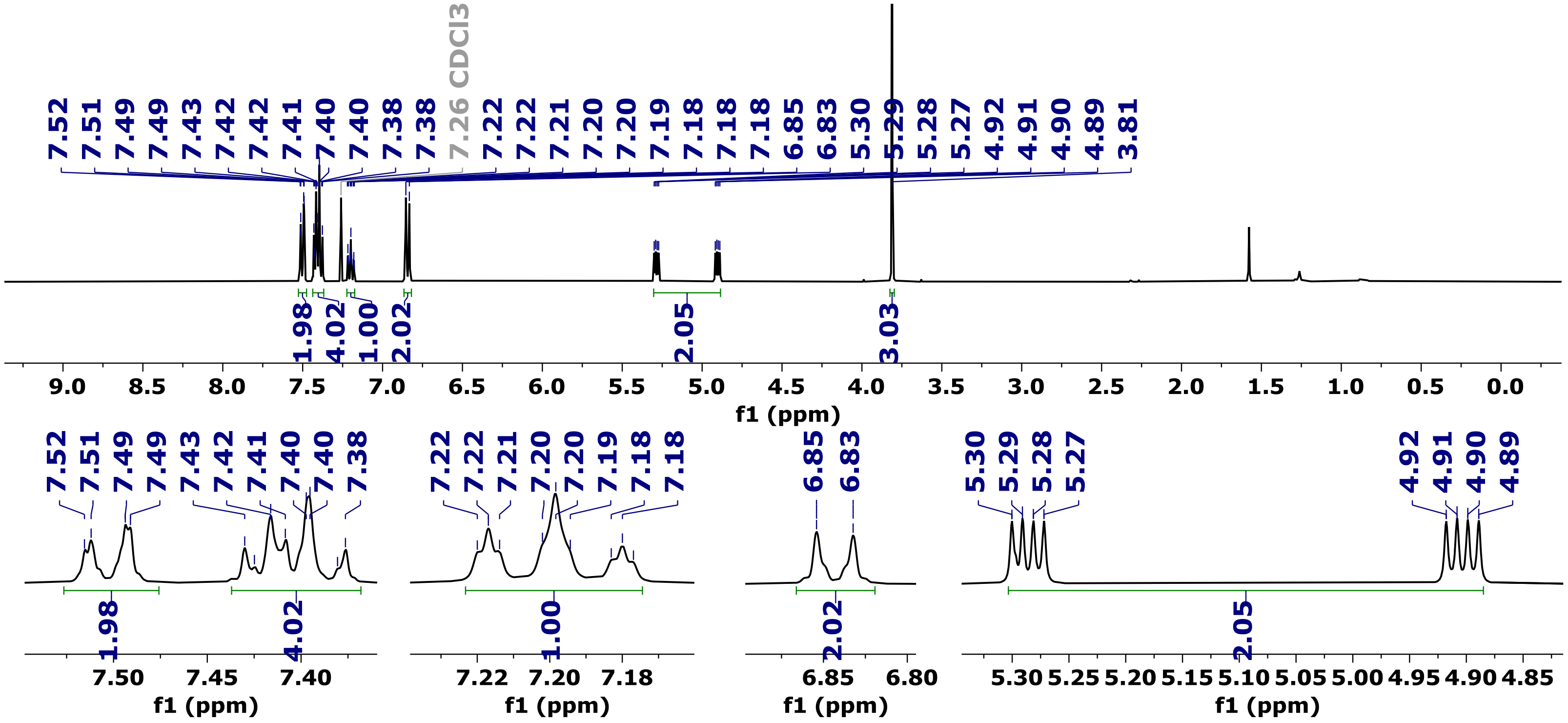

$^{13}C\{H\}$ NMR ($CDCl_3$, 100.6 MHz)

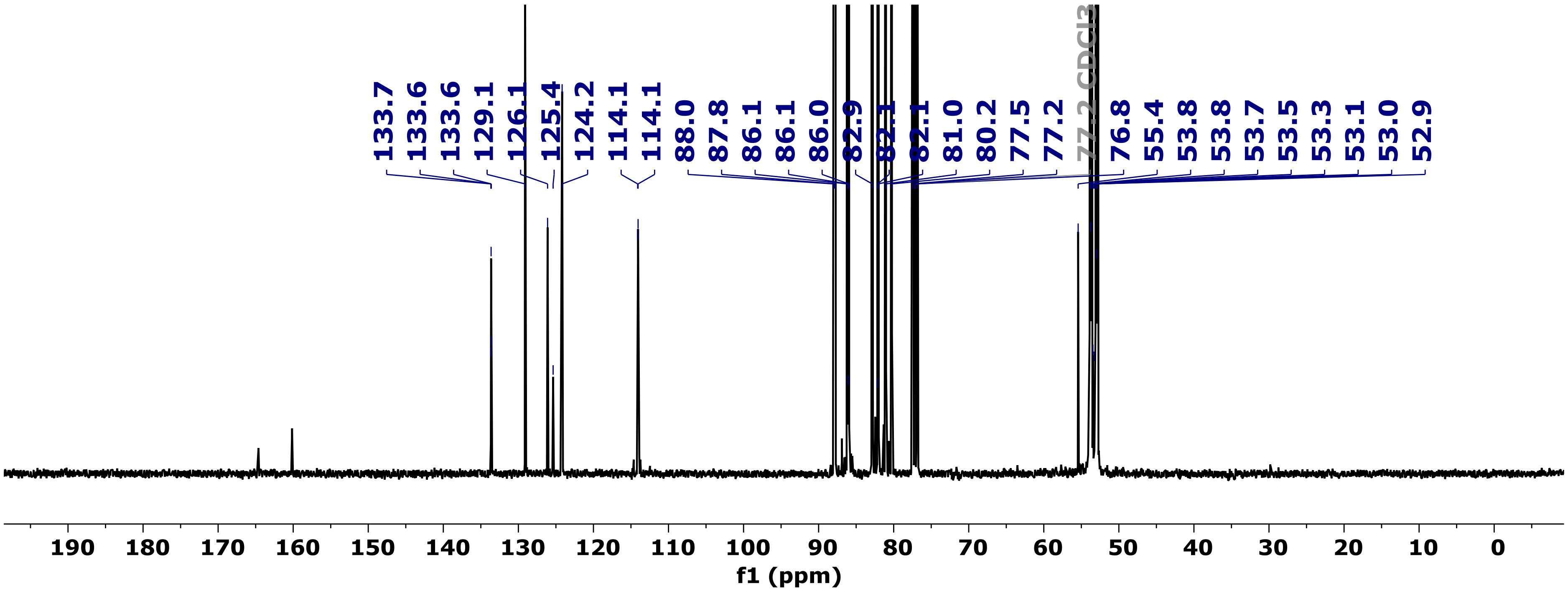

HMBC

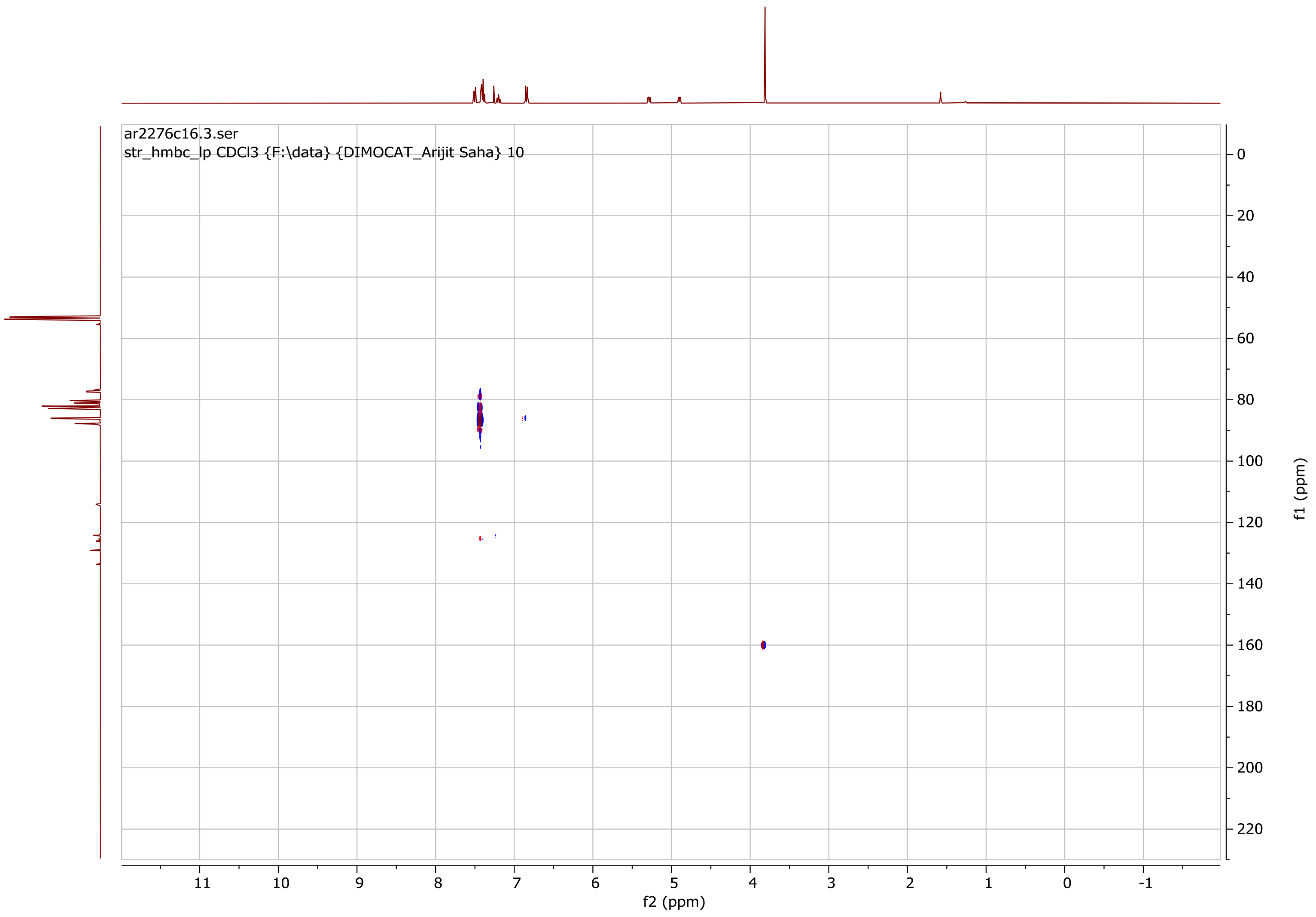

ar2276c16.3.ser
str_hmbc_lp CDCl3 {F:\data} {DIMOCAT_Arijit Saha} 10
f2 (ppm)
f1 (ppm)

HSQCED

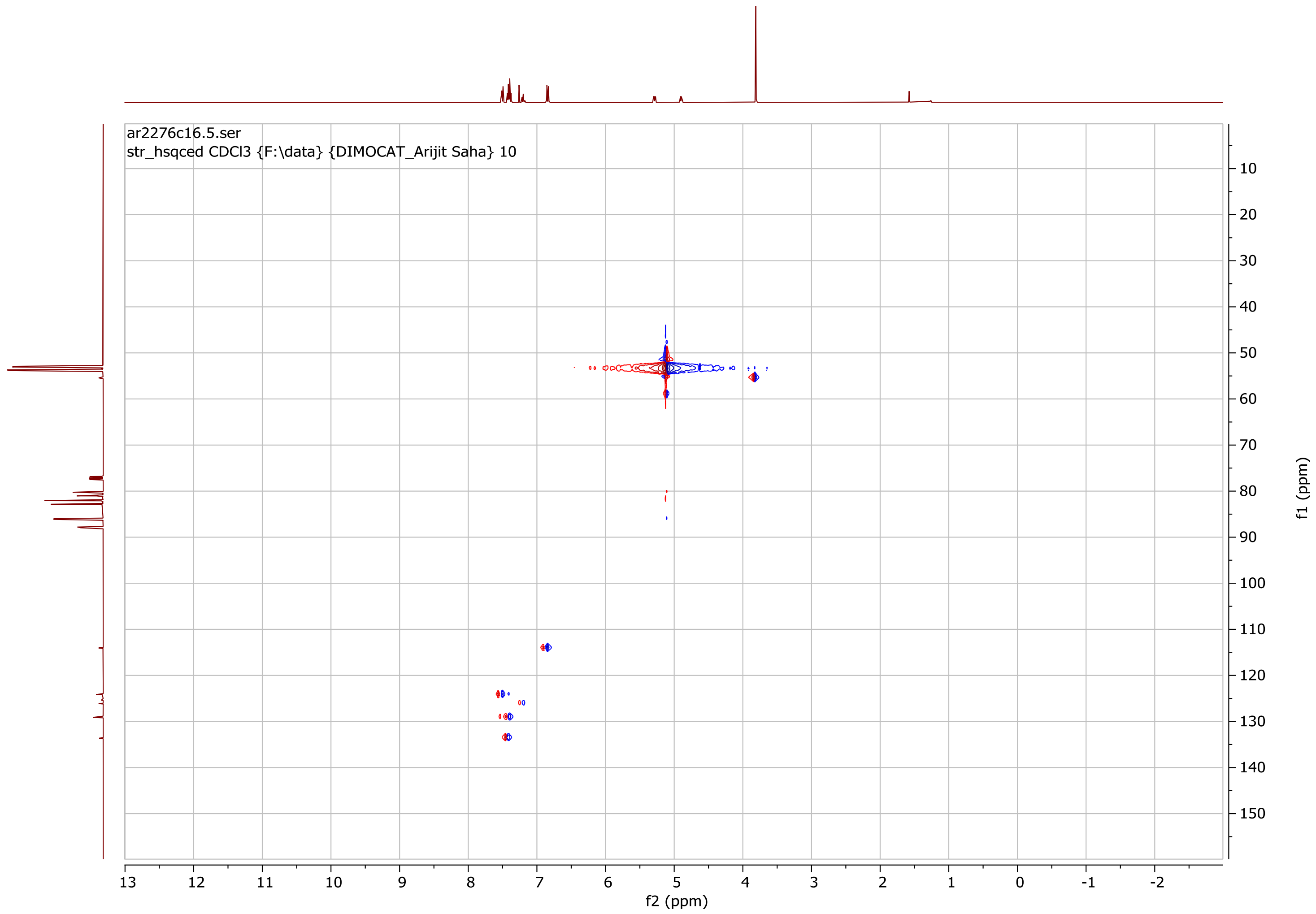

ar2276c16.5.ser
str_hsqced CDCl3 {F:\data} {DIMOCAT_Arijit Saha} 10
f2 (ppm)
f1 (ppm)

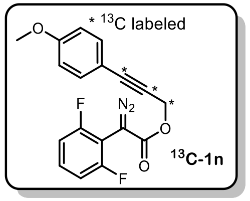

^{1}H NMR (400.13 MHz, $CDCl_3$)

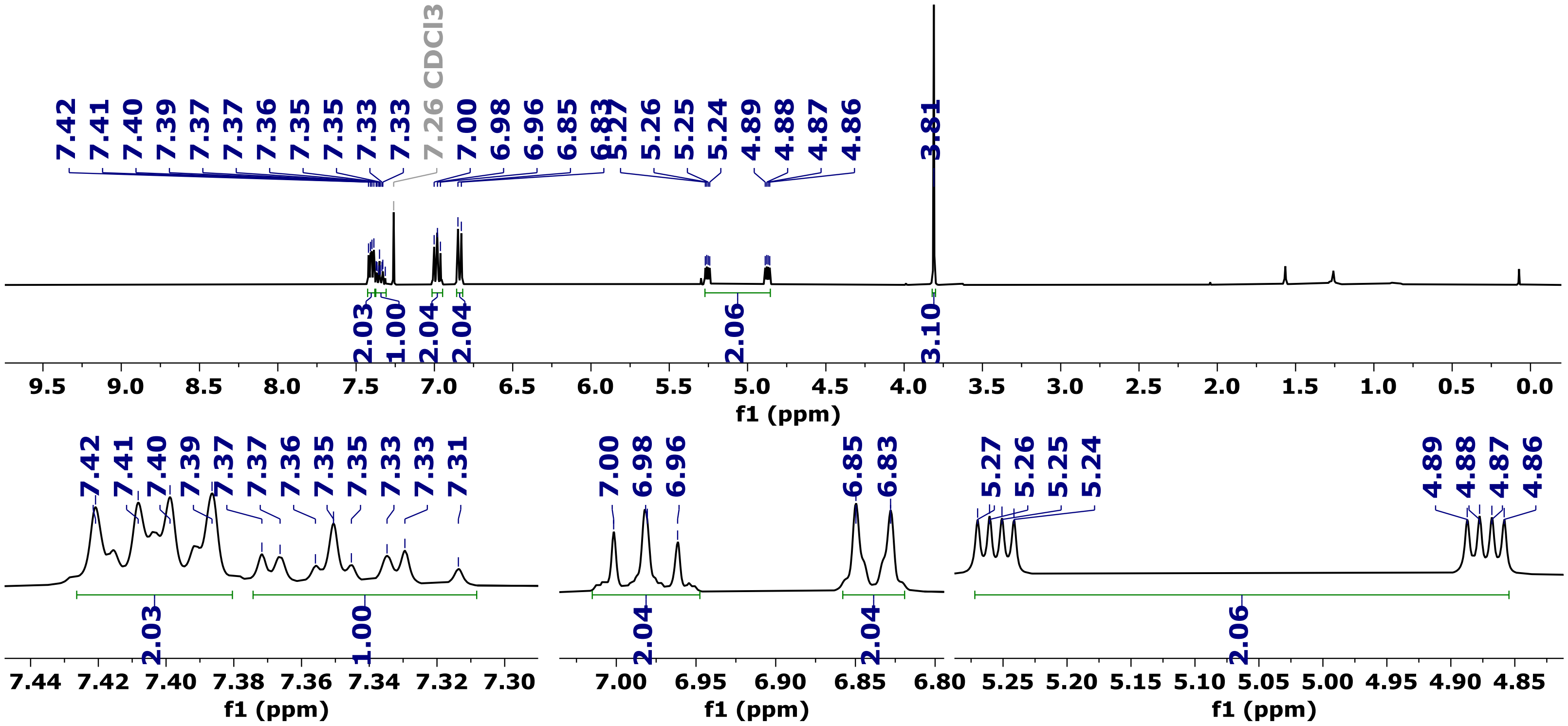

^{19}F NMR ($CDCl_3$, 376 MHz)

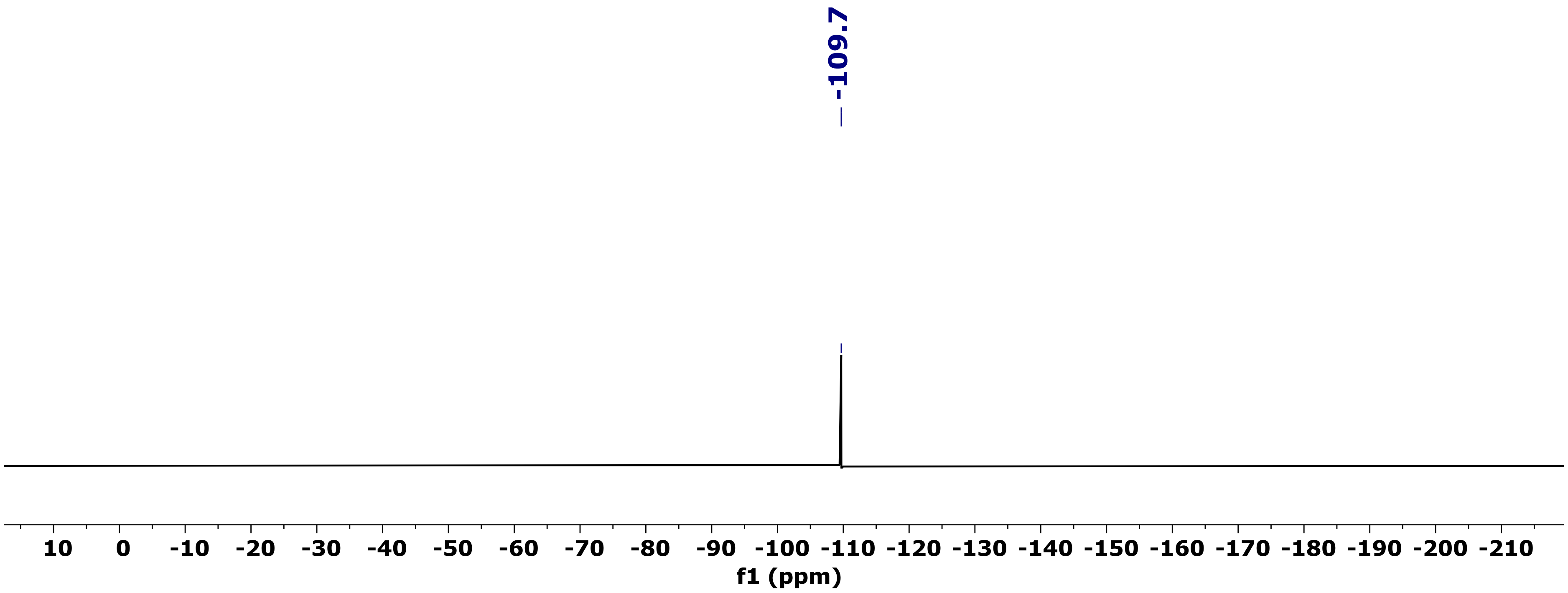

$^{13}C\{H\}$ NMR ($CDCl_3$, 100.6 MHz)

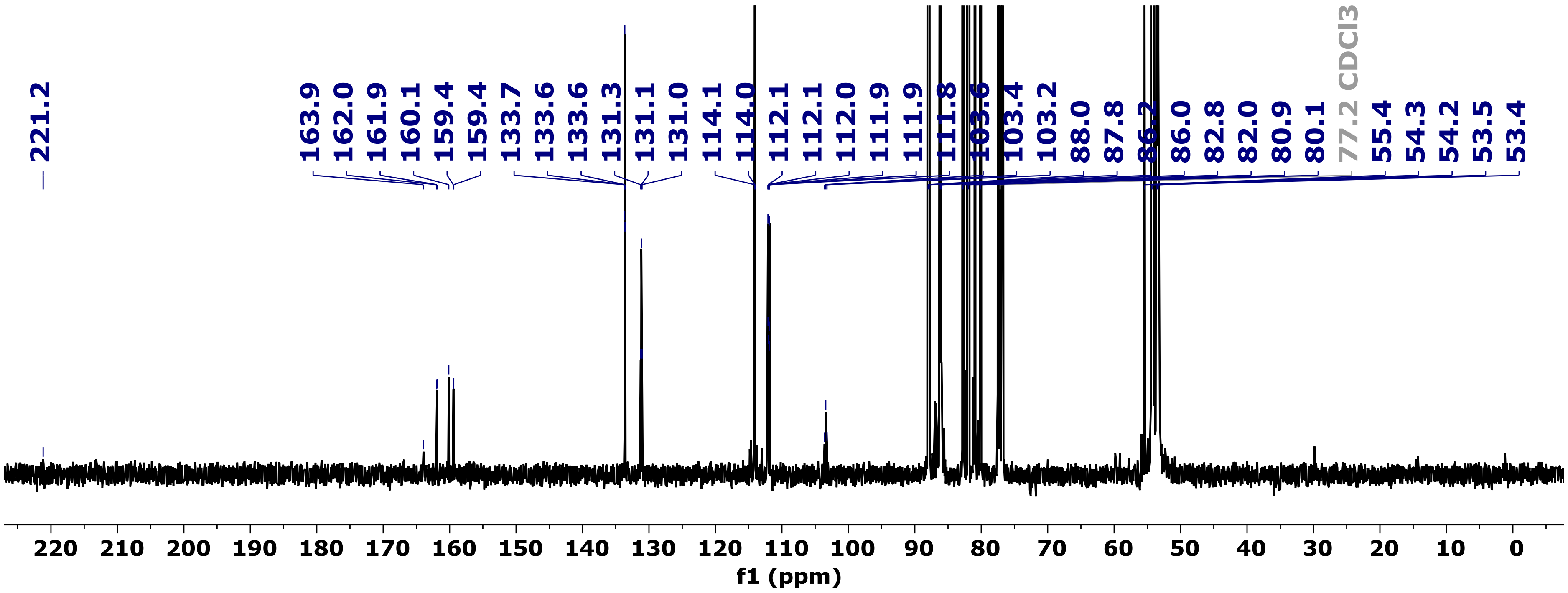

HMBC

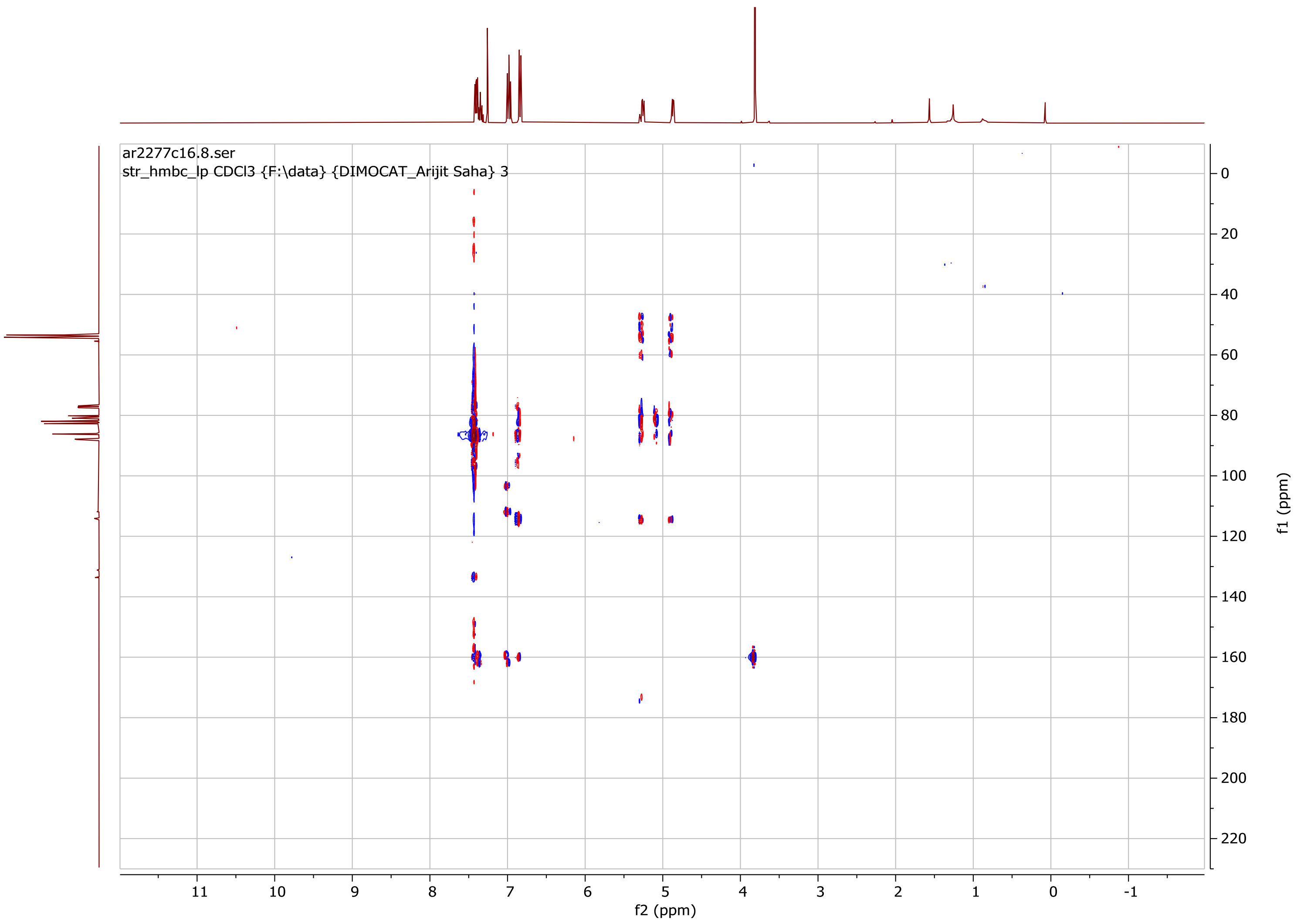

ar2277c16.8.ser
str_hmbc_lp CDCl3 {F:\data} {DIMOCAT_Arijit Saha} 3
11 10 9 8 7 6 5 4 3 2 1 0 -1
f2 (ppm)
0 20 40 60 80 100 120 140 160 180 200 220
f1 (ppm)

HSQCED

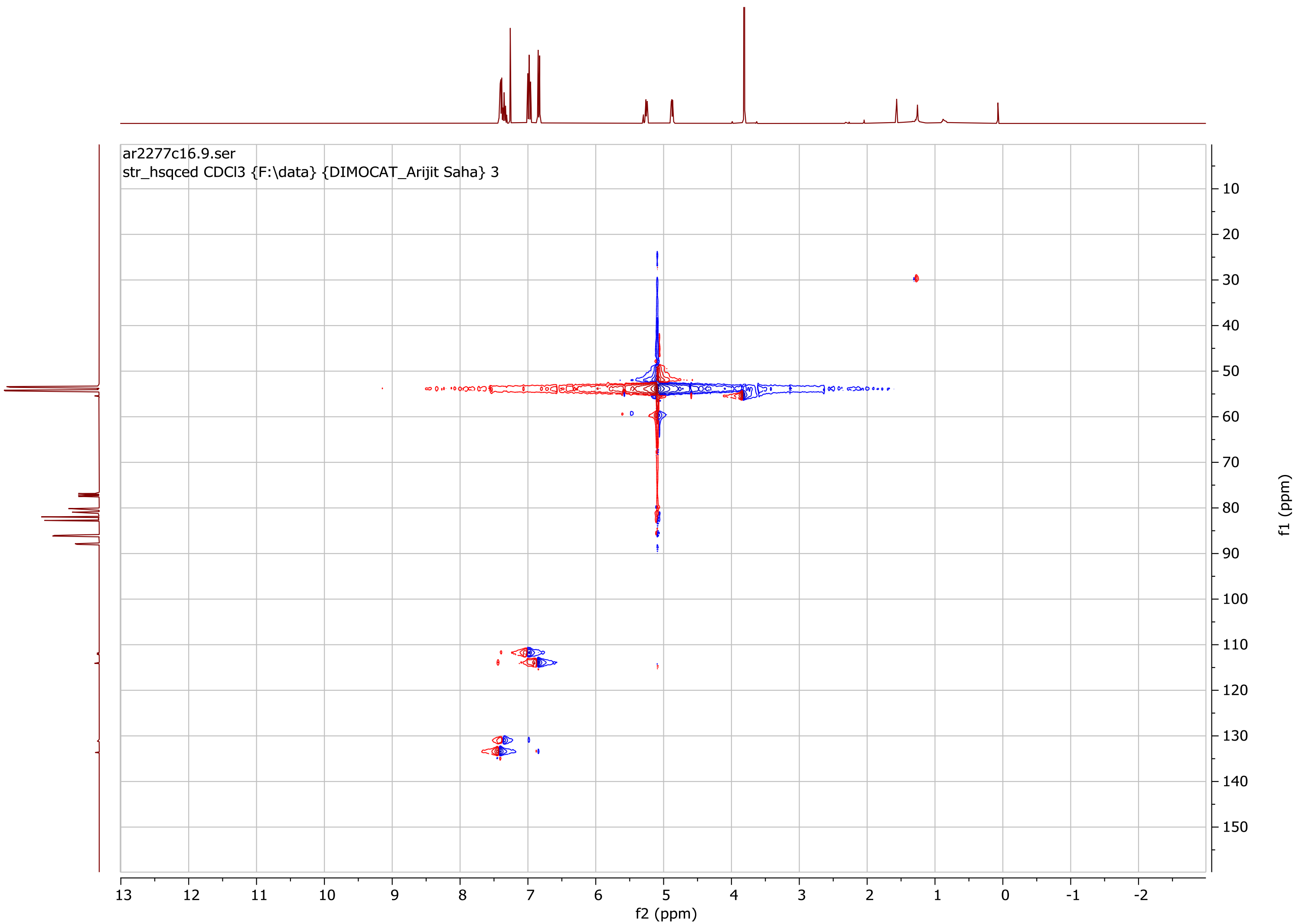

ar2277c16.9.ser
str_hsqced CDCl3 {F:\data} {DIMOCAT_Arijit Saha} 3
13 12 11 10 9 8 7 6 5 4 3 2 1 0 -1 -2
f2 (ppm)
10 20 30 40 50 60 70 80 90 100 110 120 130 140 150
f1 (ppm)
